# Hopf bifurcation in an age-structured predator–prey system with Beddington–DeAngelis functional response and constant harvesting

**DOI:** 10.1007/s00285-024-02070-3

**Published:** 2024-04-04

**Authors:** San-Xing Wu, Zhi-Cheng Wang, Shigui Ruan

**Affiliations:** 1 School of Mathematics and Statistics, Lanzhou University, Lanzhou, 730000 Gansu People’s Republic of China; 2 Department of Mathematics, University of Miami, Coral Gables, FL 33146 USA

**Keywords:** Age-structured predator–prey model, Abstract Cauchy problem, Hopf bifurcation, Center manifold theorem, Normal form theory, 34K18, 35K90, 37L10, 65L03, 92D25

## Abstract

In this paper, an age-structured predator–prey system with Beddington–DeAngelis (B–D) type functional response, prey refuge and harvesting is investigated, where the predator fertility function *f*(*a*) and the maturation function $\beta (a)$ are assumed to be piecewise functions related to their maturation period $\tau$. Firstly, we rewrite the original system as a non-densely defined abstract Cauchy problem and show the existence of solutions. In particular, we discuss the existence and uniqueness of a positive equilibrium of the system. Secondly, we consider the maturation period $\tau$ as a bifurcation parameter and show the existence of Hopf bifurcation at the positive equilibrium by applying the integrated semigroup theory and Hopf bifurcation theorem. Moreover, the direction of Hopf bifurcation and the stability of bifurcating periodic solutions are studied by applying the center manifold theorem and normal form theory. Finally, some numerical simulations are given to illustrate of the theoretical results and a brief discussion is presented.

## Introduction

Nonlinear dynamics of predator–prey systems have been investigated extensively in recent years. Most existing studies have focused on the influence of stage structure, different functional response functions, Allee effect and diffusion (Aiello and Freedman 1990; Fang et al. 2016; Liu and Wang 2011; Guin et al. 2021; Wu and Meng 2021; Yang and Wang 2020b; Zhang et al. 2022; Zhang and Wang 2015). However, some immature populations do not have most survival skills due to environmental and age limitations, so they have no ability to obtain food independently or migrate to other places. Therefore, some researchers have paid attention to the effects of population age on predator–prey interactions, which has become an important research topic in biology and ecology. Cushing and Saleem (1982) studied the following predator–prey model, in which the predator population is assumed to have an age structure:

1.1 $$\begin{aligned} \left\{ \begin{aligned}&\frac{\partial u(t,a)}{\partial t}+\frac{\partial u(t,a)}{\partial a}=-\mu u(t,a),\\&\frac{\text{ d } R(t)}{\text{ d } t}=rR(t)\Big (1-\frac{R(t)}{K}\Big )-g(R(t),P(t)),\\&u(t,0)=\int _{0}^{+\infty }b\beta (a)h(R(t),P(t))u(t,a)\text{ d }a,\\&P(t)=\int _{0}^{+\infty }u(t,a)\text{ d }a,\\&u(0,a)=u_0\in L_+^{1}((0,+\infty ),\mathbb {R}),\;R(0)=R_0\ge 0, \end{aligned} \right. \end{aligned}$$

where *u*(*t*, *a*) is the density of the predator population at time *t* with age *a*; *R*(*t*) is the density of the prey population at time *t*; $P(t)=\int _{0}^{+\infty }u(t,a)\text{ d }a$ is the total number of the predator population of all age groups at time *t*; *r* and $\mu$ are the intrinsic growth rate of the prey and the death rate of predators, respectively; *K* is the environmental carrying capacity for the prey; The function $h(R(t),P(t))=R(t)(cP(t)+1)$, as the fecundity response function, is assumed to be twice continuously differentiable for $R(t),P(t)\ge 0$ and *c* is a positive constant. The predation response function $g(R(t),P(t))=R(t)P(t)$ is also assumed to be twice continuously differentiable for $R(t),P(t)\ge 0$. Cushing and Saleem (1982) mainly studied the existence, stability and destabilization of equilibria as they depend on the prey natural carrying capacity.

Some researchers have studied predator–prey systems with age structure by transforming these systems into non-densely defined abstract Cauchy problems. For example, Fu et al. (2015) considered an age-structured population model with two time delays and investigated some dynamic properties of the system by using the integral semigroup theory, including asymptotic stability and existence of Hopf bifurcation at the positive steady state. Yang and Wang (2020b) proposed an age-dependent predator–prey system with strong Allee effect, discussed the existence and uniqueness of a nonnegative steady state by transforming the system into a non-densely defined abstract Cauchy problem, and studied Hopf-Zero bifurcation by applying the center manifold theorem (Magal and Ruan 2009a) and the normal form theory (Liu et al. 2014; Chu et al. 2016). We refer to Li (1990), Zhang and Liu (2019, 2020, 2021), Yang (2019), Yang and Wang (2019, 2020a), Cai et al. (2020) and Yuan and Fu (2022) on further studies of predator–prey models with age structure and to Webb (1985) and Magal and Ruan (2008) for fundamental theories in classical age-structured models.

For biological population systems, the dynamical behaviors can be very complex due to many factors. Researchers have studied the nonlinear dynamics by examining the complex interactions among populations. The average rate of predator consumption of the prey, also known as the functional response, is an important indicator to describe such interactions. There are several types of functional response including Monod–Haldane (M–H) (Yang and Wang 2020b), Holling (Chen et al. 2010; Yang 2019), Beddington–DeAngelis (B–D) (Beddington 1975; DeAngelis et al. 1975; Tripathi et al. 2015), Crowley–Martin (C–M) (Wang and Song 2016), Leslie–Gower (L–G) (Yang and Zhang 2017; Wang et al. 2019; Singh and Malik 2021), etc. depending on different species of predators and prey. Note that different functional response functions can induce different dynamical behaviors and cause various bifurcations. Therefore, to better describe natural phenomena and biological relationships between populations, it is necessary to introduce different functional response into different population dynamical models. Tripathi et al. (2015) proposed a predator–prey model with B–D type functional response and prey refuge as follows:

1.2 $$\begin{aligned} \left\{ \begin{aligned}&\frac{\text{ d } X}{\text{ d } t}=rX\left( 1-\frac{X}{K}\right) -\frac{B(1-m)XY}{C+A_1(1-m)X+A_2Y},\\&\frac{\text{ d } Y}{\text{ d } t}=-DY+\frac{EB(1-m)XY}{C+A_1(1-m)X+A_2Y},\\ \end{aligned} \right. \end{aligned}$$

where *X*(*t*) and *Y*(*t*) denote the density of the prey and predators at time *t*, respectively; *D* and $m~(0\le m<1)$ stand for the natural mortality rate for predators and the number of prey refuge, respectively; *B* and *E* are the capture rate and conversion rate of predators, respectively; $A_1$ denotes the effect of handling time for predators; *C* and $A_2$ are the half saturation constant and the interference coefficient among predators, respectively. The biological significance of other parameters remain consistent with system (1.1). Tripathi et al. obtained different conditions that affect the persistence of the system and discussed local and global asymptotic stability of various equilibria. At the same time, the influence of predators’ interference degree $A_2$ on system stability was analyzed.

In system (1.2), the B–D functional response $\frac{B(1-m)X}{C+A_1(1-m)X+A_2Y}$ represents not only the interaction between predators and preys (the term $A_1X$) but also the mutual interference among predators (the term $A_2Y$). However, if the interference among predators is not considered ($A_2=0$), system (1.2) reduces into a predator–prey system with Holling-II type, which has been studied in Kar (2005). By comparing the results of Kar (2005) and Tripathi et al. (2015), we can see that different functional response functions lead to different dynamic behaviors within the same parameter range, which suggests two different biological characteristics. These results suggest that it is necessary to examine the effects of different functional response functions.

In addition, along with the evolution of time and aging of the population, the capture level of predators will decline gradually, which will lead to an increase in the prey population. Moreover, large carnivores themselves are valuable and will bring considerable economic benefits to human beings, so it is worth exploring the harvesting strategies for the prey population. Based on the model in Kar (2005), considering harvesting strategies for both predator and prey populations, a predator–prey model with Holling-II type and constant harvesting takes the following form:

1.3 $$\begin{aligned} \left\{ \begin{aligned}&\frac{\text{ d } X}{\text{ d } t}=rX\left( 1-\frac{X}{K}\right) -\frac{XY}{C+X}-H_1,\\&\frac{\text{ d } Y}{\text{ d } t}=-DY+\frac{XY}{C+X}-H_2,\\ \end{aligned} \right. \end{aligned}$$

where $H_1$ and $H_2$ are constant harvesting rates of the prey and predators, respectively. The biological significance of other parameters remain consistent with system (1.2). On one hand, if $H_1=0$, Xiao and Ruan (1999) carried out a bifurcation analysis of system (1.3), showed that codimension 2 bifurcations occur in a two-dimensional parameter region, and proved system (1.3) undergoes Bogdanov–Takens bifurcation under some conditions. On the other hand, Martin and Ruan (2001) studied the combined effects of the prey harvesting and time delay on the dynamics of the generalized Gause-type predator–prey models when $H_2=0$. It is shown that in these models the time delay can cause a stable equilibrium to become unstable, while the prey harvesting rate has a stabilizing effect on the equilibrium if it is under the critical harvesting level. After that, Xia et al. (2009) studied the effects of harvesting and time delay on predator–prey systems with Holling-II functional response. They considered two different types of harvesting; namely prey harvesting and predator harvesting. Their results indicated that in the model with prey harvesting there is no bifurcation at the positive equilibrium and time delay can induce oscillations of both species via Hopf bifurcation. While in the model with predator harvesting, multiple positive equilibria and degenerate equilibria can exist, and Bogdanov–Takens bifurcation can occur. These results indicate that harvesting on different populations (predators or prey) will induce different nonlinear dynamics and harvesting on predators will result in more complex dynamical behaviors. We refer to Yang and Zhang (2017), Liu et al. (2018), Singh and Malik (2021), Yang and Wang (2020a) and Meng and Li (2021) for further studies on delayed predator–prey models with harvesting.

Notice that newborn predators will be affected by resources and the surrounding environment; that is, the birth rate will be limited which is related to not only the population density of the surrounding prey but also themselves. Based on the above discussions, we propose to consider the following facts that are consistent with natural phenomena:

- Since the birth, aging, illness, and death of populations are all related to age in nature, it is practically significant to study predator–prey systems with age-structure. However, predator–prey systems with age-structure is more complex than the classical predator–prey system.
- Because the age of a predator has a significant impact on its capture level, and the number of prey population will gradually increase as the predator capture level decreases. Therefore, it is necessary to adopt harvesting strategies for the prey population, which not only brings certain economic benefits but also determines the stability of the system;
- It is more realistic to use the B–D functional response function to replace *h*(*R*(*t*), *P*(*t*)) and *g*(*R*(*t*), *P*(*t*)) with the general linear “mass action” type functional response in Cushing and Saleem (1982) (where the biological interpretations of *h*(*R*(*t*), *P*(*t*)) and *g*(*R*(*t*), *P*(*t*)) are given in system (1.1));
- It is assumed that both the maturation function $\beta (a)$ and the fertility function *f*(*a*) are age-related functions as populations cannot reproduce until they are mature enough.

Based on the above facts and motivated by Cushing and Saleem (1982), Tripathi et al. (2015), Xia et al. (2009), Kar (2005), we propose an age-structured predator–prey system with B–D functional response and constant harvesting, which combines a partial differential equation and an ordinary differential equation as follows:

1.4 $$\begin{aligned} \left\{ \begin{aligned}&\frac{\partial u(t,a)}{\partial t}+\frac{\partial u(t,a)}{\partial a}=-\mu u(t,a),\\&\frac{\text{ d } V(t)}{\text{ d } t}=rV(t)\left( 1-\frac{V(t)}{K}\right) -\frac{(1-m)V(t)\int _{0}^{+\infty }\beta (a)u(t,a)\text{ d }a}{\alpha +(1-m)V(t)+s\int _{0}^{+\infty }u(t,a)\text{ d }a}-M,\\&u(t,0)=\frac{\eta (1-m)V(t)\int _{0}^{+\infty }f(a)u(t,a)\text{ d }a}{\alpha +(1-m)V(t)+s\int _{0}^{+\infty }u(t,a)\text{ d }a},\\&u(0,a)=u_0(a)\in L_+^{1}((0,+\infty ),\mathbb {R}),\;\,V(0)=V_0\ge 0, \end{aligned} \right. \end{aligned}$$

where *V*(*t*) is the density of the prey population at time *t*, and *u*(*t*, *a*) is the density of the predator population at time *t* with age *a*; *r* is the intrinsic growth rate of the prey ($r=\Lambda -d$, where $\Lambda$ and *d* are the birth rate and death rate of the prey population, respectively); $\mu$ and $\eta$ are the death rate of predators and the conversion that predators intake to per capital prey, respectively; *M* is the constant harvesting rate of the prey population; $\beta (a)\in L_+^{\infty }((0,+\infty ),\mathbb {R})$ is the maturation function which describes the effect of age on fecundity; $f(a)\in L_+^{\infty }((0,+\infty ),\mathbb {R})$ is a fertility function related to predator age *a* with $f(a)=b\beta (a)$ and $b\ge 1$ is the birth modulus. The biological interpretations of other parameters are same as in system (1.2) and all parameters are positive constants. $\beta (a)$ and *f*(*a*) satisfy the following assumption:

### Assumption 1.1

Assume that

$$\begin{aligned} \beta (a)= \left\{ \begin{array}{ll} 0 &{}\quad \text {if}\;\,a\in (0,\tau ),\\ \beta ^* &{}\quad \text {if}\;\,a\ge \tau \\ \end{array} \right. \quad \textrm{and}\quad f(a)= \left\{ \begin{array}{ll} 0 &{}\quad \text {if}\;\, a\in (0,\tau ),\\ f^* &{}\quad \text {if}\;\, a\ge \tau ,\\ \end{array} \right. \end{aligned}$$

where $\tau >0$ is the maturation period of predators. Let $\int _{0}^{+\infty }\beta (a)e^{-\mu a}\text{ d }a=1$, which is called the net maturation rate. $\int _{0}^{+\infty }f(a)e^{-\mu a}\text{ d }a=b~ (1\le b<\infty )$ is the net reproduction rate, which gives the number of newborns that an female individual is expected to produce during her reproductive life, where $e^{-\mu a}$ is the survival probability and $\mu >0$. In order to obtain the smooth dependency of system (1.4) with respect to $\tau$, we first normalize $\tau$ in (1.4). Take the time-scaling $\hat{t}=\frac{t}{\tau }$, age-scaling $\hat{a}=\frac{a}{\tau }$, and the change of variables $\hat{V}(\hat{t})=V(\tau \hat{t})$, $\hat{u}(\hat{t},\hat{a})=\tau u(\tau \hat{t},\tau \hat{a})$. Then dropping the hat notation for convenience, we obtain the new system as follows:

1.5 $$\begin{aligned} \left\{ \begin{aligned}&\frac{\partial u(t,a)}{\partial t}+\frac{\partial u(t,a)}{\partial a}=-\tau \mu u(t,a),~~~a\ge 0,\\&\frac{\text{ d } V(t)}{\text{ d } t}=\tau \left[ rV(t)\Big (1-\frac{V(t)}{K}\Big )-\frac{(1-m)V(t)\int _{0}^{+\infty }\beta (a)u(t,a)\text{ d }a}{\alpha +(1-m)V(t)+s\int _{0}^{+\infty }u(t,a)\text{ d }a}-M\right] ,\\&u(t,0)=\tau \left[ \frac{\eta (1-m)V(t)\int _{0}^{+\infty }f(a)u(t,a)\text{ d }a}{\alpha +(1-m)V(t)+s\int _{0}^{+\infty }u(t,a)\text{ d }a}\right] ,\quad t>0,\\&u(0,a)=u_0(a)\in L_+^{1}((0,+\infty ),\mathbb {R}),\quad V(0)=V_0\ge 0, \end{aligned} \right. \end{aligned}$$

where the new maturation function $\beta (a)$ and new fertility function *f*(*a*) become

$$\begin{aligned} \beta (a)=\beta ^*1_{[1,+\infty ]}(a)= \left\{ \begin{array}{ll} 0 &{}\quad \text {if}\;\,0<a<1,\\ \beta ^* &{}\quad \text {if}\;\,a\ge 1 \end{array} \right. \quad \text{ with } \quad \beta ^*=\mu e^{\mu \tau } \end{aligned}$$

and

$$\begin{aligned} f(a)=f^*1_{[1,+\infty ]}(a)= \left\{ \begin{array}{ll} 0 &{}\quad \text {if}\;\,0<a<1,\\ f^* &{}\quad \text {if}\;\,a\ge 1 \end{array}\right. \quad \text{ with } \quad f^*=b\mu e^{\mu \tau }. \end{aligned}$$

The connection between the asymptotic behavior of system (1.4) and (1.5) is given in Remark 1.1.

### Remark 1.1

1. If $\tau >0$, the solutions of the initial value problem for system (1.4) are equivalent to solutions of system (1.5) under the change of variables. In fact, the main purpose of this paper is to study the existence of Hopf bifurcation of system (1.4) when $\tau >0$, which is obviously equivalent to the existence of Hopf bifurcation in system (1.5); i.e., whether the characteristic equation corresponding to the linearized system of (1.5) admits pure imaginary roots when $\tau >0$.
2. If $\tau =0$, all terms of system (1.5) are zero, then the system is meaningless. Therefore, we only consider system (1.5) when $\tau >0$. In fact, in the case of $\tau =0$, denote the total number of predators by $U(t)=\int _{0}^{+\infty }u(t,a)\text{ d }a$ and integrate the first equation of system (1.4). Then system (1.4) can be transformed into an ODE system as follows:
  $$\begin{aligned} \left\{ \begin{aligned}&\frac{\text{ d } V(t)}{\text{ d } t}=rV(t)\left( 1-\frac{V(t)}{K}\right) -\frac{\mu (1-m)V(t)U(t)}{\alpha +(1-m)V(t)+sU(t)}-M,\\&\frac{\text{ d } U(t)}{\text{ d } t}=\frac{b\mu \eta (1-m)V(t)U(t)}{\alpha +(1-m)V(t)+sU(t)}-\mu U(t),\\&V(0)=V_0\ge 0,\quad U(0)=U_0\ge 0, \end{aligned} \right. \end{aligned}$$
  in which $\beta (a)\equiv \beta ^*=\mu$ and $f(a)\equiv f^*=b\mu$ at $\tau =0$. Hence, when $\tau =0$, to consider the existence of Cauchy problem of (1.4) and the existence and stability of positive steady states, it is sufficient to directly consider that for the above ODE system.

The organization of this paper is as follows. In Sect. 2, we transform the original system into a non-densely defined abstract Cauchy problem. In Sect. 3, the existence and uniqueness of a positive equilibrium are discussed. In Sect. 4, the characteristic equation of the linear system, the stability of the positive equilibrium and the existence of Hopf bifurcation are studied. In Sect. 5, the direction of Hopf bifurcation and stability of the bifurcating periodic solutions are considered. In Sects. 6 and 7, some numerical simulations and a brief discussion are given, respectively.

## Non-densely defined abstract Cauchy problem

Similar to Liu et al. (2011, Subsection 5.3), we transform system (1.5) into a non-densely defined abstract Cauchy problem to make use of the integrated semigroup theory. Let $V(t)=\int _{0}^{+\infty }v(t,a)\text{ d }a$ in system (1.5), then we have

2.1 $$\begin{aligned} \left\{ \begin{aligned}&\frac{\partial v(t,a)}{\partial t}+\frac{\partial v(t,a)}{\partial a}=-\tau d v(t,a),\\&v(t,0)={{\tau G(u(t,\cdot ),v(t,\cdot ))}},\\&v(0,a)=v_0(a)\in L_+^1((0,+\infty ),\mathbb {R}), \end{aligned} \right. \end{aligned}$$

where

$$\begin{aligned} \begin{aligned} {{\tau G(u(t,\cdot ),v(t,\cdot ))}}&=\Lambda \int _{0}^{+\infty }v(t,a)\text{ d }a-\frac{r}{K}\left( \int _{0}^{+\infty }v(t,a)\text{ d }a\right) ^2\\&\quad -\frac{(1-m)\int _{0}^{+\infty }v(t,a)\text{ d }a\int _{0}^{+\infty }\beta (a)u(t,a)\text{ d }a}{\alpha +(1-m)\int _{0}^{+\infty }v(t,a)\text{ d }a+s\int _{0}^{+\infty }u(t,a)\text{ d }a}-M \end{aligned} \end{aligned}$$

and $r=\Lambda -d$ ($\Lambda$ and *d* are the birth rate and death rate of the prey population, respectively). Let $$w(t,a)=\left( \begin{array}{c} u(t,a)\\ v(t,a)\\ \end{array} \right) $$. We obtain the equivalent system of system (1.5):

2.2 $$\begin{aligned} \left\{ \begin{aligned}&\frac{\partial w(t,a)}{\partial t}+\frac{\partial w(t,a)}{\partial a}=-\tau Qw(t,a),\\&w(t,0)={{\tau B(w(t,\cdot ))}},\\&w(0,a)=\left( \begin{array}{c} u_0(a)\\ v_0(a)\\ \end{array} \right) \in L_+^1((0,+\infty ),\mathbb {R}^2), \end{aligned} \right. \end{aligned}$$

where

$$\begin{aligned} Q&=\left( \begin{array}{cc} \mu &{}0\\ 0&{}d\\ \end{array} \right) \quad \text{ and }\quad \\ {{B(w(t,\cdot ))}}&=\left( \begin{array}{c} \displaystyle \frac{\eta (1-m)\int _{0}^{+\infty }v(t,a)\textrm{d}a\int _{0}^{+\infty }f(a)u(t,a)\textrm{d}a}{\alpha +(1-m)\int _{0}^{+\infty }v(t,a)\textrm{d}a+s\int _{0}^{+\infty }u(t,a)\textrm{d}a}\\ {{G(u(t,\cdot ),v(t,\cdot ))}}\\ \end{array} \right) . \end{aligned}$$

Clearly, if $$\left( \begin{array}{c} {{u(t,\cdot )}}\\ {{v(t,\cdot )}}\\ \end{array} \right) $$ is the solution of system (2.2) with the initial value $$\left( \begin{array}{c} {{u_0}}\\ {{v_0}}\\ \end{array} \right) \in L_+^1((0,+\infty ),\mathbb {R}^2)$$, then $$\left( \begin{array}{c} {{u(t,\cdot )}}\\ \int _{0}^{+\infty }v(t,\cdot )\text{ d }a\\ \end{array} \right) =\left( \begin{array}{c} {{u(t,\cdot )}}\\ V(t)\\ \end{array} \right) $$ is the solution of system (1.5) with the initial value $$\left( \begin{array}{c} {{u_0}}\\ \int _{0}^{+\infty }v_0(\cdot )\textrm{d}a\\ \end{array} \right) =\left( \begin{array}{c} {{u_0}}\\ V_0\\ \end{array} \right) $$. Thus in order to investigate the existence of solutions of system (1.5), we need only to consider the existence of solutions of the equivalent system (2.2).

Following the results developed in Thieme (1990) and Magal (2001), we consider the Banach space $X:=\mathbb {R}^2\times L^1((0,+\infty ),\mathbb {R}^2)$ with the norm

$$\begin{aligned} \left\| \left( \begin{array}{c} \zeta \\ \varphi \\ \end{array} \right) \right\| =\Vert \zeta \Vert _{\mathbb {R}^2}+\Vert \varphi \Vert _{L^1((0,+\infty ),\mathbb {R}^2)},\quad \forall \left( \begin{array}{c} \zeta \\ \varphi \\ \end{array} \right) \in X. \end{aligned}$$

Define the linear operator ${{A_{\tau }:D(A_{\tau })\subset X\rightarrow X}}$ by

2.3 $$\begin{aligned} {{A_{\tau }}}\left( \begin{array}{c} 0_{\mathbb {R}^2}\\ \varphi \\ \end{array} \right) =\left( \begin{array}{c} -\varphi (0)\\ -\varphi ^{\prime }-\tau Q\varphi \\ \end{array} \right) \end{aligned}$$

with $D(A_{\tau })=\{0_{\mathbb {R}^2}\}\times W^{1,1}\left( (0,+\infty ),\mathbb {R}^2\right) \subset X,$ and the nonlinear operator $F:\overline{D(A_{\tau })}\rightarrow X$ by

2.4 $$\begin{aligned} F\left( \left( \begin{array}{c} 0_{\mathbb {R}^2}\\ \varphi \\ \end{array} \right) \right) =\left( \begin{array}{c} B(\varphi )\\ 0_{L^1((0,+\infty ),\mathbb {R}^2)}\\ \end{array} \right) , \end{aligned}$$

where $W^{1,1}((0,+\infty ),\mathbb {R}^2)=\{\varphi \in L^1((0,+\infty ),\mathbb {R}^2):\varphi ^{\prime }\in L^1((0,+\infty ),\mathbb {R}^2)\}$. Since $Z:=\overline{D(A_{\tau })}=\{0_{\mathbb {R}^2}\}\times L^{1}((0,+\infty ),\mathbb {R}^2)\ne X$, the linear operator $A_{\tau }$ is non-densely defined.

Let $$p(t)=\left( \begin{array}{{c}} 0_{\mathbb {R}^2}\\ {{w(t,\cdot )}}\\ \end{array} \right) $$ and identify *w*(*t*) with ${{w(t,\cdot )}}$, then system (2.2) can be rewritten as the following non-densely defined Cauchy problem:

2.5 $$\begin{aligned} \left\{ \begin{aligned}&\frac{\text{ d } p(t)}{\text{ d } t}=A_{\tau }p(t)+\tau F(p(t)),~t\ge 0,\\&p(0)=\left( \begin{array}{{c}} 0_{\mathbb {R}^2}\\ w_0\\ \end{array} \right) \in \overline{D(A_{\tau })}. \end{aligned} \right. \end{aligned}$$

The global existence, uniqueness and positive of solutions of system (2.5) follow from the results in Magal and Ruan (2009b), Magal and Ruan (2018) and Magal (2001). Furthermore, since $A_{\tau }$ is a Hille–Yosida operator, it generates a non-degenerated integrated semigroup $\{S_{A_{\tau }}(t)\}_{t\ge 0}$ on *X*. Define the linear operator $A_{0}$ with $$D(A_{0})=\left\{ \left( \begin{array}{{c}} 0_{\mathbb {R}^{2}}\\ \varphi \\ \end{array} \right) \in D(A_{\tau }):A_{\tau }\left( \begin{array}{{c}} 0_{\mathbb {R}^{2}}\\ \varphi \\ \end{array} \right) \in Z\right\} $$ and $$A_{0}\left( \begin{array}{{c}} 0_{\mathbb {R}^{2}}\\ \varphi \\ \end{array} \right) =A_{\tau }\left( \begin{array}{{c}} 0_{\mathbb {R}^{2}}\\ \varphi \\ \end{array} \right) , $$ then $A_{0}$ is the part of $A_{\tau }$ in $\overline{D(A_{\tau })}$ and $A_{0}$ generate a $C_0$-semigroups $\{T_{A_{0}}(t)\}_{t\ge 0}$ on *Z*. Let $X_{+}:=\mathbb {R}_{+}^{2}\times L_+^{1}((0,+\infty ),\mathbb {R}_{+}^2),$ $Z_{+}=Z\cap X_{+}:=\{0_{\mathbb {R}_{+}^{2}}\}\times L_+^{1}((0,+\infty ),\mathbb {R}_{+}^2)$, then we have the following result.

### Theorem 2.1

(Existence) There exists an unique continuous semiflow $\{\mathcal {U}(t)\}_{t\ge 0}$ on $Z_{+}$ such that for any $p\in Z_{+},~t\rightarrow \mathcal {U}(t)p$ is the unique integrated solution of the non-densely defined abstract Cauchy problem

$$\begin{aligned} \left\{ \begin{aligned}&\frac{\textrm{d} \mathcal {U}(t)p}{\textrm{d} t}=A_{\tau }\mathcal {U}(t)p+\tau F(\mathcal {U}(t)p),\quad t\ge 0,\\&\mathcal {U}(0)p=p. \end{aligned} \right. \end{aligned}$$

In other words, for each $t\ge 0$, the map $t\rightarrow \mathcal {U}(t)p$ satisfies $\int _{0}^{t}\mathcal {U}(l)p\textrm{d}l\in \overline{D(A_{\tau })}$,

$$\begin{aligned} \mathcal {U}(t)p=p+A_{\tau }\int _{0}^{t}\mathcal {U}(l)p\textrm{d}l+\tau \int _{0}^{t}F(\mathcal {U}(l)p)\textrm{d}l,\quad t\ge 0, \end{aligned}$$

which is equivalent to $\mathcal {U}(t)p=T_{A_{0}}(t)p+\frac{\textrm{d}}{\textrm{d}t}\Big (S_{A_{\tau }}*\tau F(\mathcal {U}(a)p)\Big )(t),~t\ge 0.$ Moreover, $\{\mathcal {U}(t)\}_{t\ge 0}$ is a continuous semiflow on $Z_{+}$; that is,

(i) $$\mathcal {U}(t)\mathcal {U}(l)=\mathcal {U}(t+l),~\forall ~ t,l\ge 0,$$
(ii) $\mathcal {U}(0)=I$, where *I* is an identity operator and the map $(t,p)\rightarrow \mathcal {U}(t)p$ is continuous from $[0,+\infty )\times Z_{+}$ into $Z_{+}$.

## Existence and uniqueness of equilibria

Let $$\overline{p}(a)=\left( \begin{array}{{c}} 0_{\mathbb {R}^2}\\ \overline{w}(a)\\ \end{array} \right) \in D(A_{\tau })$$ be a steady state of system (2.5). Then we have

$$\begin{aligned} A_{\tau }\left( \begin{array}{{c}} 0_{\mathbb {R}^2}\\ \overline{w}(a)\\ \end{array} \right) +\tau F\left( \left( \begin{array}{{c}} 0_{\mathbb {R}^2}\\ \overline{w}(a)\\ \end{array} \right) \right) =0; \end{aligned}$$

that is,

3.1 $$\begin{aligned} \left\{ \begin{aligned}&-\overline{w}(0)+{{\tau B(\overline{w})}}=0,\\&-\overline{w}^{\prime }(a)-{{\tau Q(\overline{w})}}=0. \end{aligned} \right. \end{aligned}$$

From (3.1), we can get

3.2 $$\begin{aligned} \overline{w}(a)=\left( \begin{array}{{c}} \overline{u}(a)\\ \overline{v}(a)\\ \end{array} \right) =\left( \begin{array}{{c}} \displaystyle \tau \frac{\eta (1-m)\overline{V}\int _{0}^{+\infty }f(a)\overline{u}(a)\textrm{d}a}{\alpha +(1-m)\overline{V}+s\int _{0}^{+\infty }\overline{u}(a)\textrm{d}a}e^{-\tau \mu a}\\ {{\tau \left[ \Lambda \overline{V}-\frac{r}{K}\overline{V}^2 -\frac{(1-m)\overline{V}\int _{0}^{+\infty }\beta (a)\overline{u}(a)\textrm{d}a}{\alpha +(1-m)\overline{V}+s\int _{0}^{+\infty }\overline{u}(a)\textrm{d}a}-M\right] e^{-\tau d a}}}\\ \end{array} \right) , \end{aligned}$$

where $\overline{V}=\int _{0}^{+\infty }\overline{v}(a)\text{ d }a$. Integrating equation (3.2) from 0 to $+\infty$, we have

3.3 $$\begin{aligned} \mu \int _{0}^{+\infty }\overline{u}(a)\textrm{d}a&=\frac{\eta (1-m)\overline{V}\int _{0}^{+\infty }f(a)\overline{u}(a)\textrm{d}a}{\alpha +(1-m)\overline{V}+s\int _{0}^{+\infty }\overline{u}(a)\textrm{d}a}, \end{aligned}$$

3.4 $$\begin{aligned} \int _{0}^{+\infty }f(a)\overline{u}(a)\textrm{d}a&=\tau \frac{\eta (1-m)\overline{V}\int _{0}^{+\infty }f(a)\overline{u}(a)\textrm{d}a}{\alpha +(1-m)\overline{V}+s\int _{0}^{+\infty }\overline{u}(a)\textrm{d}a}\int _{0}^{+\infty }f(a)e^{-\tau \mu a}\textrm{d}a\nonumber \\&=b\mu \int _{0}^{+\infty }\overline{u}(a)\textrm{d}a \end{aligned}$$

and

3.5 $$\begin{aligned} {{r\overline{{V}}-\frac{r}{K}\overline{V}^2 -\frac{(1-m)\overline{V}\int _{0}^{+\infty }\beta (a)\overline{u}(a)\textrm{d}a}{\alpha +(1-m)\overline{V}+s\int _{0}^{+\infty }\overline{u}(a)\textrm{d}a}-M=0.}} \end{aligned}$$

By substituting (3.3) into (3.4), we obtain that $\int _{0}^{+\infty }\overline{u}(a)\textrm{d}a=\frac{P\overline{V}-\alpha }{s}$, where $P=(b\eta -1)(1-m)$ and $\overline{V}$ is a root of the following quadratic equation:

3.6 $$\begin{aligned} \theta _1\overline{V}^2+\theta _2\overline{V}+\theta _3=0 \end{aligned}$$

with

3.7 $$\begin{aligned} \theta _1=s\eta b r,\; \theta _2=K(\mu P-s b\eta r),\; \theta _3=K(s b\eta M-\mu \alpha ) \end{aligned}$$

and

3.8 $$\begin{aligned} \Delta =\theta _2^2-4\theta _1\theta _3=[K(\mu P-s b\eta r)]^2-4s\eta b rK(s b\eta M-\mu \alpha ). \end{aligned}$$

Clearly, $\int _{0}^{+\infty }\overline{u}(a)\textrm{d}a>0$ if and only if $\overline{V}>0$. Now, we discuss the following three cases about the roots of (3.6).

**(I)**: $\Delta =0$. This means that $\theta _2^2=4\theta _1\theta _3$. Then we have the following three subcases:
  (1): If $\theta _2>0$, i.e., $\mu P>s b\eta r$, then Eq. (3.6) has two equal negative real roots:
    $$\begin{aligned} \overline{V}_{1111}=\overline{V}_{1112}=\frac{-K(\mu P-s b\eta r)}{2sb\eta r}<0; \end{aligned}$$
  (2): If $\theta _2<0$, i.e., $\mu P<s b\eta r$, then Eq. (3.6) has two equal positive real roots:
    $$\begin{aligned} \overline{V}_{1211}=\overline{V}_{1212}=\frac{-K(\mu P-s b\eta r)}{2sb\eta r}>0; \end{aligned}$$
  (3): If $\theta _2=0$, i.e., $\mu P=s b\eta r$, then Eq. (3.6) has two equal zero real roots:
    $$\begin{aligned} \overline{V}_{1311}=\overline{V}_{1312}=0. \end{aligned}$$
**(II)**: $\Delta <0$; that is, $\theta _2^2<4\theta _1\theta _3$. Then Eq. (3.6) has no real roots.
**(III)**: $\Delta >0$; namely, $\theta _2^2>4\theta _1\theta _3$. In this case we have the following seven subcases:
  (1): If $\theta _2>0$ and $\theta _3>0$, i.e., $\mu P>s b\eta r$ and $\frac{\mu \alpha }{sb\eta }<M<\frac{[K(\mu P-s b\eta r)]^2+4sb\eta rK\mu \alpha }{4\,s^2b^2\eta ^2rK}$, then $\Delta =\theta _2^2-4\theta _1\theta _3<\theta _2^2$, $0<\sqrt{\Delta }<\theta _2$ and hence Eq. (3.6) has two negative real roots:
    $$\begin{aligned} \overline{V}_{3111}&=\frac{-K(\mu P-s b\eta r)+\sqrt{\Delta }}{2sb\eta r}<0\quad \textrm{and}\\ \overline{V}_{3112}&=\frac{-K(\mu P-s b\eta r)-\sqrt{\Delta }}{2sb\eta r}<0; \end{aligned}$$
  (2): If $\theta _2>0$ and $\theta _3<0$, i.e., $\mu P>s b\eta r$ and $0\le M<\frac{\mu \alpha }{s\eta b}$, then $\Delta =\theta _2^2-4\theta _1\theta _3>0$ and $\sqrt{\Delta }>\theta _2$. Hence Eq. (3.6) has two real roots:
    $$\begin{aligned} \overline{V}_{3211}&=\frac{-K(\mu P-s b\eta r)+\sqrt{\Delta }}{2sb\eta r}>0\quad \textrm{and}\\ \overline{V}_{3212}&=\frac{-K(\mu P-s b\eta r)-\sqrt{\Delta }}{2sb\eta r}<0; \end{aligned}$$
  (3): If $\theta _2>0$ and $\theta _3=0$, i.e., $\mu P>s b\eta r$ and $M=\frac{\mu \alpha }{sb\eta }$, then $\Delta =\theta _2^2-4\theta _1\theta _3=\theta _2^2>0$ and $\sqrt{\Delta }=\theta _2$. Equation (3.6) has two real roots:
    $$\begin{aligned} \overline{V}_{3311}&=\frac{-K(\mu P-s b\eta r)+\sqrt{\Delta }}{2sb\eta r}=0\quad \textrm{and}\\ \overline{V}_{3312}&=\frac{-\theta _2-\sqrt{\Delta }}{2\theta _1}=\frac{-K(\mu P-s b\eta r)}{sb\eta r}<0. \end{aligned}$$
  (4): If $\theta _2<0$ and $\theta _3<0$, i.e., $\mu P<s b\eta r$ and $0\le M<\frac{\mu \alpha }{s\eta b}$, then $\Delta =\theta _2^2-4\theta _1\theta _3>\theta _2^2$, $\sqrt{\Delta }>|\theta _2|$ and Eq. (3.6) has two real roots:
    $$\begin{aligned} \overline{V}_{3411}&=\frac{-K(\mu P-s b\eta r)+\sqrt{\Delta }}{2sb\eta r}>0\quad \textrm{and}\\ \overline{V}_{3412}&=\frac{-K(\mu P-s b\eta r)-\sqrt{\Delta }}{2sb\eta r}<0; \end{aligned}$$
  (5): If $\theta _2<0$ and $\theta _3>0$, i.e., $\mu P<s b\eta r$ and $\frac{\mu \alpha }{sb\eta }<M<\frac{[K(\mu P-s b\eta r)]^2+4sb\eta rK\mu \alpha }{4\,s^2b^2\eta ^2rK}$, then $\Delta =\theta _2^2-4\theta _1\theta _3<\theta _2^2$, $0<\sqrt{\Delta }<|\theta _2|$ and Eq. (3.6) has two positive real roots:
    $$\begin{aligned} \overline{V}_{3511}&=\frac{-K(\mu P-s b\eta r)+\sqrt{\Delta }}{2sb\eta r}>0\quad \textrm{and}\\ \overline{V}_{3512}&=\frac{-K(\mu P-s b\eta r)-\sqrt{\Delta }}{2sb\eta r}>0; \end{aligned}$$
  (6): If $\theta _2<0$ and $\theta _3=0$, i.e., $\mu P<s b\eta r$ and $M=\frac{\mu \alpha }{sb\eta }$, then $\Delta =\theta _2^2-4\theta _1\theta _3=\theta _2^2>0$, $\sqrt{\Delta }=|\theta _2|$ and Eq. (3.6) has two real roots:
    $$\begin{aligned} \overline{V}_{3611}&=\frac{-\theta _2+\sqrt{\Delta }}{2\theta _1}=\frac{-K(\mu P-s b\eta r)}{sb\eta r}>0\quad \textrm{and}\\ \overline{V}_{3612}&=\frac{-K(\mu P-s b\eta r)-\sqrt{\Delta }}{2sb\eta r}=0. \end{aligned}$$
  (7): If $\theta _2=0$ and $\theta _3<0$, i.e., $\mu P=s b\eta r$ and $0\le M<\frac{\mu \alpha }{sb\eta }$, then Eq. (3.6) has two real roots:
    $$\begin{aligned} \overline{V}_{3711}=\frac{\sqrt{\Delta }}{2sb\eta r}>0\quad \textrm{and}\quad \overline{V}_{3712}=\frac{-\sqrt{\Delta }}{2sb\eta r}<0. \end{aligned}$$

Because Eq. (3.6) has multiple roots and no real roots in the cases that $\Delta =0$ and $\Delta <0$, respectively, in the present work we only consider the case that $\Delta >0$. Note that when $\Delta >0$, Eq. (3.6) always has positive real roots which are simple. Because of $\overline{U}=\int _{0}^{+\infty }\overline{u}(a)\textrm{d}a=\frac{P\overline{V}-\alpha }{s}$, in order to ensure $\overline{U}>0$, we list the following two conditions:

1. $$P\sqrt{\Delta }-PK(\mu P-s b\eta r)>2sb\eta r\alpha $$;
2. $$-PK(\mu P-s b\eta r)-P\sqrt{\Delta }>2sb\eta r\alpha $$.

Then we have the following lemma for the existence of positive equilibria of system (1.5).

### Lemma 3.1

(i) If $\Delta >0$, $\theta _2>0$, $\theta _3<0$, $b\eta >1$ and $\mathrm {(H1)}$ holds, then system (1.5) has a coexistence equilibrium $$\overline{p}_{21}= \left( \begin{array}{{c}} \overline{u}_{3211}(a)\\ \overline{V}_{3211}\\ \end{array} \right) $$;
(ii) If $\Delta >0$, $\theta _2<0$, $\theta _3<0$, $b\eta >1$ and $\mathrm {(H1)}$ holds, then system (1.5) has a coexistence equilibrium $$\overline{p}_{22}= \left( \begin{array}{{c}} \overline{u}_{3411}(a)\\ \overline{V}_{3411}\\ \end{array} \right) $$, where $\overline{u}_{3411}(a)=\tau \mu \frac{-PK(\mu P-s b\eta r)+P\sqrt{\Delta }-2sb\eta r\alpha }{2\,s^2 b\eta r}e^{-\tau \mu a}$;
(iii) If $\Delta >0$, $\theta _2<0$, $\theta _3>0$, $b\eta >1$ and $\mathrm {(H1)}$ holds, then system (1.5) has a coexistence equilibrium $$\overline{p}_{31}= \left( \begin{array}{{c}} \overline{u}_{3511}(a)\\ \overline{V}_{3511}\\ \end{array} \right) $$. Furthermore, if $\mathrm {(H2)}$ also holds, then system (1.5) admits another coexistence equilibrium $$\overline{p}_{32}= \left( \begin{array}{{c}} \overline{u}_{3512}(a)\\ \overline{V}_{3512}\\ \end{array} \right) $$;
(iv) If $\Delta >0$, $\theta _2<0$, $\theta _3=0$, $b\eta >1$ and $\mathrm {(H1)}$ holds, then system (1.5) has a coexistence equilibrium $$\overline{p}_{4}= \left( \begin{array}{{c}} \overline{u}_{3611}(a)\\ \overline{V}_{3611}\\ \end{array} \right) $$;
(v) If $\Delta >0$, $\theta _2=0$, $b\eta >1$ and $\mathrm {(H1)}$ holds, then system (1.5) has a coexistence equilibrium $$\overline{p}_{5}= \left( \begin{array}{{c}} \overline{u}_{3711}(a)\\ \overline{V}_{3711}\\ \end{array} \right) $$.

In the rest of this paper, we focus on case (ii) in Lemma 3.1. Clearly, if $0\le M<\frac{\mu \alpha }{sb\eta }$, then there must be $\Delta >0$. Therefore, in the following we always make the following assumption.

### Assumption 3.1

$$\mu P<s b\eta r$$, $$0\le M<\frac{\mu \alpha }{sb\eta }$$, $$b\eta >1$$ and $$\mathrm {(H1)}$$ holds.

When Assumption 3.1 holds, it follows from case (ii) in Lemma 3.1 that system (1.5) admits a unique positive equilibrium of age distribution:

3.9 $$\begin{aligned} \overline{p}=\left( \begin{array}{{c}} \overline{u}_{+}(a)\\ \overline{V}\\ \end{array} \right) = \left( \begin{array}{{c}} \tau \mu \frac{-PK(\mu P-s b\eta r)+P\sqrt{\Delta }-2sb\eta r\alpha }{2s^2 b\eta r}e^{-\tau \mu a}\\ \frac{-K(\mu P-s b\eta r)+\sqrt{\Delta }}{2sb\eta r}\\ \end{array} \right) , \end{aligned}$$

where $\overline{u}_+(a):=\overline{u}_{3411}(a),~\overline{V}:=\overline{V}_{3411}$. According to Theorem 4.1 in next section, the unique positive equilibrium is linearly stable when the delay $\tau =0$. In fact, since there is $\Delta >0$ when $0\le M<\frac{\mu \alpha }{sb\eta }$ (in other word, $\theta _3<0$), it follows from Lemma 3.1(i)(ii)(v) that the $\overline{V}$-component always admits a unique positive root when $0\le M<\frac{\mu \alpha }{sb\eta }$. This indicates that the prey population will be persistent if overfishing is not carried out. Otherwise the prey population may tend to extinction. Meanwhile, the conditions $b\eta >1$ and $\mathrm {(H1)}$ ensure that the $\overline{U}$-component is also positive due to the fact that $\overline{U}=\int _{0}^{+\infty }\overline{u}(a)\textrm{d}a=\frac{P\overline{V}-\alpha }{s}$, where $P=(b\eta -1)(1-m)$. Recall that *b* and $\eta$ are the birth modulus and the conversion rate of predators, respectively, and then $b\eta$ is the net conversion rate of newborn predators. Then the conditions $b\eta >1$ and $\mathrm {(H1)}$ imply that if the net conversion rate $b\eta$ of newborn predators is sufficiently large and the death rate $\mu$ and the half saturation constant $\alpha$ of predators are small enough, then the predator population will also survive. Nevertheless, in this work we only focus on case (ii) in Lemma 3.1 (that is, Assumption 3.1), and the other cases can be studied similarly. Thus, we have the following proposition.

### Proposition 3.1

(i) Suppose that Assumption 1.1 holds, then system (2.5) always has the boundary equilibria
  $$\begin{aligned} \overline{p}_{01}(a)=\left( \begin{array}{{c}} 0_{\mathbb {R}^{2}}\\ \left( \begin{array}{{c}} 0_{L^{1}((0,+\infty ),\mathbb {R})}\\ \tau d\frac{K r+\sqrt{\aleph }}{2r}e^{-\tau d a}\\ \end{array} \right) \end{array} \right) \quad \textrm{and}\quad \overline{p}_{02}(a)=\left( \begin{array}{{c}} 0_{\mathbb {R}^{2}}\\ \left( \begin{array}{{c}} 0_{L^{1}((0,+\infty ),\mathbb {R})}\\ \tau d\frac{K r-\sqrt{\aleph }}{2r}e^{-\tau d a}\\ \end{array} \right) \end{array} \right) \end{aligned}$$
  if $Kr-4M>0$; that is, $0\le M<\frac{Kr}{4}$, where $\aleph =Kr(Kr-4M)$.
(ii) Suppose that Assumptions 1.1 and 3.1 hold, then system (2.5) has a unique coexistence equilibrium
  $$\begin{aligned} \overline{p}_{+}(a)=\left( \begin{array}{{c}} 0_{\mathbb {R}^{2}}\\ {{\overline{w}_{\tau }(a)}}\\ \end{array} \right) =\left( \begin{array}{{c}} 0_{\mathbb {R}^{2}}\\ \left( \begin{array}{{c}} \tau \mu \frac{-PK(\mu P-s b\eta r)+P\sqrt{\Delta }-2s\eta br\alpha }{2s^2 b\eta r}e^{-\tau \mu a}\\ \tau d\frac{-K(\mu P-s b\eta r)+\sqrt{\Delta }}{2sb\eta r}e^{-\tau d a}\\ \end{array} \right) \end{array} \right) . \end{aligned}$$

## Existence of Hopf bifurcation

In this section, under Assumptions 1.1 and 3.1, we consider the local stability of the unique coexistence equilibrium $\overline{p}$ when $\tau =0$. Then we study the existence of Hopf bifurcation by using the Hopf bifurcation theory (Liu et al. 2011) to the Cauchy problem (2.5) and regarding the maturation period $\tau$ as a bifurcation parameter.

### Linearized system

In this subsection, we consider the linearized system of non-densely defined Cauchy problem (2.5) around the positive equilibrium $\overline{p}_{+}$. By a change of variable $\tilde{p}(t)=p(t)-\overline{p}_{+}$, system (2.5) becomes the following system:

4.1 $$\begin{aligned} \left\{ \begin{aligned}&\frac{\text{ d } \tilde{p}(t)}{\text{ d } t}=A_{\tau }\tilde{p}(t)+\tau F(\tilde{p}(t)+\overline{p}_{+})-\tau F(\overline{p}_{+}),\quad t\ge 0,\\&\tilde{p}(0)=\left( \begin{array}{{c}} 0_{\mathbb {R}^{2}}\\ w_0-{{\overline{w}_{\tau }}}\\ \end{array} \right) =\tilde{p}_0\in \overline{D(A_{\tau })}. \end{aligned} \right. \end{aligned}$$

Then the linearized system (4.1) is given by

4.2 $$\begin{aligned} \frac{\text{ d } \tilde{p}(t)}{\text{ d } t}=A_{\tau }\tilde{p}(t)+\tau DF(\overline{p}_{+})\tilde{p}(t)\quad \text{ for }\;t\ge 0,\;\tilde{p}(0)=\tilde{p}_0\in {{\overline{D(A_{\tau })}}}, \end{aligned}$$

where $$\tau DF(\overline{p}_{+})\left( \begin{array}{{c}} 0_{\mathbb {R}^{2}}\\ \varphi \\ \end{array} \right) =\left( \begin{array}{{c}} {{\tau DB(\overline{w}_{\tau })(\varphi )}}\\ 0_{L_+^{1}((0,+\infty ),\mathbb {R}^2)}\\ \end{array} \right) ,\;\forall \left( \begin{array}{{c}} 0_{\mathbb {R}^{2}}\\ \varphi \\ \end{array} \right) \in D(A_{\tau }) $$ with

4.3 $$\begin{aligned}&{{DB(\overline{w}_{\tau })(\varphi )}}\nonumber \\&\quad =\left( \begin{array}{ll} \displaystyle -\frac{s\eta (1-m)\overline{V}\int _{0}^{+\infty }f(a)\overline{u}(a)\textrm{d}a}{\left[ \alpha +(1-m)\overline{V}+s\int _{0}^{+\infty }\overline{u}(a)\textrm{d}a\right] ^2}&{} \frac{\eta (1-m)\int _{0}^{+\infty }f(a)\overline{u}(a)\textrm{d}a\left[ \alpha +s\int _{0}^{+\infty }\overline{u}(a)\textrm{d}a\right] }{\left[ \alpha +(1-m)\overline{V}+s\int _{0}^{+\infty }\overline{u}(a)\textrm{d}a\right] ^2}\\ \frac{s(1-m)\overline{V}\int _{0}^{+\infty }\beta (a)\overline{u}(a)\textrm{d}a}{\left[ \alpha +(1-m)\overline{V}+s\int _{0}^{+\infty }\overline{u}(a)\textrm{d}a\right] ^2}&{} \Lambda -\frac{2r}{K}\overline{V}-\frac{(1-m)\int _{0}^{+\infty }\beta (a)\overline{u}(a)\textrm{d}a \left[ \alpha +s\int _{0}^{+\infty }\overline{u}(a)\textrm{d}a\right] }{\left[ \alpha +(1-m)\overline{V}+s\int _{0}^{+\infty }\overline{u}(a)\textrm{d}a\right] ^2}\\ \end{array} \right) \nonumber \\&\qquad \times \int _{0}^{+\infty }\varphi (a)\text{ d }a+\left( \begin{array}{ll} \frac{\eta (1-m)\overline{V}}{\alpha +(1-m)\overline{V}+s\int _{0}^{+\infty }\overline{u}(a)\textrm{d}a}&{}0\\ 0&{}0\\ \end{array} \right) \times \int _{0}^{+\infty }f(a)\varphi (a)\text{ d }a\nonumber \\&\qquad +\left( \begin{array}{ll} 0&{}0\\ -\frac{(1-m)\overline{V}}{\alpha +(1-m)\overline{V}+s\int _{0}^{+\infty }\overline{u}(a)\textrm{d}a}&{}0\\ \end{array} \right) \times \int _{0}^{+\infty }\beta (a)\varphi (a)\text{ d }a. \end{aligned}$$

Therefore, system (4.1) can be rewritten as

4.4 $$\begin{aligned} \frac{\text{ d } \tilde{p}(t)}{\text{ d } t}=\tilde{A}\tilde{p}(t)+\tilde{F}(\tilde{p}(t))\quad \text{ for }\;t\ge 0,\;{{\tilde{p}(0)=\tilde{p}_0\in \overline{D(A_\tau )},}} \end{aligned}$$

where $\tilde{A}:=A_{\tau }+\tau DF(\overline{p}_{+})$ is a linear operator, and $\tilde{F}(\tilde{p}(t))=\tau F(\tilde{p}(t)+\overline{p}_{+})-\tau F(\overline{p}_{+})-\tau DF(\overline{p}_{+})\tilde{p}(t)$ satisfies $\tilde{F}(0)=0$ and $D\tilde{F}(0)=0$.

### Characteristic equation

Firstly, we consider the characteristic equation of system (2.5) at the positive equilibrium $\overline{p}_{+}$. Let

$$\begin{aligned} \vartheta :=\text{ min }\{\mu ,d\}>0\quad \text{ and }\quad \Omega :=\{\lambda \in \mathbb {C}:\text{ Re }(\lambda )>-\vartheta \tau \}. \end{aligned}$$

Then from the results of Liu et al. (2011), we get the following lemma.

#### Lemma 4.1

For the operator $A_{\tau }$ defined by (2.3), if $\lambda \in \Omega$, then $\lambda \in \rho (A_{\tau })$, where $\rho (A_{\tau })$ is the resolvent set of $A_{\tau }$ and

4.5 $$\begin{aligned} (\lambda I-A_{\tau })^{-1}\left( \begin{array}{{c}} \zeta \\ \delta \\ \end{array} \right) =\left( \begin{array}{{c}} 0_{\mathbb {R}^2}\\ \varphi \\ \end{array} \right)&\Rightarrow (\lambda I-A_{\tau })\left( \begin{array}{{c}} 0_{\mathbb {R}^2}\\ \varphi \\ \end{array} \right) =\left( \begin{array}{{c}} \zeta \\ \delta \\ \end{array} \right) \nonumber \\&\Leftrightarrow \varphi (a)=e^{-\int _{0}^{a}(\lambda I+\tau Q)\textrm{d}l}\zeta \nonumber \\&\quad +\int _{0}^{a}e^{-\int _{s}^{a}(\lambda I+\tau Q)\textrm{d}l}\delta (s)\textrm{d}s \end{aligned}$$

with $$\left( \begin{array}{{c}} \zeta \\ \delta \\ \end{array} \right) \in X$$ and $$\left( \begin{array}{{c}} 0_{\mathbb {R}^2}\\ \varphi \\ \end{array} \right) \in D(A_{\tau })$$. In addition, $A_{\tau }$ is a Hille–Yosida operator and

4.6 $$\begin{aligned} \parallel (\lambda I-A_{\tau })^{-n}\parallel \le \frac{1}{[\textrm{Re}(\lambda )+\vartheta \tau ]^n},\quad \forall \, \lambda \in \Omega , n\ge 1. \end{aligned}$$

Define a linear operator ${{\widehat{A}_{0}:=D(\widehat{A}_{0})\subset X\rightarrow X}}$ with $D(\widehat{A}_{0})=\left\{ \varphi \in W^{1,1}((0,\right. \left. +\infty ),\mathbb {R}^2):\varphi (0)=0\right\}$ and

$$\begin{aligned} {{\widehat{A}_{0}}}\left( \begin{array}{{c}} 0_{\mathbb {R}^{2}}\\ \varphi \\ \end{array} \right) =\left( \begin{array}{{c}} 0_{\mathbb {R}^{2}}\\ \widehat{A}_{0}(\varphi )\\ \end{array} \right) =\left( \begin{array}{{c}} 0_{\mathbb {R}^{2}}\\ -\varphi ^{\prime }-\tau Q\varphi \\ \end{array} \right) . \end{aligned}$$

We know that ${{\widehat{A}_0}}$ is the part of $A_{\tau }$ in $\overline{D(A_{\tau })}$, where $W^{1,1}\left( (0,+\infty ),\mathbb {R}^2\right) =\{\varphi \in L^1\left( (0,+\infty ),\mathbb {R}^2\right) :\varphi ^{\prime }\in L^1\left( (0,+\infty ),\mathbb {R}^2\right) \}$ is a Sobolev space.

Next we study the spectral properties of the linearized equation of (4.1). From Liu et al. (2011) we can get that ${{\Vert T_{\widehat{A}_{0}}(t)\Vert \le e^{-\vartheta t},~~\forall ~t\ge 0.}}$ Thus we have ${{\omega _{0, \textrm{ess}}(\widehat{A}_0)\le \omega _{0}(\widehat{A}_0)\le -\vartheta \tau ,}}$ where the essential growth bound ${{\omega _{0,\textrm{ess}}(\widehat{A}_0)\in (-\infty ,}}{{+\infty )}}$ of $\widehat{A}_0$ is defined by

$$\begin{aligned} {{\omega _{0,\textrm{ess}}(\widehat{A}_0):=\lim _{t \rightarrow \infty }\frac{\text{ ln }(\Vert T_{\widehat{A}_{0}}(t)\Vert _{\textrm{ess}})}{t}.}} \end{aligned}$$

Due to the fact that $\tau DF(\overline{p}_{+})$ is a compact bounded linear operator, by using the perturbation results in Ducrot et al. (2008), we can obtain that ${{\omega _{0,\textrm{ess}}((A_{\tau }+\tau DF(\overline{p}_{+}))_0)}}{{\le -\vartheta \tau <0.}}$ Therefore, we obtain the following lemma.

#### Lemma 4.2

The linear operator $\tilde{A}$ is a Hille–Yosida operator and its part $\tilde{A}_0$ in $Z_0$ satisfies

$$\begin{aligned} \omega _{0,\textrm{ess}}(\tilde{A}_0)<0. \end{aligned}$$

Let $\lambda \in \Omega$. Since $(\lambda I-A_{\tau })$ is invertible, so $(\lambda I-\tilde{A})$ is invertible if and only if $I-\tau DF(\overline{p}_{+})(\lambda I-A_{\tau })^{-1}$ is invertible, where

4.7 $$\begin{aligned} (\lambda I-\tilde{A})^{-1}&=\left[ \lambda I-(A_{\tau }+\tau DF(\overline{p}_{+}))\right] ^{-1}\nonumber \\&=(\lambda I-A_{\tau })^{-1}\left[ I-\tau DF(\overline{p}_{+})(\lambda I-A_{\tau })^{-1}\right] ^{-1}. \end{aligned}$$

Let $$ [I-\tau DF(\overline{p}_{+})(\lambda I-A_{\tau })^{-1}]\left( \begin{array}{{c}} \zeta \\ \varphi \\ \end{array} \right) =\left( \begin{array}{{c}} \gamma \\ \delta \\ \end{array} \right) , $$ that is

$$\begin{aligned} \left( \begin{array}{{c}} \zeta \\ \varphi \\ \end{array} \right) - \tau DF(\overline{p}_{+})(\lambda I-A_{\tau })^{-1}\left( \begin{array}{{c}} \zeta \\ \varphi \\ \end{array} \right) =\left( \begin{array}{{c}} \gamma \\ \delta \\ \end{array} \right) , \end{aligned}$$

then we have

$$\begin{aligned} \left\{ \begin{aligned}&\zeta -{{\tau DB(\overline{w}_{\tau })}}\left( e^{-\int _{0}^{a}(\lambda I+\tau Q)\textrm{d}l}\zeta +\int _{0}^{a}e^{-\int _{s}^{a}(\lambda I+\tau Q)\textrm{d}l}\delta (s)\textrm{d}s\right) =\gamma ,\\&\delta =\varphi ,\\ \end{aligned} \right. \end{aligned}$$

i.e.,

$$\begin{aligned} \left\{ \begin{aligned}&\zeta -{{\tau DB(\overline{w}_{\tau })}}\left( e^{-\int _{0}^{a}(\lambda I+\tau Q)\textrm{d}l}\zeta \right) =\gamma +{{\tau DB(\overline{w}_{\tau })}}\left( \int _{0}^{a}e^{-\int _{s}^{a}(\lambda I+\tau Q)\textrm{d}l}\delta (s)\textrm{d}s\right) ,\\&\delta =\varphi . \end{aligned} \right. \\ \end{aligned}$$

Then combining with ${{DB(\overline{w}_{\tau })}}$ defined in (4.3), we can get that $\Delta (\lambda )\zeta =\gamma +\Gamma (\lambda ,\delta )$ and $\delta =\varphi$, where

4.8 $$\begin{aligned} \begin{aligned} \Delta (\lambda )&=I-{{\tau DB(\overline{w}_{\tau })}}\left( e^{-\int _{0}^{a}(\lambda I+\tau Q)\textrm{d}l}\right) \\&=I-\left( \begin{array}{{cc}} \displaystyle -\frac{s\eta (1-m)\overline{V}\int _{0}^{+\infty }f(a)\overline{u}(a)\textrm{d}a}{\left[ \alpha +(1-m)\overline{V}+s\int _{0}^{+\infty }\overline{u}(a)\textrm{d}a\right] ^2}&{} \frac{\eta (1-m)\int _{0}^{+\infty }f(a)\overline{u}(a)\textrm{d}a\left[ \alpha +s\int _{0}^{+\infty }\overline{u}(a)\textrm{d}a\right] }{\left[ \alpha +(1-m)\overline{V}+s\int _{0}^{+\infty }\overline{u}(a)\textrm{d}a\right] ^2}\\ \frac{s(1-m)\overline{V}\int _{0}^{+\infty }\beta (a)\overline{u}(a)\textrm{d}a}{\left[ \alpha +(1-m)\overline{V}+s\int _{0}^{+\infty }\overline{u}(a)\textrm{d}a\right] ^2}&{} \Lambda -\frac{2r}{K}\overline{V}-\frac{(1-m)\int _{0}^{+\infty }\beta (a)\overline{u}(a)\textrm{d}a \left[ \alpha +s\int _{0}^{+\infty }\overline{u}(a)\textrm{d}a\right] }{\left[ \alpha +(1-m)\overline{V}+s\int _{0}^{+\infty }\overline{u}(a)\textrm{d}a\right] ^2}\\ \end{array} \right) \\&\quad \times \tau \int _{0}^{+\infty }e^{-\int _{0}^{a}(\lambda I+\tau Q)\textrm{d}l}\textrm{d}a-\left( \begin{array}{{cc}} \displaystyle \frac{\eta (1-m)\overline{V}}{\alpha +(1-m)\overline{V}+s\int _{0}^{+\infty }\overline{u}(a)\textrm{d}a}&{}0\\ 0&{}0\\ \end{array} \right) \\&\quad \times \tau \int _{0}^{+\infty }f(a)e^{-\int _{0}^{a}(\lambda I+\tau Q)\textrm{d}l}\textrm{d}a\\&\quad -\left( \begin{array}{{cc}} \displaystyle 0&{}0\\ -\frac{(1-m)\overline{V}}{\alpha +(1-m)\overline{V}+s\int _{0}^{+\infty }\overline{u}(a)\textrm{d}a}&{}0\\ \end{array} \right) \times \tau \int _{0}^{+\infty }\beta (a)e^{-\int _{0}^{a}(\lambda I+\tau Q)\textrm{d}l}\textrm{d}a \end{aligned} \end{aligned}$$

and

4.9 $$\begin{aligned} \Gamma (\lambda ,\delta )={{\tau DB(\overline{w}_{\tau })}}\left( \int _{0}^{a}e^{-\int _{s}^{a}(\lambda I+\tau Q)\textrm{d}l}\delta (s)\text{ d }s\right) . \end{aligned}$$

When $\Delta (\lambda )$ is invertible, we have $\zeta =[\Delta (\lambda )]^{-1}[\gamma +\Gamma (\lambda ,\varphi )].$ From the above argument, we obtain the following lemma.

#### Lemma 4.3

There are two results:

(i) $$\sigma (\tilde{A})\cap \Omega =\sigma _p(\tilde{A})\cap \Omega =\{\lambda \in \Omega :\textrm{det}(\Delta (\lambda ))=0\}$$;
(ii) If $\lambda \in \rho (\tilde{A})\cap \Omega$, then
  $$\begin{aligned}&(\lambda I-\tilde{A})^{-1}\left( \begin{array}{{c}} \zeta \\ \varphi \\ \end{array} \right) =\left( \begin{array}{{c}} 0_{\mathbb {R}^2}\\ \delta \\ \end{array} \right) \\&\quad \Leftrightarrow \delta (a)=e^{-\int _{0}^{a}(\lambda I+\tau Q)\textrm{d}l}[\Delta (\lambda )]^{-1}[\zeta +\Gamma (\lambda ,\delta )]+\int _{0}^{a}e^{-\int _{s}^{a}(\lambda I+\tau Q)\textrm{d}l}{{\varphi (s)}}\textrm{d}s. \end{aligned}$$

#### Proof

Let $\lambda \in \Omega$ and $\text{ det }(\Delta (\lambda ))\ne 0$. Then we can obtain that

$$\begin{aligned}(\lambda I-\tilde{A})^{-1}\left( \begin{array}{{c}} \zeta \\ \varphi \\ \end{array} \right) =(\lambda I-A_{\tau })^{-1}\left[ I-\tau DF(\overline{p}_{+})(\lambda I-A_{\tau })^{-1}\right] ^{-1}\left( \begin{array}{{c}} \zeta \\ \varphi \\ \end{array} \right) =\left( \begin{array}{{c}} 0_{\mathbb {R}^2}\\ \delta \\ \end{array} \right) . \end{aligned}$$

Denote

$$\begin{aligned} \left[ I-\tau DF(\overline{p}_{+})(\lambda I-A_{\tau })^{-1}\right] ^{-1}\left( \begin{array}{{c}} \zeta \\ \varphi \\ \end{array} \right) =\left( \begin{array}{{c}} \tilde{\zeta }\\ \tilde{\varphi }\\ \end{array} \right) , \end{aligned}$$

then we assert $[\Delta (\lambda )]^{-1}[\zeta +\Gamma (\lambda ,\delta )]=\tilde{\zeta }$ and $\varphi =\tilde{\varphi }$. From Lemma 4.1, we obtain

$$\begin{aligned}&(\lambda I-\tilde{A})^{-1}\left( \begin{array}{{c}} \zeta \\ \varphi \\ \end{array} \right) =\left( \begin{array}{{c}} 0_{\mathbb {R}^2}\\ \delta \\ \end{array} \right) \\&\quad \Leftrightarrow \delta (a)=e^{-\int _{0}^{a}(\lambda I+\tau Q)\textrm{d}l}[\Delta (\lambda )]^{-1}[\zeta +\Gamma (\lambda ,\delta )]+\int _{0}^{a}e^{-\int _{s}^{a}(\lambda I+\tau Q)\textrm{d}l}\varphi (s)\textrm{d}s. \end{aligned}$$

We claim that $\{\lambda \in \Omega :\text{ det }(\Delta (\lambda ))\ne 0\}\subset \rho (\tilde{A})\cap \Omega$ and $\sigma (\tilde{A})\cap \Omega \subset \{\lambda \in \Omega :\text{ det }(\Delta (\lambda ))=0\}$. If not, assume $\lambda \in \Omega$ and $\text{ det }(\Delta (\lambda ))=0$, then we can get $$\left( \begin{array}{{c}} 0_{\mathbb {R}^2}\\ \delta \\ \end{array} \right) \in D(A)\backslash \{0_{\mathbb {R}^2}\}$$ such that

4.10 $$\begin{aligned} \tilde{A}\left( \begin{array}{{c}} 0_{\mathbb {R}^2}\\ \delta \\ \end{array} \right) =\lambda \left( \begin{array}{{c}} 0_{\mathbb {R}^2}\\ \delta \\ \end{array} \right) . \end{aligned}$$

In fact,

$$\begin{aligned} \left( \begin{array}{{c}} \zeta \\ \varphi \\ \end{array} \right) =(\lambda I-{{\tilde{A}}})\left( \begin{array}{{c}} 0_{\mathbb {R}^2}\\ \delta \\ \end{array} \right) \Leftrightarrow \left( \begin{array}{{c}} 0_{\mathbb {R}^2}\\ \delta \\ \end{array} \right) =(\lambda I-{{\tilde{A}}})^{-1}\left( \begin{array}{{c}} \zeta \\ \varphi \\ \end{array} \right) . \end{aligned}$$

Hence, we can find a non-zero solution of (4.10) if and only if $$\left( \begin{array}{{c}} \zeta \\ \varphi \\ \end{array} \right) \in Z\backslash \{0_{\mathbb {R}^2}\}$$ satisfies

$$\begin{aligned} \left[ I-\tau DF(\overline{p}_{+})(\lambda I-A_{\tau })^{-1}\right] \times \left( \begin{array}{{c}} \zeta \\ \varphi \\ \end{array} \right) =0, \end{aligned}$$

which is equivalent to $$\left( \begin{array}{{c}} \zeta \\ \varphi \\ \end{array} \right) \ne 0$$ with $$ \left\{ \begin{array}{l} \Delta (\lambda )\zeta =0,\\ \varphi =0.\\ \end{array}\right. $$ On the basis of $\text{ det }(\Delta (\lambda ))=0$, there is a $\zeta \ne 0$ such that $\Delta (\lambda )\zeta =0$. Thus we can get $$\left( \begin{array}{{c}} 0_{\mathbb {R}^2}\\ \delta \\ \end{array} \right) \in D(A_{\tau })\backslash \{0_{\mathbb {R}^2}\}$$ satisfying (4.10) and $\lambda \in \sigma _p(\tilde{A})$, then we have $\{\lambda \in \Omega :\text{ det }(\Delta (\lambda ))=0\}\subset \sigma _p(\tilde{A})$. In summary, $\sigma (\tilde{A})\cap \Omega =\sigma _p(\tilde{A})\cap \Omega =\{\lambda \in \Omega :\text{ det }(\Delta (\lambda ))=0\}$. $\square$

It follows from Assumption 1.1 that

$$\begin{aligned}{} & {} \int _{0}^{+\infty }e^{-\int _{0}^{a}(\lambda I+\tau Q)\textrm{d}l}\textrm{d}a=\left( \begin{array}{{cc}} \displaystyle \frac{1}{\lambda +\tau \mu }&{}\quad 0\\ 0&{}\quad \frac{1}{\lambda +\tau d}\\ \end{array} \right) , \\{} & {} \int _{0}^{+\infty }f(a)e^{-\int _{0}^{a}(\lambda I+\tau Q)\textrm{d}l}\textrm{d}a=\left( \begin{array}{{cc}} \displaystyle \frac{f^*e^{-(\lambda +\tau \mu )}}{\lambda +\tau \mu }&{}\quad 0\\ 0&{}\quad \frac{f^*e^{-(\lambda +\tau d)}}{\lambda +\tau d}\\ \end{array} \right) ,\\{} & {} \int _{0}^{+\infty }\beta (a)e^{-\int _{0}^{a}(\lambda I+\tau Q)\textrm{d}l}\textrm{d}a=\left( \begin{array}{{cc}} \displaystyle \frac{\beta ^*e^{-(\lambda +\tau \mu )}}{\lambda +\tau \mu }&{}\quad 0\\ 0&{}\quad \frac{\beta ^*e^{-(\lambda +\tau d)}}{\lambda +\tau d}\\ \end{array} \right) . \end{aligned}$$

According to (4.3) and (4.8), the characteristic equation at the positive equilibrium can be expressed as:

4.11 $$\begin{aligned}&\text{ det }(\Delta (\lambda ))\nonumber \\ {}&=\left| \begin{array}{{cc}} \displaystyle 1-\tau \frac{\frac{\eta (1-m)\overline{V}}{\alpha +(1-m)\overline{V}+s\overline{U}}\left[ b\mu e^{-\lambda }-\frac{s\overline{Y}}{\alpha +(1-m)\overline{V}+s\overline{U}}\right] }{\lambda +\tau \mu } &{}-\tau \frac{\frac{\eta (1-m)\overline{Y}\left( \alpha +s\overline{U}\right) }{\left[ \alpha +(1-m)\overline{V}+s\overline{U}\right] ^2}}{\lambda +\tau d}\\ \tau \frac{\frac{(1-m)\overline{V}}{\alpha +(1-m)\overline{V}+s\overline{U}}\left[ \mu e^{-\lambda }-\frac{s\overline{Z}}{\alpha +(1-m)\overline{V}+s\overline{U}}\right] }{\lambda +\tau \mu } &{} 1-\tau \frac{\left[ \Lambda -\frac{2r}{K}\overline{V}-\frac{(1-m)\overline{Z} \left( \alpha +s\overline{U}\right) }{\left[ \alpha +(1-m)\overline{V}+s\overline{U}\right] ^2}\right] }{\lambda +\tau d}\\ \end{array} \right| \nonumber \\&=\frac{\lambda ^2+ p_1\lambda \tau +p_0\tau ^2+(s_1\lambda \tau +s_0\tau ^2)e^{-\lambda }}{(\lambda +\tau \mu )(\lambda +\tau d)}:=\frac{\hat{f}(\lambda ,\tau )}{\hat{g}(\lambda ,\tau )}=0. \end{aligned}$$

From (4.11), we have

4.12 $$\begin{aligned} \hat{f}(\lambda ,\tau )=\lambda ^2+ p_1\lambda \tau +p_0\tau ^2+(s_1\lambda \tau +s_0\tau ^2)e^{-\lambda }, \end{aligned}$$

where

$$\begin{aligned} \overline{V}&=\int _{0}^{+\infty }\overline{v}(a)\text{ d }a=\frac{-K(\mu P-s b\eta r)+\sqrt{\Delta }}{2sb\eta r}>0,\\ \overline{Y}&=\int _{0}^{+\infty }f(a)\overline{u}(a)\text{ d }a>0,\\ \overline{U}&=\int _{0}^{+\infty }\overline{u}(a)\text{ d }a=\frac{-PK(\mu P-s b\eta r)+P\sqrt{\Delta }-2sb\eta r\alpha }{2s^2 b\eta r}>0,\\ \overline{Z}&=\int _{0}^{+\infty }\beta (a)\overline{u}(a)\text{ d }a>0,\\ R&=b\eta (1-m)\overline{V}>0,\;\,R_1=\mu P\overline{U}\overline{V}=\frac{\mu P\overline{U}}{b\eta (1-m)}R>0,\\ R_2&=s b\eta \mu \overline{U}\overline{V}=\frac{s\mu \overline{U}}{1-m}R>0,\\ R_3&=\frac{2r}{K}\overline{V}=\frac{2r}{Kb\eta (1-m)}R>0,\;\,P=(b\eta -1)(1-m)>0,\\ s_1&=-\mu ,\;\,s_0=\mu (r-R_3),\\ p_1&=\frac{R^2(\mu +R_3-r)+(1-m)(R_1+R_2)}{R^2},\\ p_0&=\frac{\mu R^2(R_3-r)+(1-m)[\mu R_1+R_2(R_3-r)]}{R^2}. \end{aligned}$$

Let $\lambda =\tau \xi$, then we obtain

$$\begin{aligned} \hat{f}(\tau \xi ,\tau )=\tau ^2\left[ \xi ^2+p_1\xi +p_0+(s_1\xi +s_0)e^{-\tau \xi }\right] :=\tau ^2h(\tau ,\xi ), \end{aligned}$$

where

4.13 $$\begin{aligned} h(\tau ,\xi )=\xi ^2+p_1\xi +p_0+(s_1\xi +s_0)e^{-\tau \xi }. \end{aligned}$$

It is easy to know that $\{\lambda \in \Omega :\text{ det }(\Delta (\lambda ))=0\}=\{\lambda =\tau \xi \in \Omega :h(\tau ,\xi )=0\}$.

#### Remark 4.1

In order to utilize the Hopf bifurcation theory of the non-densely defined cauchy problems (Liu et al. 2011), an inverse transformation $\lambda =\tau \xi$ with $\tau >0$ need to be considered. The main purpose is to use the Routh–Hurwitz criterion to obtain the distribution of the roots for the characteristic equation when $\tau >0$. In fact, Eq. (4.13), that is $h(\tau ,\xi )=\xi ^2+p_1\xi +p_0+(s_1\xi +s_0)e^{-\tau \xi }$, is just the characteristic equation of system (1.4).

### Existence of Hopf bifurcation

From Eq. (4.13), if $\xi =0$, then $h(\tau ,0)=p_0+s_0=R_4$, where

4.14 $$\begin{aligned} R_4=\frac{(1-m)[\mu R_1+R_2(R_3-r)]}{R^2}. \end{aligned}$$

Based on the relationship between $R_1,\;R_2,\;R_3$ and *R*, we can calculate that $R_4>0$, so that $\xi =0$ is not a root of equation (4.13). When $\tau =0$, we have the following result.

#### Theorem 4.1

Suppose that Assumptions 1.1 and 3.1 hold, then the positive equilibrium of system (1.4) is locally asymptotically stable with $\tau =0$.

#### Proof

If $\tau =0$, then the Eq. (4.13) becomes:

$$\begin{aligned} h(0,\xi )=\xi ^2+(p_1+s_1)\xi +(p_0+s_0) \end{aligned}$$

with $p_0+s_0=R_4$ and $p_1+s_1=R_5$, where $R_4>0$ is given in (4.14) and

4.15 $$\begin{aligned} R_5=\frac{R^2(R_3-r)+(1-m)(R_1+R_2)}{R^2}. \end{aligned}$$

Similarly, from the relationship between $R_1,~R_2,~R_3$ and *R*, we can obtain that $R_5>0$. Then, according to the Routh–Hurwitz criterion (Murray 1998), we get that all roots of equation (4.13) have negative real parts with $\tau =0$, and the positive equilibrium of system (1.4) is locally asymptotically stable. This completes the proof of Theorem 4.1. $\square$

If $\tau \ne 0$, assume that $\xi =i\omega (\omega >0)$ is a purely imaginary root of equation (4.13). Substituting $i\omega$ into $h(\tau ,\xi )=0$, we obtain that

4.16 $$\begin{aligned} h(\tau ,i\omega )=-\omega ^2+p_1\omega i+p_0+(s_1\omega i+s_0)e^{-\tau \omega i}=0. \end{aligned}$$

By separating real and imaginary parts, it follows that

4.17 $$\begin{aligned} \left\{ \begin{array}{l} s_{1}\omega \text{ sin }(\omega \tau )+s_{0}\text{ cos }(\omega \tau )=\omega ^2-p_{0},\\ s_{1}\omega \text{ cos }(\omega \tau )-s_{0}\text{ sin }(\omega \tau )=-p_{1}\omega .\\ \end{array} \right. \end{aligned}$$

Squaring two equations in (4.17) and adding them up, we get

4.18 $$\begin{aligned} \omega ^4+(p_{1}^2-2p_{0}-s_{1}^2)\omega ^2+(p_{0}^2-s_{0}^2)=0. \end{aligned}$$

Let $\omega ^2=\tilde{\hbar }$, then Eq. (4.18) can be written as

4.19 $$\begin{aligned} \tilde{\hbar }^2+(p_{1}^2-2p_{0}-s_{1}^2)\tilde{\hbar }+(p_{0}^2-s_{0}^2)=0. \end{aligned}$$

Denote $\tilde{\hbar }_1$ and $\tilde{\hbar }_2$ be the two roots of Eq. (4.19), then we have $\tilde{\hbar }_1+\tilde{\hbar }_2=-(p_{1}^2-2p_{0}-s_{1}^2)$, $\tilde{\hbar }_1\tilde{\hbar }_2=p_{0}^2-s_{0}^2=(p_{0}+s_{0})(p_{0}-s_{0})$. At the same time, Eq. (4.19) has only one positive root $\tilde{\hbar }_0$ when $p_0>0$ and $p_{0}-s_{0}=R_6<0$, where

$$\begin{aligned} R_6=\frac{2\mu R^2(R_3-r)+(1-m)[\mu R_1+R_2(R_3-r)]}{R^2}. \end{aligned}$$

Then Eq. (4.18) has only one positive real root $\omega _0=\sqrt{\tilde{\hbar }_0}$ and $h(\tau ,\xi )=0$ with $\tau =\tau _k,~k=0,1,2,\ldots$ has a pair of purely imaginary roots $\pm i\omega _0$, where

4.20 $$\begin{aligned} \omega _0=\sqrt{\frac{-(p_{1}^2-2p_{0}-s_{1}^2)+\sqrt{(p_{1}^2-2p_{0}-s_{1}^2)^2-4(p_{0}^2-s_{0}^2)}}{2}} \end{aligned}$$

and

$$\begin{aligned} \tau _k=\left\{ \begin{aligned}&\frac{1}{\omega _0}\left[ \text{ arccos }\left( \frac{s_0(\omega _0^2-p_0)-s_1p_1\omega _0^2}{s_1^2\omega _0^2+s_0^2}\right) +2k\pi \right] , \quad \\&\quad \textrm{if}\;\frac{s_1\omega _0(\omega _0^2-p_0)+s_0p_1\omega _0}{s_1^2\omega _0^2+s_0^2}\ge 0,\\&\frac{1}{\omega _0}\left[ -\text{ arccos }\left( \frac{s_0(\omega _0^2-p_0)-s_1p_1\omega _0^2}{s_1^2\omega _0^2+s_0^2}\right) +2(k+1)\pi \right] , \quad \\&\quad \textrm{if}\;\frac{s_1\omega _0(\omega _0^2-p_0)+s_0p_1\omega _0}{s_1^2\omega _0^2+s_0^2}<0, \end{aligned} \right. \end{aligned}$$

for $$k=0,1,2,\ldots $$.

#### Lemma 4.4

Suppose that Assumptions 1.1 and 3.1 hold, then we have $\frac{\textrm{d}h(\tau ,\xi )}{\textrm{d}\xi }\Big |_{\xi =i\omega _0}\ne 0$, where $\xi =i\omega _0$ is a simple root of equation (4.13).

#### Proof

Differentiating equation (4.13) with respect to $\xi$ and noticing that $\xi$ is a function with respect to $\tau$, we obtain

$$\begin{aligned} \frac{\textrm{d}h(\tau ,\xi )}{\textrm{d}\xi }\Big |_{\xi =i\omega _0}=\left\{ 2\xi +p_1+s_1e^{-\tau \xi }-\tau (s_1\xi +s_0)e^{-\tau \xi }\right\} \Big |_{\xi =i\omega _0} \end{aligned}$$

and

$$\begin{aligned} \left\{ 2\xi +p_1+s_1e^{-\tau \xi }-\tau (s_1\xi +s_0)e^{-\tau \xi }\right\} \frac{\textrm{d}\xi (\tau )}{\textrm{d}\tau }=\xi (s_1\xi +s_0)e^{-\tau \xi }. \end{aligned}$$

Assume that $\frac{\textrm{d}h(\tau ,\xi )}{\textrm{d}\xi }\Big |_{\xi =i\omega _0}=0$, then $i\omega _0(s_1\omega _0i+s_0)e^{-\tau \omega _0i}=0.$ Thus, we have

4.21 $$\begin{aligned} \left\{ \begin{array}{l} -s_{1}\omega _0^2 \text{ cos }(\omega _0\tau )+s_{0}\omega _0\text{ sin }(\omega _0\tau )=0,\\ s_{1}\omega _0^2 \text{ sin }(\omega _0\tau )+s_{0}\omega _0\text{ cos }(\omega _0\tau )=0,\\ \end{array} \right. \end{aligned}$$

that is $(s_{1}\omega _0^2)^2+(s_{0}\omega _0)=0,$ so $s_{1}\omega _0^2=s_{0}\omega _0=0.$ Since $\omega _0>0$, we get that $s_{1}=s_{0}=0.$ But we know that $s_1=-\mu <0,$ which leads to a contradiction. Hence, we have $\frac{\textrm{d}h(\tau ,\xi )}{\textrm{d}\xi }\Big |_{\xi =i\omega _0}\ne 0.$ $\square$

#### Lemma 4.5

Suppose that Assumptions 1.1 and 3.1 hold, denote the root of $h(\tau ,\xi )=0$ as $\xi (\tau )=\varrho (\tau )+i\omega (\tau )$ with $\varrho (\hat{\tau }_0)=0$ and $\omega (\hat{\tau }_0)=\omega _0$, where $\hat{\tau }_0=\textrm{min}\{\tau _k\},~k=0,1,2,\ldots$, then the following transversality condition

$$\begin{aligned} \varrho ^{\prime }(\hat{\tau }_0)=\frac{\textrm{dRe}(\xi )}{\textrm{d}\tau }\Big |_{\tau =\hat{\tau }_0}>0 \end{aligned}$$

holds.

#### Proof

Taking the derivative of $\xi$ respect to $\tau$ in Eq. (4.13), we obtain

$$\begin{aligned} \left( \frac{\text{ d }\xi }{\text{ d }\tau }\right) ^{-1}\Big |_{\xi =i\omega _0}= \left\{ \frac{2\xi +p_{1}}{-\xi (\xi ^2+p_{1}\xi +p_{0})}+\frac{s_{1}}{\xi (s_{1}\xi +s_{0})}-\frac{\tau }{\xi }\right\} \Big |_{\xi =i\omega _0}. \end{aligned}$$

Thus, by calculation we have

4.22 $$\begin{aligned} \text{ Re }\left\{ \left( \frac{\text{ d }\xi }{\text{ d }\tau }\right) ^{-1}\Big |_{\xi =i\omega _0}\right\}&=\text{ Re }\left( \frac{2\xi +p_{1}}{-\xi (\xi ^2+p_{1}\xi +p_{0})}\right) _{\xi =i\omega _0}\nonumber \\&\quad +\text{ Re }\left( \frac{s_{1}}{\xi (s_{1}\xi +s_{0})}\right) _{\xi =i\omega _0}\nonumber \\&=\frac{2(\omega _0^2-p_{0})+p_{1}^2}{p_{1}\omega _0^2+(\omega _0^2-p_{0})^2} -\frac{s_{1}^2}{s_{1}\omega _0^2+s_{0}^2}. \end{aligned}$$

From Eqs. (4.20) and (4.22), one has

$$\begin{aligned} sign \left\{ \frac{\text{ d }(\text{ Re }\xi )}{\text{ d }\tau }\Big |_{\xi =i\omega _0}\right\}&=sign \left\{ \text{ Re }\left( \frac{\text{ d }\xi }{\text{ d }\tau }\right) ^{-1}\Big |_{\xi =i\omega _0}\right\} \\&=sign \left\{ \frac{2\omega _0^2+p_{1}^2-2p_{0}-s_1^2}{s_{1}\omega _0^2+s_{0}^2}\right\} \\&=sign \left\{ \frac{\sqrt{(p_{1}^2-2p_{0}-s_{1}^2)^2-4(p_{0}^2-s_{0}^2)}}{s_{1}\omega _0^2+s_{0}^2}\right\} >0. \end{aligned}$$

It follows that $\frac{\textrm{dRe}(\xi )}{\textrm{d}\tau }\Big |_{\tau =\hat{\tau }_0}>0$ and the proof is completed. $\square$

By Lemmas 4.4 and 4.5, we have the following theorem.

#### Theorem 4.2

Suppose that Assumptions 1.1 and 3.1 hold. Then there exists $\hat{\tau }_0=\textrm{min}\{\tau _k\}>0,~k=0,1,2,\ldots ,$ such that

(i) all the roots of equation (4.13) have negative real parts if $\tau \in [0,\hat{\tau }_0)$, which means that the positive equilibrium $(\overline{u}_{+}(a),\overline{V})$ of system (1.5) is locally asymptotically stable;
(ii) Equation (4.13) has at least one root with positive real part if $\tau >\hat{\tau }_0$, then the positive equilibrium $(\overline{u}_{+}(a),\overline{V})$ of system (1.5) is unstable;
(iii) Equation (4.13) has a pair of purely imaginary roots $\pm i\omega _0$ ($\omega _0$ is a positive root of (4.18) if $\tau =\hat{\tau }_0$, which implies that system (1.5) undergoes a Hopf bifurcation at the positive equilibrium $(\overline{u}_{+}(a),\overline{V})$.

## Properties of Hopf bifurcation

In this section, we study the direction of Hopf bifurcation and stability of the bifurcating periodic solutions by applying center manifold theorem (Magal and Ruan 2009a) and normal form theory (Liu et al. 2014; Chu et al. 2016) to Cauchy problem (2.5).

### Transformation of the bifurcation parameter

We first incorporate the parameter $\tau$ into the state space, then the Cauchy problem (2.5) becomes the following system

5.1 $$\begin{aligned} \left\{ \begin{aligned}&\frac{\text{ d } \tau (t)}{\text{ d } t}=0,\quad t\ge 0,\\&\frac{\text{ d } p(t)}{\text{ d } t}={{A_{\tau (t)}p(t)}}+\tau (t)F(p(t)),\quad t\ge 0,\\&\tau (0)=\tau _0\in \mathbb {R},\\&p(0)=\left( \begin{array}{{c}} {{0_{\mathbb {R}^2}}}\\ w_0\\ \end{array} \right) =p_0\in \overline{D(A)}. \end{aligned} \right. \end{aligned}$$

Now let $\hat{p}(t)=p(t)-\overline{p}_{+}$, then system (5.1) takes the form

5.2 $$\begin{aligned} \left\{ \begin{aligned}&\frac{\text{ d } \tau (t)}{\text{ d } t}=0,\quad t\ge 0,\\&\frac{\text{ d } \hat{p}(t)}{\text{ d } t}={{A_{\tau (t)}\hat{p}(t)}}+\tau (t)F(\hat{p}(t)+\overline{p}_{+})-\tau (t)F(\overline{p}_{+}),\quad t\ge 0,\\&\tau (0)=\tau _0\in \mathbb {R},\\&\hat{p}(0)=\left( \begin{array}{{c}} {{0_{\mathbb {R}^2}}}\\ w_0-{{\overline{w}_{\tau }}}\\ \end{array} \right) =\hat{p}_0\in \overline{D(A)}. \end{aligned} \right. \end{aligned}$$

Making a change of variable $\tilde{\tau }(t)=\tau (t)-\tau _k$, then system (5.2) is given by

5.3 $$\begin{aligned} \left\{ \begin{aligned}&\frac{\text{ d } \tilde{\tau }(t)}{\text{ d } t}=0,\quad t\ge 0,\\&\frac{\text{ d } \hat{p}(t)}{\text{ d } t}={{{A}\hat{p}(t)}}+{F}(\tilde{\tau }(t),\hat{p}(t)),\quad t\ge 0,\\&\tilde{\tau }(0)=\tau _0-\tau _k\in \mathbb {R},\\&\hat{p}(0)=\left( \begin{array}{{c}} {{0_{\mathbb {R}^2}}}\\ w_0-{{\overline{w}_{\tau }}}\\ \end{array} \right) =\hat{p}_0\in \overline{D(A)} \end{aligned} \right. \end{aligned}$$

with ${{{A}=A_{\tilde{\tau }(t)+\tau _k}}}$ and ${F}(\tilde{\tau }(t),\hat{p}(t))=(\tilde{\tau }(t)+\tau _k)F(\hat{p}(t)+{\overline{p}_{+}}_{\tilde{\tau }(t)+\tau _k}) -(\tilde{\tau }(t)+\tau _k)F({\overline{p}_{+}}_{\tilde{\tau }(t)+\tau _k})$, so we obtain

$$\begin{aligned}&\partial _{\tilde{\tau }}{F}(\tilde{\tau }(t),\hat{p}(t))(\hat{\tau })\\&\quad =\hat{\tau }\Bigg \{F(\hat{p}(t)+{\overline{p}_{+}}_{\tilde{\tau }(t)+\tau _k}) -F({\overline{p}_{+}}_{\tilde{\tau }(t)+\tau _k})+(\tilde{\tau }(t)+\tau _k)\\&\qquad \Bigg [DF(\hat{p}(t)+{\overline{p}_{+}}_{\tilde{\tau }(t)+\tau _k}) \frac{\text{ d } {\overline{p}_{+}}_{\tilde{\tau }(t)+\tau _k}}{\text{ d } \tilde{\tau }}\\&\qquad -DF({\overline{p}_{+}}_{\tilde{\tau }(t)+\tau _k})\frac{\text{ d } {\overline{p}_{+}}_{\tilde{\tau }(t)+\tau _k}}{\text{ d } \tilde{\tau }} \Bigg ]\Bigg \} \end{aligned}$$

and $\partial _{\hat{p}}{F}(\tilde{\tau }(t),\hat{p}(t))(\check{p})=(\tilde{\tau }(t)+\tau _k)DF(\hat{p}(t) +{\overline{p}_{+}}_{\tilde{\tau }(t)+\tau _k})(\check{p}).$ Therefore, $\partial _{\tilde{\tau }}{F}(0,0)(\hat{\tau })=0$ and $\partial _{\hat{p}}{F}(0,0)(\check{p})=\tau _kDF({\overline{p}_{+}})(\check{p}).$ To rewrite system (5.3) as an abstract Cauchy problem, redefine the Banach space as $\mathcal {X}:=\mathbb {R}\times X$, which has the usual product norm

$$\begin{aligned} \left\| \left( \begin{array}{{c}} \tilde{\tau }\\ \hat{p}\\ \end{array} \right) \right\| =\Vert \tilde{\tau }\Vert _{\mathbb {R}}+\Vert \hat{p}\Vert _{X},~\forall \left( \begin{array}{{c}} \tilde{\tau }\\ \hat{p}\\ \end{array} \right) \in \mathcal {X}. \end{aligned}$$

We consider the linear operator $\mathcal {A}:D(\mathcal {A})\subset \mathcal {X}\rightarrow \mathcal {X}$ defined by

$$\begin{aligned} \mathcal {A}\left( \begin{array}{{c}} \tilde{\tau }\\ \hat{p}\\ \end{array} \right) =\left( \begin{array}{{c}} 0_{\mathbb {R}}\\ ({{A_{\tau _k}}}+\tau _kDF(\overline{p}_{+}))\hat{p}\\ \end{array} \right) =\left( \begin{array}{{c}} 0_{\mathbb {R}}\\ \mathcal {A}_{\tau _k}\hat{p}\\ \end{array} \right) \end{aligned}$$

with $D(\mathcal {A})=\mathbb {R}\times D(A)$, $\overline{D(\mathcal {A})}=\mathbb {R}\times \overline{ D(A)}=\mathcal {X}_{0}$ and $\mathcal {A}_{\tau _k}:={{A_{\tau _k}}}+\tau _kDF({\overline{p}_{+}})$. Because $A_\tau$ is a Hille–Yosida operator, we know that $\mathcal {A}$ is also a Hille–Yosida operator. Moreover, we consider the nonlinear map $\mathcal {P}:\overline{D(\mathcal {A})}\rightarrow \mathcal {X}$ defined by

5.4 $$\begin{aligned} \mathcal {P}\left( \begin{array}{{c}} \tilde{\tau }\\ \hat{p}\\ \end{array} \right) =\left( \begin{array}{{c}} 0_{\mathbb {R}}\\ \mathcal {H}\left( \begin{array}{{c}} \tilde{\tau }\\ \hat{p}\\ \end{array} \right) \\ \end{array} \right) \end{aligned}$$

and the nonlinear map $\mathcal {H}:\overline{D(\mathcal {A})}\rightarrow X$ given by

5.5 $$\begin{aligned} \mathcal {H}\left( \begin{array}{{c}} \tilde{\tau }\\ \hat{p}\\ \end{array} \right)&=(\tilde{\tau }(t)+\tau _k)F(\hat{p}(t)+{\overline{p}_{+}}_{\tilde{\tau }(t)+\tau _k}) -(\tilde{\tau }(t)+\tau _k)F({\overline{p}_{+}}_{\tilde{\tau }(t)+\tau _k})\nonumber \\&\quad -\tau _kDF({\overline{p}_{+}})(\hat{p})-\tilde{\tau }(t)\mathcal {K}(\hat{p}), \end{aligned}$$

where

5.6 $$\begin{aligned} \mathcal {K}(\hat{p})=\mathcal {K}\left( \begin{array}{{c}} 0_{\mathbb {R}^2}\\ \varphi \\ \end{array} \right) =\left( \begin{array}{{c}} 0_{\mathbb {R}^2}\\ Q\varphi \\ \end{array} \right) . \end{aligned}$$

Then we have $$\mathcal {P}\left( \begin{array}{{c}} \tilde{\tau }\\ \hat{p}\\ \end{array} \right) =0$$ and $$D\mathcal {P}\left( \begin{array}{{c}} \tilde{\tau }\\ \hat{p}\\ \end{array} \right) =0$$. Let $$\kappa (t)=\left( \begin{array}{{c}} \tilde{\tau }\\ \hat{p}\\ \end{array} \right) $$, then system (5.3) is rewritten as

5.7 $$\begin{aligned} \left\{ \begin{aligned}&\frac{\text{ d } \kappa (t)}{\text{ d } t}=\mathcal {A}\kappa (t)+\mathcal {P}(\kappa (t)),\quad t\ge 0,\\&\kappa (0)=\kappa _0\in \overline{D(\mathcal {A})}. \end{aligned} \right. \end{aligned}$$

### Spectral decomposition of the state space

In this subsection, we study the spectral properties of the linear operator $\mathcal {A}_{\tau _k}$. Firstly, we calculate the projectors on the generalized eigenspace associated to eigenvalues $\omega _{*}i,-\omega _{*}i$ of $\mathcal {A}_{\tau _k}$. In addition, we get that $\omega _{*}i,-\omega _{*}i$ are poles of $(\lambda I-\mathcal {A}_{\tau _k})^{-1}$ of finite order 1. It implies that $\omega _{*}i,-\omega _{*}i$ are isolated in $\sigma (\mathcal {A}_{\tau _k})\cap \Omega$ and the Laurent’s expansion of the resolvent near $\omega _{*}i,-\omega _{*}i$ takes the form as follows:

$$\begin{aligned} (\lambda I-\mathcal {A}_{\tau _k})^{-1}=\sum _{n=-1}^{+\infty }(\lambda -\lambda _0)^nB_{n,\lambda _0}^{\mathcal {A}_{\tau _k}},\quad \lambda _0 =\omega _{*}i,-\omega _{*}i. \end{aligned}$$

The bounded linear operator $B_{-1,\lambda _0}^{\mathcal {A}_{\tau _k}}$ is the projector on the generalized eigenspace of $\mathcal {A}_{\tau _k}$ related to $\lambda _0 =\omega _{*}i,-\omega _{*}i$. Note that

$$\begin{aligned} (\lambda -\lambda _0)(\lambda I-\mathcal {A}_{\tau _k})^{-1}=\sum _{n_1=0}^{+\infty }(\lambda -\lambda _0)^{n_1}B_{n_1-1,\lambda _0}^{\mathcal {A}_{\tau ^k}},\quad \lambda _0 =\omega _{*}i,-\omega _{*}i, \end{aligned}$$

then we get that the approximation formula

$$\begin{aligned} B_{-1,\lambda _0}^{\mathcal {A}_{\tau _k}}=\lim _{\lambda \rightarrow \lambda _0}(\lambda -\lambda _0)(\lambda I-\mathcal {A}_{\tau _k})^{-1},\quad \lambda _0=\omega _{*}i,-\omega _{*}i. \end{aligned}$$

#### Theorem 5.1

If Assumptions 1.1 and 3.1 are satisfied, then $\omega _{*}i,-\omega _{*}i$ are poles of $(\lambda I-\mathcal {A}_{\tau _k})^{-1}$ of order $\textrm{1}$ with $\tau =\tau _k,~k=0,1,2,\ldots$, and the projector on the generalized eigenspace of $\mathcal {A}_{\tau _k}$ related to the eigenvalues $\omega _{*}i,-\omega _{*}i$ is $$ B_{-1,\lambda _0}^{\mathcal {A}_{\tau _k}}\left( \begin{array}{{c}} \zeta \\ \delta \\ \end{array} \right) =\left( \begin{array}{{c}} 0_{\mathbb {R}^2}\\ \varphi \\ \end{array} \right) $$. Here

5.8 $$\begin{aligned} \varphi (a)&=\left( \frac{\textrm{d}~\textrm{det}(\Delta (\lambda ))}{\textrm{d}\lambda }\right) ^{-1}\left( \begin{array}{{cc}} e^{-(\lambda _0+\tau _k\mu )a}&{}0\\ 0&{}e^{-(\lambda _0+\tau _kd)a}\\ \end{array} \right) \left( \begin{array}{{cc}} \hbar _{\lambda _0,11}&{}\hbar _{\lambda _0,12}\\ \hbar _{\lambda _0,21}&{}\hbar _{\lambda _0,22}\\ \end{array} \right) \nonumber \\&\quad \times \left\{ \zeta +{{\tau _kDB(\overline{w}_{\tau _k})}}\left[ \int _{0}^{a}\left( \begin{array}{{cc}} e^{-(\lambda _0+\tau _k\mu )(a-s)}&{}0\\ 0&{}e^{-(\lambda _0+\tau _kd)(a-s)}\\ \end{array} \right) \delta (s)\textrm{d}s\right] \right\} \end{aligned}$$

with

$$\begin{aligned} \hbar _{\lambda _0,11}&=1-\frac{\tau _k}{\lambda _0+\tau _kd}\left( \Lambda -\frac{2r}{K}\nu -\frac{\mu PP_1}{sb^2\eta ^2m_1\nu }\right) ,\\ \hbar _{\lambda _0,12}&=\frac{\tau _k}{\lambda _0+\tau _kd}\left( \frac{\mu PP_1}{sb\eta m_1\nu }\right) ,\\ \hbar _{\lambda _0,21}&=-\frac{\tau _k}{\lambda _0+\tau _k\mu }\left( \frac{\mu }{b\eta } e^{-\lambda _0}-\frac{\mu P_1}{b^2\eta ^2m_1\nu }\right) ,\\ \hbar _{\lambda _0,22}&=1-\frac{\tau _k}{\lambda _0+\tau _k\mu }\left( \mu e^{-\lambda _0}-\frac{\mu P_1}{b\eta m_1\nu }\right) , \end{aligned}$$

where $\overline{w}_{\tau _k}$ is defined in (ii) of Proposition 3.1 at $\tau =\tau _k$, and

$$\begin{aligned} \nu :=\overline{V}=\frac{-K(\mu P-s\eta br)+\sqrt{\Delta }}{2s\eta b r},\quad P_1=P\nu -\alpha ,\;m_1=1-m. \end{aligned}$$

#### Proof

According to Lemma 4.1, we have

$$\begin{aligned}&(\lambda -\lambda _0)(\lambda I-\mathcal {A}_{\tau _k})^{-1}\left( \begin{array}{{c}} \zeta \\ \delta \\ \end{array} \right) =\left( \begin{array}{{c}} 0_{\mathbb {R}^2}\\ \widetilde{\varphi }\\ \end{array} \right) \\&\quad \Leftrightarrow \widetilde{\varphi }(a)=(\lambda -\lambda _0)e^{-\int _{0}^{a}(\lambda I+\tau _kQ)\textrm{d}l}(\Delta (\lambda ))^{-1}(\zeta +\Gamma (\lambda ,\delta ))\\&\qquad +(\lambda -\lambda _0)\int _{0}^{a}e^{-\int _{s}^{a}(\lambda I+\tau _kQ)\textrm{d}l}\delta (s)\textrm{d}s, \end{aligned}$$

where $\Gamma (\lambda ,\delta )={{\tau _kDB(\overline{w}_{\tau _k})}}\left( \int _{0}^{a}e^{-\int _{s}^{a}(\lambda I+\tau _kQ)\textrm{d}l}\delta (s)\text{ d }s\right)$. Then we get

$$\begin{aligned}&\lim _{\lambda \rightarrow \lambda _0}(\lambda -\lambda _0)(\lambda I-\mathcal {A}_{\tau _k})^{-1}\left( \begin{array}{{c}} \zeta \\ \delta \\ \end{array} \right) \\&\quad =\lim _{\lambda \rightarrow \lambda _0}(\lambda -\lambda _0)e^{-\int _{0}^{a}(\lambda I+\tau _kQ)\textrm{d}l}(\Delta (\lambda ))^{-1}(\zeta +\Gamma (\lambda ,\delta ))\\&\qquad +(\lambda -\lambda _0)\int _{0}^{a}e^{-\int _{s}^{a}(\lambda I+\tau _kQ)\textrm{d}l}\delta (s)\text{ d }s\\&\quad =\lim _{\lambda \rightarrow \lambda _0}(\lambda -\lambda _0)e^{-\int _{0}^{a}(\lambda I+\tau _kQ)\textrm{d}l}\frac{(\Delta (\lambda ))^{*}}{\text{ det }(\Delta (\lambda ))}\\&\qquad \times \left\{ \zeta +{{\tau _kDB(\overline{w}_{\tau _k})}} \left( \int _{0}^{a}e^{-\int _{s}^{a}(\lambda I+\tau _kQ)\textrm{d}l}\delta (s)\text{ d }s\right) \right\} \\&\quad =\lim _{\lambda \rightarrow \lambda _0}\frac{(\lambda -\lambda _0)}{\text{ det }(\Delta (\lambda ))}e^{-\int _{0}^{a}(\lambda I+\tau _kQ)\textrm{d}l}{{(\Delta (\lambda ))^{*}}}\\&\qquad \times \left\{ \zeta +{{\tau _kDB(\overline{w}_{\tau _k})}} \left( \int _{0}^{a}e^{-\int _{s}^{a}(\lambda I+\tau _kQ)\textrm{d}l}\delta (s)\text{ d }s\right) \right\} \\&\quad =\left( \frac{\textrm{d}~\text{ det }(\Delta (\lambda ))}{\textrm{d}\lambda }\right) ^{-1}\left( \begin{array}{{cc}} e^{-(\lambda _0+\tau _k\mu )a}&{}0\\ 0&{}e^{-(\lambda _0+\tau _kd)a}\\ \end{array} \right) \left( \begin{array}{{cc}} \hbar _{\lambda _0,11}&{}\hbar _{\lambda _0,12}\\ \hbar _{\lambda _0,21}&{}\hbar _{\lambda _0,22}\\ \end{array} \right) \\&\qquad \times \left\{ \zeta +{{\tau _kDB(\overline{w}_{\tau _k})}}\left[ \int _{0}^{a}\left( \begin{array}{{cc}} e^{-(\lambda _0+\tau _k\mu )(a-s)}&{}0\\ 0&{}e^{-(\lambda _0+\tau _kd)(a-s)}\\ \end{array} \right) \delta (s)\text{ d }s\right] \right\} , \end{aligned}$$

where $(\Delta (\lambda ))^{*}$ is the adjoint matrix of $\Delta (\lambda )$ and is expressed as $$(\Delta (\lambda ))^{*}=\left( \begin{array}{{cc}} \hbar _{\lambda _0,11}&{}\hbar _{\lambda _0,12}\\ \hbar _{\lambda _0,21}&{}\hbar _{\lambda _0,22}\\ \end{array} \right) $$ with $\hbar _{\lambda _0,11},~\hbar _{\lambda _0,12},~\hbar _{\lambda _0,21}$ and $\hbar _{\lambda _0,22}$ given in Theorem 5.1. The proof is completed. $\square$

Next, we discuss the state space decomposition regarding the spectral properties of the linear operator $\mathcal {A}_{\tau _k}$. The projector on the linear center manifold is defined by

$$\begin{aligned} \Pi _c^{\mathcal {A}_{\tau _k}}\left( \begin{array}{{c}} \zeta \\ \delta \\ \end{array} \right) =B_{-1,\omega _*i}^{\mathcal {A}_{\tau _k}}\left( \begin{array}{{c}} \zeta \\ \delta \\ \end{array} \right) +B_{-1,-\omega _*i}^{\mathcal {A}_{\tau _k}}\left( \begin{array}{{c}} \zeta \\ \delta \\ \end{array} \right) ,\;\forall \left( \begin{array}{{c}} \zeta \\ \delta \\ \end{array} \right) \in X. \end{aligned}$$

Then correspondingly, we have

$$\begin{aligned} \sigma \left( \mathcal {A}_{\tau _k}|_{\Pi _c^{\mathcal {A}_{\tau _k}}(X)}\right) =\{\omega _*i,-\omega _*i\},\; \sigma \left( \mathcal {A}_{\tau _k}|_{I-\Pi _c^{\mathcal {A}_{\tau _k}}(X)}\right) = \sigma \left( \mathcal {A}_{\tau _k}\right) \backslash \{\omega _*i,-\omega _*i\}. \end{aligned}$$

For the convenience of application in this paper, let

$$\begin{aligned} \Theta _1=\left( \begin{array}{{c}} \left( \begin{array}{{c}} 1\\ 0\\ \end{array} \right) \\ 0_{L^1((0,+\infty ),\mathbb {R}^2)}\\ \end{array} \right) \quad \text{ and }\quad \Theta _2=\left( \begin{array}{{c}} \left( \begin{array}{{c}} 0\\ 1\\ \end{array} \right) \\ 0_{L^1((0,+\infty ),\mathbb {R}^2)}\\ \end{array} \right) . \end{aligned}$$

Then,

$$\begin{aligned} B_{-1,\lambda _0}^{\mathcal {A}_{\tau _k}}\Theta _1=\left( \begin{array}{{c}} 0_{\mathbb {R}^2}\\ \left( \frac{\text{ d }~\text{ det }(\Delta (\lambda _0))}{\text{ d }\lambda }\right) ^{-1}\left( \begin{array}{{c}} e^{-(\lambda _0+\tau _k\mu )a}\hbar _{\lambda _0,11}\\ e^{-(\lambda _0+\tau _kd)a}\hbar _{\lambda _0,21}\\ \end{array} \right) \\ \end{array} \right) \end{aligned}$$

and

$$\begin{aligned} B_{-1,\lambda _0}^{\mathcal {A}_{\tau _k}}\Theta _2=\left( \begin{array}{{c}} 0_{\mathbb {R}^2}\\ \left( \frac{\text{ d }~\text{ det }(\Delta (\lambda _0))}{\text{ d }\lambda }\right) ^{-1}\left( \begin{array}{{c}} e^{-(\lambda _0+\tau _k\mu )a}\hbar _{\lambda _0,12}\\ e^{-(\lambda _0+\tau _kd)a}\hbar _{\lambda _0,22}\\ \end{array} \right) \\ \end{array} \right) . \end{aligned}$$

Let $\beta _1=e^{-(\omega _*i+\tau _k\mu )a},~\beta _2=e^{-(\omega _*i+\tau _kd)a},~\beta _3=e^{-(-\omega _*i+\tau _k\mu )a},~\beta _4=e^{-(-\omega _*i+\tau _kd)a}.$ Then we have

5.9 $$\begin{aligned} \Pi _c^{\mathcal {A}_{\tau _k}}\Theta _1=B_{-1,\omega _*i}^{\mathcal {A}_{\tau _k}}\Theta _1+B_{-1,-\omega _*i}^{\mathcal {A}_{\tau _k}} \Theta _1=\left( \begin{array}{{c}} 0_{\mathbb {R}^2}\\ \varphi _1(a)\\ \end{array} \right) \end{aligned}$$

with

$$\begin{aligned} \varphi _1(a)&=\left( \frac{\text{ d }~\text{ det }(\Delta (\omega _*i))}{\text{ d }\lambda }\right) ^{-1}\left( \begin{array}{{c}} \beta _1\hbar _{\lambda _0=\omega _*i,11}\\ \beta _2\hbar _{\lambda _0=\omega _*i,21}\\ \end{array} \right) \\&\quad +\left( \frac{\text{ d }~\text{ det }(\Delta (-\omega _*i))}{\text{ d }\lambda }\right) ^{-1}\left( \begin{array}{{c}} \beta _3\hbar _{\lambda _0=-\omega _*i,11}\\ \beta _4\hbar _{\lambda _0=-\omega _*i,21}\\ \end{array} \right) \\&={\textrm{Re}}\left( \left( \frac{\text{ d }~\text{ det }(\Delta (\omega _*i))}{\text{ d }\lambda }\right) ^{-1}\right) \left( \begin{array}{{c}} (\beta _1+\beta _3)\hbar _{\lambda _0=\omega _*i,11}\\ (\beta _2+\beta _4)\hbar _{\lambda _0=\omega _*i,21}\\ \end{array} \right) \\&\quad +{\textrm{Im}}\left( \left( \frac{\text{ d }~\text{ det }(\Delta (\omega _*i))}{\text{ d }\lambda }\right) ^{-1}\right) \left( \begin{array}{{c}} \frac{1}{i}(\beta _1-\beta _3)\hbar _{\lambda _0=\omega _*i,11}\\ \frac{1}{i}(\beta _2-\beta _4)\hbar _{\lambda _0=\omega _*i,21}\\ \end{array} \right) \\ \end{aligned}$$

and

5.10 $$\begin{aligned} \Pi _c^{\mathcal {A}_{\tau _k}}\Theta _2=B_{-1,\omega _*i}^{\mathcal {A}_{\tau _k}}\Theta _2+B_{-1,-\omega _*i}^{\mathcal {A}_{\tau _k}} \Theta _2=\left( \begin{array}{{c}} 0_{\mathbb {R}^2}\\ \varphi _2(a)\\ \end{array} \right) , \end{aligned}$$

where

$$\begin{aligned} \varphi _2(a)&=\left( \frac{\text{ d }~\text{ det }(\Delta (\omega _*i))}{\text{ d }\lambda }\right) ^{-1}\left( \begin{array}{{c}} \beta _1\hbar _{\lambda _0=\omega _*i,12}\\ \beta _2\hbar _{\lambda _0=\omega _*i,22}\\ \end{array} \right) \\&\quad +\left( \frac{\text{ d }~\text{ det }(\Delta (-\omega _*i))}{\text{ d }\lambda }\right) ^{-1}\left( \begin{array}{{c}} \beta _3\hbar _{\lambda _0=-\omega _*i,12}\\ \beta _4\hbar _{\lambda _0=-\omega _*i,22}\\ \end{array} \right) \\&={\textrm{Re}}\left( \left( \frac{\text{ d }~\text{ det }(\Delta (\omega _*i))}{\text{ d }\lambda }\right) ^{-1}\right) \left( \begin{array}{{c}} (\beta _1+\beta _3)\hbar _{\lambda _0=\omega _*i,12}\\ (\beta _2+\beta _4)\hbar _{\lambda _0=\omega _*i,22}\\ \end{array} \right) \\&\quad +{\textrm{Im}}\left( \left( \frac{\text{ d }~\text{ det }(\Delta (\omega _*i))}{\text{ d }\lambda }\right) ^{-1}\right) \left( \begin{array}{{c}} \frac{1}{i}(\beta _1-\beta _3)\hbar _{\lambda _0=\omega _*i,12}\\ \frac{1}{i}(\beta _2-\beta _4)\hbar _{\lambda _0=\omega _*i,22}\\ \end{array} \right) .\\ \end{aligned}$$

Thus, we demonstrate that a basis of $X_c^{\mathcal {A}_{\tau _k}}=\Pi _c^{\mathcal {A}_{\tau _k}}(X)$ is

$$\begin{aligned} \left\{ \left( \begin{array}{{c}} 0_{\mathbb {R}^2}\\ \left( \begin{array}{{c}} \beta _1+\beta _3\\ \beta _2+\beta _4\\ \end{array} \right) \\ \end{array} \right) ,\left( \begin{array}{{c}} 0_{\mathbb {R}^2}\\ \left( \begin{array}{{c}} \frac{1}{i}(\beta _1-\beta _3)\\ \frac{1}{i}(\beta _2-\beta _4)\\ \end{array} \right) \\ \end{array} \right) \right\} . \end{aligned}$$

By construction, we get

$$\begin{aligned} \mathcal {A}_{\tau _k}\left( \begin{array}{{c}} 0_{\mathbb {R}^2}\\ \left( \begin{array}{{c}} e^{-(\pm \omega _*i+\tau _k\mu )a}\\ e^{-(\pm \omega _*i+\tau _kd)a}\\ \end{array} \right) \end{array} \right) =\pm \omega _*i\left( \begin{array}{{c}} 0_{\mathbb {R}^2}\\ \left( \begin{array}{{c}} e^{-(\pm \omega _*i+\tau _k\mu )a}\\ e^{-(\pm \omega _*i+\tau _kd)a}\\ \end{array} \right) \end{array} \right) . \end{aligned}$$

At the same time, we have

$$\begin{aligned} \mathcal {A}_{\tau _k}\left( \begin{array}{{c}} 0_{\mathbb {R}^2}\\ \left( \begin{array}{{c}} \beta _1+\beta _3\\ \beta _2+\beta _4\\ \end{array} \right) \\ \end{array} \right) =\omega _*\left( \begin{array}{{c}} 0_{\mathbb {R}^2}\\ \left( \begin{array}{{c}} \frac{1}{i}(\beta _1-\beta _3)\\ \frac{1}{i}(\beta _2-\beta _4)\\ \end{array} \right) \\ \end{array} \right) \end{aligned}$$

and

$$\begin{aligned} \mathcal {A}_{\tau _k}\left( \begin{array}{{c}} 0_{\mathbb {R}^2}\\ \left( \begin{array}{{c}} \frac{1}{i}(\beta _1-\beta _3)\\ \frac{1}{i}(\beta _2-\beta _4)\\ \end{array} \right) \\ \end{array} \right) =-\omega _*\left( \begin{array}{{c}} 0_{\mathbb {R}^2}\\ \left( \begin{array}{{c}} \beta _1+\beta _3\\ \beta _2+\beta _4\\ \end{array} \right) \\ \end{array} \right) . \end{aligned}$$

Then we have that the matrix of $\mathcal {A}_{\tau _k}|_{\Pi _c^{\mathcal {A}_{\tau _k}}(X)}$ with basis

$$\begin{aligned} \left\{ \left( \begin{array}{{c}} 0_{\mathbb {R}^2}\\ \left( \begin{array}{{c}} \beta _1+\beta _3\\ \beta _2+\beta _4\\ \end{array} \right) \\ \end{array} \right) ,\left( \begin{array}{{c}} 0_{\mathbb {R}^2}\\ \left( \begin{array}{{c}} \frac{1}{i}(\beta _1-\beta _3)\\ \frac{1}{i}(\beta _2-\beta _4)\\ \end{array} \right) \\ \end{array} \right) \right\} \end{aligned}$$

is

$$\begin{aligned} \mathcal {A}_{\tau _k}|_{\Pi _c^{\mathcal {A}_{\tau _k}}(X)}=\left( \begin{array}{{cc}} 0&{}-\omega _*\\ \omega _*&{}0\\ \end{array} \right) . \end{aligned}$$

Let $\Pi _h^{\mathcal {A}_{\tau _k}}=I-\Pi _c^{\mathcal {A}_{\tau _k}},~X_c^{\mathcal {A}_{\tau _k}}=\Pi _c^{\mathcal {A}_{\tau _k}}(X),~ X_h^{\mathcal {A}_{\tau _k}}=\Pi _h^{\mathcal {A}_{\tau _k}}(X)$, then we get

5.11 $$\begin{aligned} \Pi _h^{\mathcal {A}_{\tau _k}}\Theta _1&=(I-\Pi _c^{\mathcal {A}_{\tau _k}})\Theta _1=\left( \begin{array}{{c}} \left( \begin{array}{{c}} 1\\ 0\\ \end{array} \right) \\ -\varphi _1(a)\\ \end{array} \right) , \end{aligned}$$

5.12 $$\begin{aligned} \Pi _h^{\mathcal {A}_{\tau _k}}\Theta _2&=(I-\Pi _c^{\mathcal {A}_{\tau _k}})\Theta _2=\left( \begin{array}{{c}} \left( \begin{array}{{c}} 0\\ 1\\ \end{array} \right) \\ -\varphi _2(a)\\ \end{array} \right) . \end{aligned}$$

Put $\lambda \in i\mathbb {R}\backslash \{-\omega _*i,\omega _*i\}$, then for each $\lambda \in \rho (\mathcal {A}_{\tau _k})$,

5.13 $$\begin{aligned} (\lambda I-\mathcal {A}_{\tau _k})^{-1}\left( \begin{array}{{c}} 0_{\mathbb {R}^2}\\ \left( \begin{array}{{c}} e^{-(\pm \omega _*i+\tau _k\mu )a}\\ e^{-(\pm \omega _*i+\tau _kd)a}\\ \end{array} \right) \end{array} \right) =(\lambda \mp \omega _*i)^{-1}\left( \begin{array}{{c}} 0_{\mathbb {R}^2}\\ \left( \begin{array}{{c}} e^{-(\pm \omega _*i+\tau _k\mu )a}\\ e^{-(\pm \omega _*i+\tau _kd)a}\\ \end{array} \right) \end{array} \right) . \end{aligned}$$

According to (5.11) and (5.13), we can get

5.14 $$\begin{aligned}&\left( \lambda I-\mathcal {A}_{\tau _k}^{\mathbb {C}}|_{\Pi _c^{\mathcal {A}_{\tau _k}}(X)}\right) ^{-1}\Pi _h^{\mathcal {A}_{\tau _k}} \Theta _1=\left( \lambda I-\mathcal {A}_{\tau _k}^{\mathbb {C}}|_{\Pi _c^{\mathcal {A}_{\tau _k}}(X)}\right) ^{-1}\Theta _1\nonumber \\&\quad +\left( \lambda I-\mathcal {A}_{\tau _k}^{\mathbb {C}}|_{\Pi _c^{\mathcal {A}_{\tau _k}}(X)}\right) ^{-1}\left( \begin{array}{{c}} 0_{\mathbb {R}^2}\\ -\varphi _1(a)\\ \end{array} \right) =\left( \begin{array}{{c}} 0_{\mathbb {R}^2}\\ \left( \begin{array}{{c}} \varphi _{11}(\lambda )\\ \varphi _{12}(\lambda )\\ \end{array} \right) \end{array} \right) , \end{aligned}$$

where

$$\begin{aligned} \varphi _{11}(\lambda )&=\frac{e^{-(\lambda +\tau _k\mu )a}\hbar _{\lambda ,11}}{\text{ det }(\Delta (\lambda ))} -\left( \frac{\text{ d }~\text{ det }(\Delta (\omega _*i))}{\text{ d }\lambda }\right) ^{-1} \frac{\beta _1\hbar _{\lambda _0=\omega _*i,11}}{\lambda -\omega _*i}\\&\quad -\left( \frac{\text{ d }~\text{ det }(\Delta (-\omega _*i))}{\text{ d }\lambda }\right) ^{-1} \frac{\beta _3\hbar _{\lambda _0=-\omega _*i,11}}{\lambda +\omega _*i},\\ \varphi _{12}(\lambda )&=\frac{e^{-(\lambda +\tau _kd)a}\hbar _{\lambda ,11}}{\text{ det }(\Delta (\lambda ))} -\left( \frac{\text{ d }~\text{ det }(\Delta (\omega _*i))}{\text{ d }\lambda }\right) ^{-1} \frac{\beta _2\hbar _{\lambda _0=\omega _*i,11}}{\lambda -\omega _*i}\\&\quad -\left( \frac{\text{ d }~\text{ det }(\Delta (-\omega _*i))}{\text{ d }\lambda }\right) ^{-1} \frac{\beta _4\hbar _{\lambda _0=-\omega _*i,11}}{\lambda +\omega _*i}. \end{aligned}$$

In addition, according to (5.12) and (5.13), we can get

5.15 $$\begin{aligned}&\left( \lambda I-\mathcal {A}_{\tau _k}^{\mathbb {C}}|_{\Pi _c^{\mathcal {A}_{\tau _k}}(X)}\right) ^{-1}\Pi _h^{\mathcal {A}_{\tau _k}}\Theta _2=\left( \lambda I-\mathcal {A}_{\tau _k}^{\mathbb {C}}|_{\Pi _c^{\mathcal {A}_{\tau _k}}(X)}\right) ^{-1}\Theta _2 \nonumber \\&\quad +\left( \lambda I-\mathcal {A}_{\tau _k}^{\mathbb {C}}|_{\Pi _c^{\mathcal {A}_{\tau _k}}(X)}\right) ^{-1}\left( \begin{array}{{c}} 0_{\mathbb {R}^2}\\ -\varphi _2(a)\\ \end{array} \right) =\left( \begin{array}{{c}} 0_{\mathbb {R}^2}\\ \left( \begin{array}{{c}} \varphi _{21}(\lambda )\\ \varphi _{22}(\lambda )\\ \end{array} \right) \end{array} \right) , \end{aligned}$$

where

$$\begin{aligned} \varphi _{21}(\lambda )&=\frac{e^{-(\lambda +\tau _k\mu )a}\hbar _{\lambda ,12}}{\text{ det }(\Delta (\lambda ))} -\left( \frac{\text{ d }~\text{ det }(\Delta (\omega _*i))}{\text{ d }\lambda }\right) ^{-1} \frac{\beta _1\hbar _{\lambda _0=\omega _*i,12}}{\lambda -\omega _*i}\\&\quad -\left( \frac{\text{ d }~\text{ det }(\Delta (-\omega _*i))}{\text{ d }\lambda }\right) ^{-1} \frac{\beta _3\hbar _{\lambda _0=-\omega _*i,12}}{\lambda +\omega _*i},\\ \varphi _{22}(\lambda )&=\frac{e^{-(\lambda +\tau _kd)a}\hbar _{\lambda ,12}}{\text{ det }(\Delta (\lambda ))} -\left( \frac{\text{ d }~\text{ det }(\Delta (\omega _*i))}{\text{ d }\lambda }\right) ^{-1} \frac{\beta _2\hbar _{\lambda _0=\omega _*i,12}}{\lambda -\omega _*i}\\&\quad -\left( \frac{\text{ d }~\text{ det }(\Delta (-\omega _*i))}{\text{ d }\lambda }\right) ^{-1} \frac{\beta _4\hbar _{\lambda _0=-\omega _*i,12}}{\lambda +\omega _*i}. \end{aligned}$$

From (5.8), we have $$ B_{-1,\omega _*i}^{\mathcal {A}_{\tau _k}}\left( \begin{array}{{c}} 0_{\mathbb {R}^2}\\ Q\left( \begin{array}{{c}} \beta _1\\ \beta _2\\ \end{array} \right) \\ \end{array} \right) =\left( \begin{array}{{c}} 0_{\mathbb {R}^2}\\ \varphi _{111}(a)\left( \begin{array}{{c}} \beta _1\\ \beta _2\\ \end{array} \right) \\ \end{array} \right) ,$$ where

$$\begin{aligned} \varphi _{111}(a)&=\tau _k\left( \frac{\text{ d }~\text{ det }(\Delta (\omega _*i))}{\text{ d }\lambda }\right) ^{-1} \left\{ \hbar _{\lambda _0=\omega _*i,11}\left[ \frac{d\hbar _1}{(\omega _*i+\tau _kd)^2}+\frac{b\mu e^{-\omega _*ia}}{(\omega _*i+\tau _k\mu )^2}\right] \right. \\&\left. \quad +\hbar _{\lambda _0=\omega _*i,12}\left[ \frac{d\hbar _2}{(\omega _*i+\tau _kd)^2}-\frac{b\mu }{\eta (\omega _*i+\tau _k\mu )^2}\right] \right\} \\ \end{aligned}$$

with

$$\begin{aligned} \hbar _1&=\frac{\mu PP_1}{sb\eta m_1\nu },\\ \hbar _2&=\frac{-2srb^2\eta ^2\nu ^2+[Krsb^2\eta ^2-\mu K(b\eta -1)P]\nu +\mu K(b\eta -1)\alpha }{K\eta ^2\nu bs}. \end{aligned}$$

On the other hand, we deduce that $$B_{-1,-\omega _*i}^{\mathcal {A}_{\tau _k}}\left( \begin{array}{{c}} 0_{\mathbb {R}^2}\\ Q\left( \begin{array}{{c}} \beta _1\\ \beta _2\\ \end{array} \right) \\ \end{array} \right) =\left( \begin{array}{{c}} 0_{\mathbb {R}^2}\\ \varphi _{112}(a)\left( \begin{array}{{c}} \beta _3\\ \beta _4\\ \end{array} \right) \\ \end{array} \right) , $$ where

$$\begin{aligned} \varphi _{112}(a)&=-\tau _k\left( \frac{\text{ d }~\text{ det }(\Delta (-\omega _*i))}{\text{ d }\lambda }\right) ^{-1}\\&\quad \times \left\{ -\hbar _{\lambda _0=-\omega _*i,11}\left[ \frac{d\hbar _1i}{2\omega _*}\left( \frac{1}{\omega _*i+\tau _kd}+\frac{1}{\omega _*i-\tau _kd}\right) \right. \right. \\&\quad \left. +\frac{b\mu i}{2\omega _*}\left( \frac{e^{-\omega _*ia}}{\omega _*i+\tau _k\mu }+\frac{e^{\omega _*ia}}{\omega _*i-\tau _k\mu }\right) \right] \\&\quad -\hbar _{\lambda _0=-\omega _*i,12}\left[ \frac{d\hbar _2i}{2\omega _*}\left( \frac{1}{\omega _*i+\tau _kd}+\frac{1}{\omega _*i-\tau _kd}\right) \right. \\&\left. \left. \quad -\frac{b\mu i}{2\eta \omega _*}\left( \frac{1}{\omega _*i+\tau _k\mu }+\frac{1}{\omega _*i-\tau _k\mu }\right) \right] \right\} .\\ \end{aligned}$$

Therefore, we obtain that

5.16 $$\begin{aligned}&\Pi _c^{\mathcal {A}_{\tau _k}}\left( \begin{array}{{c}} 0_{\mathbb {R}^2}\\ Q\left( \begin{array}{{c}} \beta _1\\ \beta _2\\ \end{array} \right) \\ \end{array} \right) =\varphi _{111}(a)\left( \begin{array}{{c}} 0_{\mathbb {R}^2}\\ \left( \begin{array}{{c}} \beta _1\\ \beta _2\\ \end{array} \right) \\ \end{array} \right) +\varphi _{112}(a)\left( \begin{array}{{c}} 0_{\mathbb {R}^2}\\ \left( \begin{array}{{c}} \beta _3\\ \beta _4\\ \end{array} \right) \\ \end{array} \right) , \end{aligned}$$

5.17 $$\begin{aligned}&\Pi _h^{\mathcal {A}_{\tau _k}}\left( \begin{array}{{c}} 0_{\mathbb {R}^2}\\ Q\left( \begin{array}{{c}} \beta _1\\ \beta _2\\ \end{array} \right) \\ \end{array} \right) =(I-\Pi _c^{\mathcal {A}_{\tau _k}})\left( \begin{array}{{c}} 0_{\mathbb {R}^2}\\ Q\left( \begin{array}{{c}} \beta _1\\ \beta _2\\ \end{array} \right) \\ \end{array} \right) =\left( \begin{array}{{c}} 0_{\mathbb {R}^2}\\ \left( \begin{array}{{c}} \varphi _{121}(a)\\ \varphi _{122}(a)\\ \end{array} \right) \\ \end{array} \right) \end{aligned}$$

with $$\varphi _{121}(a)=(\mu -\varphi _{111}(a))\beta _1-\varphi _{112}(a)\beta _3\;\text{ and }\; \varphi _{122}(a)=(d-\varphi _{111}(a))\beta _2-\varphi _{112}(a)\beta _4.$$

Similarly, from (5.8) we find $$B_{-1,\omega _*i}^{\mathcal {A}_{\tau _k}}\left( \begin{array}{{c}} 0_{\mathbb {R}^2}\\ Q\left( \begin{array}{{c}} \beta _3\\ \beta _4\\ \end{array} \right) \\ \end{array} \right) =\left( \begin{array}{{c}} 0_{\mathbb {R}^2}\\ \varphi _{131}(a)\left( \begin{array}{{c}} \beta _1\\ \beta _2\\ \end{array} \right) \\ \end{array} \right) , $$ where

$$\begin{aligned} \varphi _{131}(a)&=\tau _k\left( \frac{\text{ d }~\text{ det }(\Delta (\omega _*i))}{\text{ d }\lambda }\right) ^{-1}\\&\quad \times \left\{ \hbar _{\lambda _0=\omega _*i,11}\left[ \frac{d\hbar _1i}{2\omega _*}\left( \frac{1}{\omega _*i+\tau _kd}+\frac{1}{\omega _*i-\tau _kd}\right) \right. \right. \\&\quad \left. +\frac{b\mu i}{2\omega _*}\left( \frac{e^{-\omega _*ia}}{\omega _*i+\tau _k\mu }+\frac{e^{\omega _*ia}}{\omega _*i-\tau _k\mu }\right) \right] \\&\quad +\hbar _{\lambda _0=\omega _*i,12}\left[ \frac{d\hbar _2i}{2\omega _*}\left( \frac{1}{\omega _*i+\tau _kd}+\frac{1}{\omega _*i-\tau _kd}\right) \right. \\&\left. \left. \quad -\frac{b\mu i}{2\eta \omega _*}\left( \frac{1}{\omega _*i+\tau _k\mu }+\frac{1}{\omega _*i-\tau _k\mu }\right) \right] \right\} .\\ \end{aligned}$$

On the other hand, we get $$B_{-1,-\omega _*i}^{\mathcal {A}_{\tau _k}}\left( \begin{array}{{c}} 0_{\mathbb {R}^2}\\ Q\left( \begin{array}{{c}} \beta _3\\ \beta _4\\ \end{array} \right) \\ \end{array} \right) =\left( \begin{array}{{c}} 0_{\mathbb {R}^2}\\ \varphi _{132}(a)\left( \begin{array}{{c}} \beta _3\\ \beta _4\\ \end{array} \right) \\ \end{array} \right) , $$ where

$$\begin{aligned} \varphi _{132}(a)&=-\tau _k\left( \frac{\text{ d }~\text{ det }(\Delta (-\omega _*i))}{\text{ d }\lambda }\right) ^{-1}\\&\quad \times \left\{ -\hbar _{\lambda _0=-\omega _*i,11}\left[ \frac{d\hbar _1}{(\omega _*i+\tau _kd)^2}+\frac{b\mu e^{-\omega _*ia}}{(\omega _*i+\tau _k\mu )^2}\right] \right. \\&\left. \quad -\hbar _{\lambda _0=-\omega _*i,12}\left[ \frac{d\hbar _2}{(\omega _*i+\tau _kd)^2}-\frac{b\mu }{\eta (\omega _*i+\tau _k\mu )^2}\right] \right\} .\\ \end{aligned}$$

So we have

5.18 $$\begin{aligned}&\Pi _c^{\mathcal {A}_{\tau _k}}\left( \begin{array}{{c}} 0_{\mathbb {R}^2}\\ Q\left( \begin{array}{{c}} \beta _3\\ \beta _4\\ \end{array} \right) \\ \end{array} \right) =\varphi _{131}(a)\left( \begin{array}{{c}} 0_{\mathbb {R}^2}\\ \left( \begin{array}{{c}} \beta _1\\ \beta _2\\ \end{array} \right) \\ \end{array} \right) +\varphi _{132}(a)\left( \begin{array}{{c}} 0_{\mathbb {R}^2}\\ \left( \begin{array}{{c}} \beta _3\\ \beta _4\\ \end{array} \right) \\ \end{array} \right) , \end{aligned}$$

5.19 $$\begin{aligned}&\Pi _h^{\mathcal {A}_{\tau _k}}\left( \begin{array}{{c}} 0_{\mathbb {R}^2}\\ Q\left( \begin{array}{{c}} \beta _3\\ \beta _4\\ \end{array} \right) \\ \end{array} \right) =(I-\Pi _c^{\mathcal {A}_{\tau _k}})\left( \begin{array}{{c}} 0_{\mathbb {R}^2}\\ Q\left( \begin{array}{{c}} \beta _3\\ \beta _4\\ \end{array} \right) \\ \end{array} \right) =\left( \begin{array}{{c}} 0_{\mathbb {R}^2}\\ \left( \begin{array}{{c}} \varphi _{141}(a)\\ \varphi _{142}(a)\\ \end{array} \right) \\ \end{array} \right) \end{aligned}$$

with $$\varphi _{141}(a)=(\mu -\varphi _{132}(a))\beta _3-\varphi _{131}(a)\beta _1\;\text{ and }\; \varphi _{142}(a)=(d-\varphi _{132}(a))\beta _4-\varphi _{131}(a)\beta _2.$$

From the above discussion we get $\sigma (\mathcal {A})=\sigma (\mathcal {A}_{\tau _k})\cup \{0\}$, and for $\lambda \in \rho (\mathcal {A})\cap \Omega =\Omega \backslash (\sigma (\mathcal {A}_{\tau _k})\cup \{0\})$ we deduce that

$$\begin{aligned} (\lambda I-\mathcal {A})^{-1}\left( \begin{array}{{c}} {{\varepsilon }}\\ \left( \begin{array}{{c}} {{\zeta }}\\ {{\delta }}\\ \end{array} \right) \\ \end{array} \right) =\left( \begin{array}{{c}} {{\frac{\varepsilon }{\lambda }}}\\ (\lambda I-\mathcal {A}_{\tau _k})^{-1}\left( \begin{array}{{c}} {{\zeta }}\\ {{\delta }}\\ \end{array} \right) \\ \end{array} \right) , \end{aligned}$$

and the eigenvalues 0 and $\pm \omega _*i$ of $\mathcal {A}$ are simple, the corresponding projectors $\Pi _0,\Pi _{\pm \omega _*i}:\mathcal {X}+i\mathcal {X}\rightarrow \mathcal {X}+i\mathcal {X}$ are denoted by

$$\begin{aligned} \Pi _0\left( \begin{array}{{c}} {{\varepsilon }}\\ {{p}}\\ \end{array} \right) =\left( \begin{array}{{c}} {{\varepsilon }}\\ {{0}}\\ \end{array} \right) ,\;\Pi _{\pm \omega _*i}\left( \begin{array}{{c}} {{\varepsilon }}\\ {{p}}\\ \end{array} \right) =\left( \begin{array}{{c}} 0\\ B_{-1,\pm \omega _*i}^{\mathcal {A}_{\tau _k}}p\\ \end{array} \right) ,\;\forall \left( \begin{array}{{c}} {{\varepsilon }}\\ {{p}}\\ \end{array} \right) \in \mathcal {X}+i\mathcal {X} \end{aligned}$$

and

$$\begin{aligned} \overline{\Pi _{\omega _*i}\left( \begin{array}{{c}} {{\varepsilon }}\\ {{p}}\\ \end{array} \right) }=\Pi _{-\omega _*i}\left( \begin{array}{{c}} \overline{\varepsilon }\\ \overline{p}\\ \end{array} \right) ,\;\forall \left( \begin{array}{{c}} {{\varepsilon }}\\ {{p}}\\ \end{array} \right) \in \mathcal {X}+i\mathcal {X}. \end{aligned}$$

In this setting, the projectors $\Pi _c,\Pi _h:\mathcal {X}\rightarrow \mathcal {X}$ are denoted by

$$\begin{aligned} \Pi _c\left( \begin{array}{{c}} {{\varepsilon }}\\ {{p}}\\ \end{array} \right) =(\Pi _0+\Pi _{\omega _*i}+\Pi _{-\omega _*i})\left( \begin{array}{{c}} {{\varepsilon }}\\ {{p}}\\ \end{array} \right) ,\forall \left( \begin{array}{{c}} {{\varepsilon }}\\ {{p}}\\ \end{array} \right) \in \mathcal {X} \end{aligned}$$

and

$$\begin{aligned} \Pi _h\left( \begin{array}{{c}} {{\varepsilon }}\\ {{p}}\\ \end{array} \right) =(I-\Pi _c)\left( \begin{array}{{c}} {{\varepsilon }}\\ {{p}}\\ \end{array} \right) ,\forall \left( \begin{array}{{c}} {{\varepsilon }}\\ {{p}}\\ \end{array} \right) \in \mathcal {X}. \end{aligned}$$

Define $\mathcal {X}_c=\Pi _c(\mathcal {X}),~\mathcal {X}_h=\Pi _h(\mathcal {X})\; \text{ and }\; \mathcal {A}_c=\mathcal {A}|_{\mathcal {X}_c},~\mathcal {A}_h=\mathcal {A}|_{\mathcal {X}_h}.$ Then we have the following decomposition

$$\begin{aligned} \mathcal {X}=\mathcal {X}_c\oplus \mathcal {X}_h. \end{aligned}$$

Consequently, we get

$$\begin{aligned} \Pi _c\left( \begin{array}{{c}} {{0_{\mathbb {R}}}}\\ {{\Theta _1}}\\ \end{array} \right)&=\left( \begin{array}{{c}} 0_{\mathbb {R}}\\ B_{-1,\omega _*i}^{\mathcal {A}_{\tau _k}}\Theta _1+B_{-1,-\omega _*i}^{\mathcal {A}_{\tau _k}}\Theta _1\\ \end{array} \right) =\Pi _{c}^{\mathcal {A}_{\tau _k}}\left( \begin{array}{{c}} {{0_{\mathbb {R}}}}\\ {{\Theta _1}}\\ \end{array} \right) ,\\ \Pi _c\left( \begin{array}{{c}} {{0_{\mathbb {R}}}}\\ {{\Theta _2}}\\ \end{array} \right)&=\left( \begin{array}{{c}} 0_{\mathbb {R}}\\ B_{-1,\omega _*i}^{\mathcal {A}_{\tau _k}}\Theta _2+B_{-1,-\omega _*i}^{\mathcal {A}_{\tau _k}}\Theta _2\\ \end{array} \right) =\Pi _{c}^{\mathcal {A}_{\tau _k}}\left( \begin{array}{{c}} {{0_{\mathbb {R}}}}\\ {{\Theta _2}}\\ \end{array} \right) . \end{aligned}$$

Moreover, we can conclude that $\Pi _h=I-\Pi _c$. Then the basis of $\mathcal {X}_c$ is

$$\begin{aligned} \nu _1&=\left( \begin{array}{{c}} 1\\ \left( \begin{array}{{c}} 0_{\mathbb {R}^2}\\ \left( \begin{array}{{c}} 0_{L^1((0,+\infty ),\mathbb {R}^2)}\\ 0_{L^1((0,+\infty ),\mathbb {R}^2)}\\ \end{array} \right) \\ \end{array} \right) \\ \end{array} \right) ,\nu _2=\left( \begin{array}{{c}} 0_{\mathbb {R}}\\ \left( \begin{array}{{c}} 0_{\mathbb {R}^2}\\ \left( \begin{array}{{c}} \beta _1+\beta _3\\ \beta _2+\beta _4\\ \end{array} \right) \\ \end{array} \right) \\ \end{array} \right) ,\\ \nu _3&=\left( \begin{array}{{c}} 0_{\mathbb {R}}\\ \left( \begin{array}{{c}} 0_{\mathbb {R}^2}\\ \left( \begin{array}{{c}} \frac{1}{i}(\beta _1-\beta _3)\\ \frac{1}{i}(\beta _2-\beta _4)\\ \end{array} \right) \\ \end{array} \right) \\ \end{array} \right) . \end{aligned}$$

For $\lambda \in i\mathbb {R}$, we can conclude that

$$\begin{aligned}&(\lambda I-\mathcal {A}_h)^{-1}\Pi _h\left( \begin{array}{{c}} {{0_{\mathbb {R}}}}\\ {{\Theta _1}}\\ \end{array} \right) =\left( \begin{array}{{c}} 0_{\mathbb {R}}\\ \left( \lambda I-\mathcal {A}_{\tau _k}^{\mathbb {C}}|_{\Pi _c^{\mathcal {A}_{\tau _k}}(X)}\right) ^{-1}\Pi _h^{\mathcal {A}_{\tau _k}} \Theta _1\\ \end{array} \right) ,\\&(\lambda I-\mathcal {A}_h)^{-1}\Pi _h\left( \begin{array}{{c}} {{0_{\mathbb {R}}}}\\ {{\Theta _2}}\\ \end{array} \right) =\left( \begin{array}{{c}} 0_{\mathbb {R}}\\ \left( \lambda I-\mathcal {A}_{\tau _k}^{\mathbb {C}}|_{\Pi _c^{\mathcal {A}_{\tau _k}}(X)}\right) ^{-1}\Pi _h^{\mathcal {A}_{\tau _k}} \Theta _2\\ \end{array} \right) . \end{aligned}$$

By calculation, we make use of the eigenfunctions of $\mathcal {A}$ in $\mathcal {X}_c$ and

$$\begin{aligned} \overline{\nu }_1=\left( \begin{array}{{c}} 1\\ \left( \begin{array}{{c}} 0_{\mathbb {R}^2}\\ \left( \begin{array}{{c}} 0_{L^1((0,+\infty ),\mathbb {R}^2)}\\ 0_{L^1((0,+\infty ),\mathbb {R}^2)}\\ \end{array} \right) \\ \end{array} \right) \\ \end{array} \right) ,\overline{\nu }_2=\left( \begin{array}{{c}} 0_{\mathbb {R}}\\ \left( \begin{array}{{c}} 0_{\mathbb {R}^2}\\ \left( \begin{array}{{c}} \beta _1\\ \beta _2\\ \end{array} \right) \\ \end{array} \right) \\ \end{array} \right) ,\overline{\nu }_3=\left( \begin{array}{{c}} 0_{\mathbb {R}}\\ \left( \begin{array}{{c}} 0_{\mathbb {R}^2}\\ \left( \begin{array}{{c}} \beta _3\\ \beta _4\\ \end{array} \right) \\ \end{array} \right) \\ \end{array} \right) . \end{aligned}$$

Then we get that

$$\begin{aligned} \mathcal {A}\overline{\nu }_1=0,\;\mathcal {A}\overline{\nu }_2=\omega _*i\overline{\nu }_2, \;\mathcal {A}\overline{\nu }_3=-\omega _*i\overline{\nu }_3, \end{aligned}$$

and hence, the matrix of $\mathcal {A}_c$ in the basis $\{\overline{\nu }_1,\overline{\nu }_2,\overline{\nu }_3\}$ of $\mathcal {X}_c$ is given by

$$\begin{aligned} \mathcal {A}(\overline{\nu }_1,\overline{\nu }_2,\overline{\nu }_3)=(\overline{\nu }_1,\overline{\nu }_2,\overline{\nu }_3) \left( \begin{array}{{ccc}} 0&{}0&{}0\\ 0&{}\omega _*i&{}0\\ 0&{}0&{}-\omega _*i\\ \end{array} \right) . \end{aligned}$$

### Computation of the Taylor expansion

In this subsection, we compute the Taylor expansion of reduced system of system (1.4) on the center manifold. Firstly, we put

$$\begin{aligned} \kappa =\left( \begin{array}{{c}} \tilde{\tau }\\ \hat{p}\\ \end{array} \right) =\left( \begin{array}{{c}} \tilde{\tau }\\ \left( \begin{array}{{c}} 0_{\mathbb {R}^2}\\ p\\ \end{array} \right) \\ \end{array} \right) \in \overline{D(\mathcal {A})}\;\textrm{and}\; p=\left( \begin{array}{{c}} \varphi ^1\\ \varphi ^2\\ \end{array} \right) , \end{aligned}$$

$$ \Pi _c^{\mathcal {A}_{\tau _k}}\hat{p}=\hat{p}_c\;\text {and}\;\kappa _c =\Pi _c\kappa =\left( \begin{array}{{c}} \tilde{\tau }\\ \Pi _c^{\mathcal {A}_{\tau _k}}\hat{p}\\ \end{array} \right) =\left( \begin{array}{{c}} \tilde{\tau }\\ \hat{p}_c\\ \end{array} \right) , \Pi _h^{\mathcal {A}_{\tau _k}}\hat{p}=\hat{p}_h\;\textrm{and}\;\kappa _h=\Pi _h\kappa =(I-\Pi _c)\kappa =\left( \begin{array}{{c}} 0\\ \Pi _h^{\mathcal {A}_{\tau _k}}\hat{p}\\ \end{array} \right) =\left( \begin{array}{{c}} 0\\ \hat{p}_h\\ \end{array} \right) . $$ Notice that $$ \left\{ \left( \begin{array}{{c}} 0_{\mathbb {R}^2}\\ \left( \begin{array}{{c}} \beta _1\\ \beta _2\\ \end{array} \right) \\ \end{array} \right) ,\left( \begin{array}{{c}} 0_{\mathbb {R}^2}\\ \left( \begin{array}{{c}} \beta _3\\ \beta _4\\ \end{array} \right) \\ \end{array} \right) \right\} $$ is the basis of $X_c$, then there exists a unique pair of real numbers $\varsigma _1,\varsigma _2\in \mathbb {R}$ such that

$$\begin{aligned} \hat{p}_c=\left( \begin{array}{{c}} 0_{\mathbb {R}^2}\\ \varsigma _1\left( \begin{array}{{c}} \beta _1\\ \beta _2\\ \end{array} \right) +\varsigma _2\left( \begin{array}{{c}} \beta _3\\ \beta _4\\ \end{array} \right) \\ \end{array} \right) . \end{aligned}$$

Here we recall that the nonlinear maps $\mathcal {H}$ and $\mathcal {K}$ are defined in (5.5) and (5.6), respectively. Then, for any $$ \kappa _1=\left( \begin{array}{{c}} \tilde{\tau }_1\\ \hat{p}_1\\ \end{array} \right) ,\;\kappa _2=\left( \begin{array}{{c}} \tilde{\tau }_2\\ \hat{p}_2\\ \end{array} \right) ,\;\kappa _3=\left( \begin{array}{{c}} \tilde{\tau }_3\\ \hat{p}_3\\ \end{array} \right) \in \overline{D(\mathcal {A})}\;\text{ with }\;\hat{p}_i=\left( \begin{array}{{c}} 0_{\mathbb {R}^2}\\ \left( \begin{array}{{c}} \varphi _i^1\\ \varphi _i^2\\ \end{array} \right) \\ \end{array} \right) (i=1,2,3), $$ we have

5.20 $$\begin{aligned}&D^2{{\mathcal {H}(0_\mathcal {X})}}(\kappa _1,\kappa _2)=D^2{{\mathcal {H}(0_\mathcal {X})}}\left( \left( \begin{array}{{c}} \tilde{\tau }_1\\ \hat{p}_1\\ \end{array} \right) ,\left( \begin{array}{{c}} \tilde{\tau }_2\\ \hat{p}_2\\ \end{array} \right) \right) \nonumber \\&\quad =\tau _kD^2F(\overline{p}_{+})(\hat{p}_1,\hat{p}_2)+\tilde{\tau }_2DF(\overline{p}_{+})(\hat{p}_1)+\tilde{\tau }_1DF(\overline{p}_{+})(\hat{p}_2)\nonumber \\&\qquad +\tau _k\tilde{\tau }_2D^2F(\overline{p}_{+})\left( \hat{p}_1,\left( \frac{\text{ d } {\overline{p}_{+}}_{\tilde{\tau }(t)+\tau _k}}{\text{ d } \tilde{\tau }}\right) \Bigg |_{\tilde{\tau }=0}\right) \nonumber \\&\qquad +\tau _k\tilde{\tau }_1D^2F(\overline{p}_{+})\left( \hat{p}_2,\left( \frac{\text{ d } {\overline{p}_{+}}_{\tilde{\tau }(t)+\tau _k}}{\text{ d } \tilde{\tau }}\right) \Bigg |_{\tilde{\tau }=0}\right) \nonumber \\&\qquad -\tilde{\tau }_1{{D\mathcal {K}(0_X)}}(\hat{p}_2)-\tilde{\tau }_2{{D\mathcal {K}(0_X)}}(\hat{p}_1) \end{aligned}$$

with $${{D\mathcal {K}(0_X)(\hat{p}_i)=D\mathcal {K}(0_X)}}\left( \begin{array}{{c}} 0_{\mathbb {R}^2}\\ \left( \begin{array}{{c}} \varphi _i^1\\ \varphi _i^2\\ \end{array} \right) \\ \end{array} \right) =\left( \begin{array}{{c}} 0_{\mathbb {R}^2}\\ Q\left( \begin{array}{{c}} \varphi _i^1\\ \varphi _i^2\\ \end{array} \right) \\ \end{array} \right) (i=1,2) $$ and

$$\begin{aligned} D^2F({\overline{p}_{+}})(\hat{p}_1,\hat{p}_2)=D^2F({\overline{p}_{+}})\left( \left( \begin{array}{{c}} 0_{\mathbb {R}^2}\\ \left( \begin{array}{{c}} \varphi _1^1\\ \varphi _1^2\\ \end{array} \right) \\ \end{array} \right) ,\left( \begin{array}{{c}} 0_{\mathbb {R}^2}\\ \left( \begin{array}{{c}} \varphi _2^1\\ \varphi _2^2\\ \end{array} \right) \\ \end{array} \right) \right) =\left( \begin{array}{{c}} \left( \begin{array}{{c}} \varphi _{1111}\\ \varphi _{1112}\\ \end{array} \right) \\ 0_{L^1((0,+\infty ),\mathbb {R}^2)}\\ \end{array} \right) , \end{aligned}$$

where

$$\begin{aligned} \varphi _{1111}&=\frac{\mu (2-b\eta )P_1}{b^2\eta ^2\nu ^2m_1}\left( \int _{0}^{+\infty }\varphi _1^1(a)\text{ d }a\int _{0}^{+\infty }\varphi _2^2(a)\text{ d }a\right. \\&\quad \left. +\int _{0}^{+\infty }\varphi _1^2(a)\text{ d }a\int _{0}^{+\infty }\varphi _2^1(a)\text{ d }a\right) \\&\quad -\frac{s}{b^2\eta \nu m_1}\left( \int _{0}^{+\infty }\varphi _1^1(a)\text{ d }a\int _{0}^{+\infty }f(a)\varphi _2^1(a)\text{ d }a\right. \\&\quad \left. +\int _{0}^{+\infty }f(a)\varphi _1^1(a)\text{ d }a\int _{0}^{+\infty }\varphi _2^1(a)\text{ d }a\right) \\&\quad +\frac{b\eta -1}{b^2\eta \nu }\left( \int _{0}^{+\infty }f(a)\varphi _1^1(a)\text{ d }a\int _{0}^{+\infty }\varphi _2^2(a)\text{ d }a\right. \\&\quad \left. +\int _{0}^{+\infty }\varphi _1^2(a)\text{ d }a\int _{0}^{+\infty }f(a)\varphi _2^1(a)\text{ d }a\right) \\&\quad +\frac{2s\mu P_1}{(b\eta \nu m_1)^2} \left( \int _{0}^{+\infty }\varphi _1^1(a)\text{ d }a\int _{0}^{+\infty }\varphi _2^1(a)\text{ d }a\right) \\&\quad -\frac{2\mu (b\eta -1)P_1}{sb^2\eta ^2\nu ^2} \left( \int _{0}^{+\infty }\varphi _1^2(a)\text{ d }a\int _{0}^{+\infty }\varphi _2^2(a)\text{ d }a\right) ,\\ \varphi _{1112}&=\frac{s}{b^2\eta ^2\nu m_1}\left( \int _{0}^{+\infty }\varphi _1^1(a)\text{ d }a\int _{0}^{+\infty }\beta (a)\varphi _2^1(a)\text{ d }a\right. \\&\quad \left. +\int _{0}^{+\infty }\beta (a)\varphi _1^1(a)\text{ d }a\int _{0}^{+\infty }\varphi _2^1(a)\text{ d }a\right) \\&\quad -\frac{\mu (2-b\eta )P_1}{b^3\eta ^3\nu ^2m_1} \left( \int _{0}^{+\infty }\varphi _1^1(a)\text{ d }a\int _{0}^{+\infty }\varphi _2^2(a)\text{ d }a\right. \\&\quad \left. +\int _{0}^{+\infty }\varphi _1^2(a)\text{ d }a\int _{0}^{+\infty }\varphi _2^1(a)\text{ d }a\right) \\&\quad -\frac{b\eta -1}{b^2\eta ^2\nu }\left( \int _{0}^{+\infty }\beta (a)\varphi _1^1(a)\text{ d }a\int _{0}^{+\infty }\varphi _2^2(a)\text{ d }a\right. \\&\quad \left. +\int _{0}^{+\infty }\varphi _1^2(a)\text{ d }a\int _{0}^{+\infty }\beta (a)\varphi _2^1(a)\text{ d }a\right) \\&\quad +\frac{2[K \mu (b\eta -1)P_1- s r b^3\eta ^3\nu ^2m_1]}{Ksb^3\eta ^3\nu ^2m_1}\\&\quad \left( \int _{0}^{+\infty }\varphi _1^2(a)\text{ d }a\int _{0}^{+\infty }\varphi _2^2(a)\text{ d }a\right) \\&\quad -\frac{2\mu sP_1}{b^3\eta ^3\nu ^2m_1^2}\left( \int _{0}^{+\infty }\varphi _1^1(a)\text{ d }a\int _{0}^{+\infty }\varphi _2^1(a)\text{ d }a\right) . \end{aligned}$$

Consequently, we get

$$\begin{aligned} \frac{1}{2!}D^2{{\mathcal {H}(0_\mathcal {X})}}(\kappa )^2&=\frac{1}{2!}D^2{{\mathcal {H}(0_\mathcal {X})}}\left( \begin{array}{{c}} \tilde{\tau }\\ \left( \begin{array}{{c}} 0_{\mathbb {R}^2}\\ \left( \begin{array}{{c}} \overline{\varphi }^1\\ \overline{\varphi }^2\\ \end{array} \right) \\ \end{array} \right) \\ \end{array} \right) ^2 =\tilde{\tau }DF({\overline{p}_{+}})\left( \begin{array}{{c}} 0_{\mathbb {R}^2}\\ \left( \begin{array}{{c}} \overline{\varphi }^1\\ \overline{\varphi }^2\\ \end{array} \right) \\ \end{array} \right) \\&\quad +\frac{1}{2}\tau _kD^2F({\overline{p}_{+}})\left( \begin{array}{{c}} 0_{\mathbb {R}^2}\\ \left( \begin{array}{{c}} \overline{\varphi }^1\\ \overline{\varphi }^2\\ \end{array} \right) \\ \end{array} \right) ^2 +\tilde{\tau }\tau _kD^2F({\overline{p}_{+}})\left( \left( \begin{array}{{c}} 0_{\mathbb {R}^2}\\ \left( \begin{array}{{c}} \overline{\varphi }^1\\ \overline{\varphi }^2\\ \end{array} \right) \\ \end{array} \right) ,\right. \\&\quad \left. \left( \begin{array}{{c}} 0_{\mathbb {R}^2}\\ \left( \frac{\textrm{d} {\overline{p}_{+}}_{\tilde{\tau }(t)+\tau _k}}{\textrm{d} \tilde{\tau }}\right) \Big |_{\tilde{\tau }=0}\\ \end{array} \right) \right) \\&\quad -\tilde{\tau }D{{\mathcal {K}(0_X)}}\left( \begin{array}{{c}} 0_{\mathbb {R}^2}\\ \left( \begin{array}{{c}} \overline{\varphi }^1\\ \overline{\varphi }^2\\ \end{array} \right) \\ \end{array} \right) =\left( \begin{array}{{c}} \left( \begin{array}{{c}} \widehat{\overline{\varphi }}_1(\tau _k,\tilde{\tau })\\ \widehat{\overline{\varphi }}_2(\tau _k,\tilde{\tau })\\ \end{array} \right) \\ -\tilde{\tau }Q\left( \begin{array}{{c}} \overline{\varphi }^1\\ \overline{\varphi }^2\\ \end{array} \right) \\ \end{array} \right) , \end{aligned}$$

where the forms of $\widehat{\overline{\varphi }}_1(\tau _k,\tilde{\tau })$ and $\widehat{\overline{\varphi }}_2(\tau _k,\tilde{\tau })$ are given in Appendix A.1. By projecting on $X_c$, we have

$$\begin{aligned}&\frac{1}{2!}\Pi _c^{\mathcal {A}_{\tau _k}}D^2{{\mathcal {H}(0_\mathcal {X})}}\left( \begin{array}{{c}} \tilde{\tau }\\ \left( \begin{array}{{c}} 0_{\mathbb {R}^2}\\ p\\ \end{array} \right) \\ \end{array} \right) ^2\\&\quad = \widehat{\overline{\varphi }}_1(\tau _k,\tilde{\tau })\Pi _c^{\mathcal {A}_{\tau _k}}\Theta _1 +\widehat{\overline{\varphi }}_2(\tau _k,\tilde{\tau })\Pi _c^{\mathcal {A}_{\tau _k}}\Theta _2 +\Pi _c^{\mathcal {A}_{\tau _k}}\left( \begin{array}{{c}} 0_{\mathbb {R}^2}\\ -\tilde{\tau }Q\left( \begin{array}{{c}} \overline{\varphi }^1\\ \overline{\varphi }^2\\ \end{array} \right) \\ \end{array} \right) \\&\qquad +\widehat{\overline{\varphi }}_1(\tau _k,\tilde{\tau })\left( \begin{array}{{c}} 0_{\mathbb {R}^2}\\ \varphi _1(a)\\ \end{array} \right) +\widehat{\overline{\varphi }}_2(\tau _k,\tilde{\tau })\left( \begin{array}{{c}} 0_{\mathbb {R}^2}\\ \varphi _2(a)\\ \end{array} \right) -\tilde{\tau }\Pi _c^{\mathcal {A}_{\tau _k}}\left( \begin{array}{{c}} 0_{\mathbb {R}^2}\\ Q\left( \begin{array}{{c}} \overline{\varphi }^1\\ \overline{\varphi }^2\\ \end{array} \right) \\ \end{array} \right) . \end{aligned}$$

Now we compute $\frac{1}{2!}D^2{{\mathcal {H}(0_\mathcal {X})}}(\kappa _c)^2,~\frac{1}{2!}\Pi _cD^2{{\mathcal {P}(0_\mathcal {X})}} (\kappa _c)^2,~\frac{1}{2!}\Pi _hD^2{{\mathcal {P}(0_\mathcal {X})}}(\kappa _c)^2$ and $\frac{1}{3!}D^3{{\mathcal {H}(0_\mathcal {X})}}(\kappa _c)^2$ expressed in terms of the basis ${{\{\overline{\nu }_1,\overline{\nu }_2,\overline{\nu }_3\}}}$, where $\kappa _c=\tilde{\tau }\overline{\nu }_1+\varsigma _1\overline{\nu }_2+\varsigma _2\overline{\nu }_3$. Firstly, we have

$$\begin{aligned} \frac{1}{2!}D^2{{\mathcal {H}(0_\mathcal {X})}}(\kappa _c)^2&=\frac{1}{2!}D^2{{\mathcal {H}(0_\mathcal {X})}}\left( \begin{array}{{c}} \tilde{\tau }\\ \left( \begin{array}{{c}} 0_{\mathbb {R}^2}\\ \varsigma _1\left( \begin{array}{{c}} \beta _1\\ \beta _2\\ \end{array} \right) +\varsigma _2\left( \begin{array}{{c}} \beta _3\\ \beta _4\\ \end{array} \right) \\ \end{array} \right) \\ \end{array} \right) ^2\\&=\left( \begin{array}{{c}} \left( \begin{array}{{c}} \widehat{\overline{\overline{\varphi }}}_1(\tau _k,\tilde{\tau })\\ \widehat{\overline{\overline{\varphi }}}_2(\tau _k,\tilde{\tau })\\ \end{array} \right) \\ -\tilde{\tau }Q\left( \begin{array}{{c}} \overline{\overline{\varphi }}^1\\ \overline{\overline{\varphi }}^2\\ \end{array} \right) \\ \end{array} \right) \end{aligned}$$

with $\overline{\overline{\varphi }}^1=\varsigma _1\beta _1+\varsigma _2\beta _3$ and $\overline{\overline{\varphi }}^2=\varsigma _1\beta _2+\varsigma _2\beta _4$, where the forms of $\widehat{\overline{\overline{\varphi }}}_1(\tau _k,\tilde{\tau })$ and $\widehat{\overline{\overline{\varphi }}}_2(\tau _k,\tilde{\tau })$ are given in Appendix A.1. Then, according to (5.9), (5.10), (5.16) and (5.18), we get

5.21 $$\begin{aligned}&\frac{1}{2!}\Pi _cD^2{{\mathcal {P}(0_\mathcal {X})}}(\kappa _c)^2\nonumber \\&\quad =\left( \begin{array}{{c}} 0_{\mathbb {R}}\\ \frac{1}{2!}\Pi _c^{\mathcal {A}_{\tau _k}}D^2{{\mathcal {H}(0_\mathcal {X})}}\left( \begin{array}{{c}} \tilde{\tau }\\ \hat{p}_c\\ \end{array} \right) ^2\\ \end{array} \right) =\left( \begin{array}{{c}} 0_{\mathbb {R}}\\ \widehat{\overline{\overline{\varphi }}}_1(\tau _k,\tilde{\tau })\Pi _c^{\mathcal {A}_{\tau _k}} \Theta _1\\ \end{array} \right) \nonumber \\&\qquad +\left( \begin{array}{{c}} 0_{\mathbb {R}}\\ \widehat{\overline{\overline{\varphi }}}_2(\tau _k,\tilde{\tau })\Pi _c^{\mathcal {A}_{\tau _k}} \Theta _2\\ \end{array} \right) \nonumber \\&\qquad -\left( \begin{array}{{c}} 0_{\mathbb {R}}\\ \varsigma _1\tilde{\tau }\Pi _c^{\mathcal {A}_{\tau _k}}\left( \begin{array}{{c}} 0_{\mathbb {R}^2}\\ Q\left( \begin{array}{{c}} \beta _1\\ \beta _2\\ \end{array} \right) \\ \end{array} \right) \\ \end{array} \right) -\left( \begin{array}{{c}} 0_{\mathbb {R}}\\ \varsigma _2\tilde{\tau }\Pi _c^{\mathcal {A}_{\tau _k}}\left( \begin{array}{{c}} 0_{\mathbb {R}^2}\\ Q\left( \begin{array}{{c}} \beta _3\\ \beta _4\\ \end{array} \right) \\ \end{array} \right) \\ \end{array} \right) \nonumber \\&\quad =\left( \begin{array}{{c}} 0_{\mathbb {R}}\\ \left( \begin{array}{{c}} 0_{\mathbb {R}^2}\\ \widetilde{\varphi }_{1}(\tau _k,\tilde{\tau })\left( \begin{array}{{c}} \beta _1\hbar _{\lambda _0=\omega _*i,11}\\ \beta _2\hbar _{\lambda _0=\omega _*i,21}\\ \end{array} \right) +\widetilde{\varphi }_{2}(\tau _k,\tilde{\tau })\left( \begin{array}{{c}} \beta _3\hbar _{\lambda _0=-\omega _*i,12}\\ \beta _4\hbar _{\lambda _0=-\omega _*i,22}\\ \end{array} \right) \\ \end{array} \right) \\ \end{array} \right) \end{aligned}$$

with

$$\begin{aligned} \widetilde{\varphi }_{1}(\tau _k,\tilde{\tau })&=\left( \frac{\text{ d }~\text{ det }(\Delta (\omega _*i))}{\text{ d }\lambda }\right) ^{-1} \left( \widehat{\overline{\overline{\varphi }}}_1(\tau _k,\tilde{\tau })+ \widehat{\overline{\overline{\varphi }}}_2(\tau _k,\tilde{\tau })\right) \\&\quad -\tilde{\tau }\left( \varsigma _1\varphi _{111}(a) -\varsigma _2\varphi _{131}(a)\right) ,\\ \widetilde{\varphi }_{2}(\tau _k,\tilde{\tau })&=\left( \frac{\text{ d }~\text{ det }(\Delta (-\omega _*i))}{\text{ d }\lambda }\right) ^{-1} \left( \widehat{\overline{\overline{\varphi }}}_1(\tau _k,\tilde{\tau })+ \widehat{\overline{\overline{\varphi }}}_2(\tau _k,\tilde{\tau })\right) \\&\quad -\tilde{\tau }\left( \varsigma _1\varphi _{112}(a) -\varsigma _2\varphi _{132}(a)\right) . \end{aligned}$$

In addition, according to (5.11), (5.12), (5.17) and (5.19), we get

5.22 $$\begin{aligned} \frac{1}{2!}\Pi _hD^2{{\mathcal {P}(0_\mathcal {X})}}(\kappa _c)^2&=\frac{1}{2!}(I-\Pi _c)D^2{{\mathcal {P}(0_\mathcal {X})}}(\kappa _c)^2\nonumber \\&=\left( \begin{array}{{c}} 0_{\mathbb {R}}\\ \frac{1}{2!}(I-\Pi _c^{\mathcal {A}_{\tau _k}})D^2{{\mathcal {H}(0_\mathcal {X})}}\left( \begin{array}{{c}} \tilde{\tau }\\ \hat{p}_c\\ \end{array} \right) ^2\\ \end{array} \right) \nonumber \\&=\left( \begin{array}{{c}} 0_{\mathbb {R}}\\ \left( \begin{array}{{c}} \widetilde{\widetilde{\varphi }}(\tau _k,\tilde{\tau })\\ \left( \begin{array}{{c}} \widehat{\overline{\overline{\varphi }}}_1(\tau _k,\tilde{\tau })\\ \widehat{\overline{\overline{\varphi }}}_2(\tau _k,\tilde{\tau })\\ \end{array} \right) \\ \end{array} \right) \\ \end{array} \right) , \end{aligned}$$

where

$$\begin{aligned} \widetilde{\widetilde{\varphi }}(\tau _k,\tilde{\tau })&=-Q \left[ \varsigma _1\left( \begin{array}{{c}} \beta _1\hbar _{\lambda _0=\omega _*i,11}\\ \beta _2\hbar _{\lambda _0=\omega _*i,21}\\ \end{array} \right) +\varsigma _2\left( \begin{array}{{c}} \beta _3\hbar _{\lambda _0=-\omega _*i,12}\\ \beta _4\hbar _{\lambda _0=-\omega _*i,22}\\ \end{array} \right) \right] \\&\quad +\widetilde{\varphi }_3(\tau _k,\tilde{\tau })\left( \begin{array}{{c}} \beta _1\hbar _{\lambda _0=\omega _*i,11}\\ \beta _2\hbar _{\lambda _0=\omega _*i,21}\\ \end{array} \right) +\widetilde{\varphi }_4(\tau _k,\tilde{\tau })\left( \begin{array}{{c}} \beta _3\hbar _{\lambda _0=-\omega _*i,12}\\ \beta _4\hbar _{\lambda _0=-\omega _*i,22}\\ \end{array} \right) \end{aligned}$$

with

$$\begin{aligned} \widetilde{\varphi }_{3}(\tau _k,\tilde{\tau })&=-\left( \frac{\text{ d }~\text{ det }(\Delta (\omega _*i))}{\text{ d }\lambda }\right) ^{-1} \left( \widehat{\overline{\overline{\varphi }}}_1(\tau _k,\tilde{\tau })+ \widehat{\overline{\overline{\varphi }}}_2(\tau _k,\tilde{\tau })\right) \\&\quad +\tilde{\tau }\left( \varsigma _1\varphi _{111}(a) +\varsigma _2\varphi _{131}(a)\right) ,\\ \widetilde{\varphi }_{4}(\tau _k,\tilde{\tau })&=-\left( \frac{\text{ d }~\text{ det }(\Delta (-\omega _*i))}{\text{ d }\lambda }\right) ^{-1} \left( \widehat{\overline{\overline{\varphi }}}_1(\tau _k,\tilde{\tau })+ \widehat{\overline{\overline{\varphi }}}_2(\tau _k,\tilde{\tau })\right) \\&\quad +\tilde{\tau }\left( \varsigma _1\varphi _{112}(a) +\varsigma _2\varphi _{132}(a)\right) . \end{aligned}$$

For the calculation of ${{D^3\mathcal {H}(0_\mathcal {X})(\kappa _1,\kappa _2,\kappa _3)}}$, we have

$$\begin{aligned}&{{D^3\mathcal {H}(0_\mathcal {X})(\kappa _1,\kappa _2,\kappa _3)}}\\&\quad ={{D^3\mathcal {H}(0_\mathcal {X})}}\left( \left( \begin{array}{{c}} \tilde{\tau }_1\\ \hat{p}_1\\ \end{array} \right) ,\left( \begin{array}{{c}} \tilde{\tau }_2\\ \hat{p}_2\\ \end{array} \right) ,\left( \begin{array}{{c}} \tilde{\tau }_3\\ \hat{p}_3\\ \end{array} \right) \right) \\&\quad =\tilde{\tau }_1D^2F({\overline{p}_{+}})(\hat{p}_2,\hat{p}_3)+\tilde{\tau }_2D^2F({\overline{p}_{+}})(\hat{p}_1,\hat{p}_3)+ \tilde{\tau }_3D^2F({\overline{p}_{+}})(\hat{p}_1,\hat{p}_2)\\&\qquad +2\tilde{\tau }_2\tilde{\tau }_3D^2F({\overline{p}_{+}})\left( \hat{p}_1,\left( \frac{\text{ d } {\overline{p}_{+}}_{\tilde{\tau }(t)+\tau _k}}{\text{ d } \tilde{\tau }}\right) \Bigg |_{\tilde{\tau }=0}\right) \\&\qquad +2\tilde{\tau }_1\tilde{\tau }_3D^2F({\overline{p}_{+}})\left( \hat{p}_2,\left( \frac{\text{ d } {\overline{p}_{+}}_{\tilde{\tau }(t)+\tau _k}}{\text{ d } \tilde{\tau }}\right) \Bigg |_{\tilde{\tau }=0}\right) \\&\qquad +2\tilde{\tau }_1\tilde{\tau }_2D^2F({\overline{p}_{+}})\left( \hat{p}_3,\left( \frac{\text{ d } {\overline{p}_{+}}_{\tilde{\tau }(t)+\tau _k}}{\text{ d } \tilde{\tau }}\right) \Bigg |_{\tilde{\tau }=0}\right) \\&\qquad +\tilde{\tau }_2\tilde{\tau }_3\tau _kD^2F({\overline{p}_{+}})\left( \hat{p}_1,\left( \frac{\text{ d}^2 {\overline{p}_{+}}_{\tilde{\tau }(t)+\tau _k}}{\text{ d } \tilde{\tau }^2}\right) \Bigg |_{\tilde{\tau }=0}\right) \\&\qquad +\tilde{\tau }_1\tilde{\tau }_3\tau _kD^2F({\overline{p}_{+}})\left( \hat{p}_2,\left( \frac{\text{ d}^2 {\overline{p}_{+}}_{\tilde{\tau }(t)+\tau _k}}{\text{ d } \tilde{\tau }^2}\right) \Bigg |_{\tilde{\tau }=0}\right) \\&\qquad +\tilde{\tau }_1\tilde{\tau }_2\tau _kD^2F({\overline{p}_{+}})\left( \hat{p}_3,\left( \frac{\text{ d}^2 {\overline{p}_{+}}_{\tilde{\tau }(t)+\tau _k}}{\text{ d } \tilde{\tau }^2}\right) \Bigg |_{\tilde{\tau }=0}\right) \\&\qquad +\tau _kD^3F({\overline{p}_{+}})(\hat{p}_1,\hat{p}_2,\hat{p}_3)+\tilde{\tau }_3\tau _kD^3F({\overline{p}_{+}})\left( \hat{p}_1,\hat{p}_2,\left( \frac{\text{ d } {\overline{p}_{+}}_{\tilde{\tau }(t)+\tau _k}}{\text{ d } \tilde{\tau }}\right) \Bigg |_{\tilde{\tau }=0}\right) \\&\qquad +\tilde{\tau }_2\tau _kD^3F({\overline{p}_{+}})\left( \hat{p}_1,\hat{p}_3,\left( \frac{\text{ d } {\overline{p}_{+}}_{\tilde{\tau }(t)+\tau _k}}{\text{ d } \tilde{\tau }}\right) \Bigg |_{\tilde{\tau }=0}\right) \\&\qquad +\tilde{\tau }_1\tau _kD^3F({\overline{p}_{+}})\left( \hat{p}_2,\hat{p}_3,\left( \frac{\text{ d } {\overline{p}_{+}}_{\tilde{\tau }(t)+\tau _k}}{\text{ d } \tilde{\tau }}\right) \Bigg |_{\tilde{\tau }=0}\right) \\&\qquad +\tilde{\tau }_2\tilde{\tau }_3\tau _kD^3F({\overline{p}_{+}})\left( \hat{p}_1,\left( \frac{\text{ d } {\overline{p}_{+}}_{\tilde{\tau }(t)+\tau _k}}{\text{ d } \tilde{\tau }}\right) \Bigg |_{\tilde{\tau }=0},\left( \frac{\text{ d } {\overline{p}_{+}}_{\tilde{\tau }(t)+\tau _k}}{\text{ d } \tilde{\tau }}\right) \Bigg |_{\tilde{\tau }=0}\right) \\&\qquad +\tilde{\tau }_1\tilde{\tau }_3\tau _kD^3F({\overline{p}_{+}})\left( \hat{p}_2,\left( \frac{\text{ d } {\overline{p}_{+}}_{\tilde{\tau }(t)+\tau _k}}{\text{ d } \tilde{\tau }}\right) \Bigg |_{\tilde{\tau }=0},\left( \frac{\text{ d } {\overline{p}_{+}}_{\tilde{\tau }(t)+\tau _k}}{\text{ d } \tilde{\tau }}\right) \Bigg |_{\tilde{\tau }=0}\right) \\&\qquad +\tilde{\tau }_1\tilde{\tau }_2\tau _kD^3F({\overline{p}_{+}})\left( \hat{p}_3,\left( \frac{\text{ d } {\overline{p}_{+}}_{\tilde{\tau }(t)+\tau _k}}{\text{ d } \tilde{\tau }}\right) \Bigg |_{\tilde{\tau }=0},\left( \frac{\text{ d } {\overline{p}_{+}}_{\tilde{\tau }(t)+\tau _k}}{\text{ d } \tilde{\tau }}\right) \Bigg |_{\tilde{\tau }=0}\right) \\ \end{aligned}$$

with

$$\begin{aligned} D^3F({\overline{p}_{+}})\left( \left( \begin{array}{{c}} 0_{\mathbb {R}^2}\\ \left( \begin{array}{{c}} \varphi _1^1\\ \varphi _1^2\\ \end{array} \right) \\ \end{array} \right) ,\left( \begin{array}{{c}} 0_{\mathbb {R}^2}\\ \left( \begin{array}{{c}} \varphi _2^1\\ \varphi _2^2\\ \end{array} \right) \\ \end{array} \right) ,\left( \begin{array}{{c}} 0_{\mathbb {R}^2}\\ \left( \begin{array}{{c}} \varphi _3^1\\ \varphi _3^2\\ \end{array} \right) \\ \end{array} \right) \right) =\left( \begin{array}{{c}} \left( \begin{array}{{c}} \varphi _{2211}\\ \varphi _{2212}\\ \end{array} \right) \\ 0_{L^1((0,+\infty ),\mathbb {R}^2)}\\ \end{array} \right) , \end{aligned}$$

where the forms of $\varphi _{2211}$ and $\varphi _{2212}$ are given in Appendix A.1.

Similarly, we obtain

$$\begin{aligned} \frac{1}{3!}D^3{{\mathcal {H}(0_\mathcal {X})}}(\kappa _c)^3=\frac{1}{3!}D^3{{\mathcal {H}(0_\mathcal {X})}} \left( \begin{array}{{c}} \tilde{\tau }\\ \hat{p}_c\\ \end{array} \right) ^3= \left( \begin{array}{{c}} \left( \begin{array}{{c}} \widehat{\overline{\varphi }}_3(\tau _k,\tilde{\tau })\\ \widehat{\overline{\varphi }}_4(\tau _k,\tilde{\tau })\\ \end{array} \right) \\ 0_{L^1((0,+\infty ),\mathbb {R}^2)}\\ \end{array} \right) , \end{aligned}$$

where the forms of $\widehat{\overline{\varphi }}_3(\tau _k,\tilde{\tau })$ and $\widehat{\overline{\varphi }}_4(\tau _k,\tilde{\tau })$ are given in Appendix A.1. Then we can get

5.23 $$\begin{aligned} \frac{1}{3!}\Pi _cD^3{{\mathcal {P}(0_\mathcal {X})}}(\kappa _c)^3&=\left( \begin{array}{{c}} 0_{\mathbb {R}}\\ \frac{1}{3!}\Pi _c^{\mathcal {A}_{\tau _k}}D^3{{\mathcal {H}(0_\mathcal {X})}}\left( \begin{array}{{c}} \tilde{\tau }\\ \hat{p}_c\\ \end{array} \right) ^3\\ \end{array} \right) \nonumber \\&=\left( \begin{array}{{c}} 0_{\mathbb {R}}\\ \widehat{\overline{\varphi }}_3(\tau _k,\tilde{\tau })\left( \begin{array}{{c}} 0_{\mathbb {R}^2}\\ \varphi _1(a)\\ \end{array} \right) \\ \end{array} \right) +\left( \begin{array}{{c}} 0_{\mathbb {R}}\\ \widehat{\overline{\varphi }}_4(\tau _k,\tilde{\tau })\left( \begin{array}{{c}} 0_{\mathbb {R}^2}\\ \varphi _2(a)\\ \end{array} \right) \\ \end{array} \right) . \end{aligned}$$

### Computation of $\mathcal {A}_2$

In order to calculate the normal form of the reduced system of system (5.7) up to the third order terms, the main point is to compute $\mathcal {U}_2\in J^2(\mathcal {X},D(\mathcal {A}))$, where $\mathcal {U}_2$ can be defined in Eq. (5.33) and $J^2(\mathcal {X},D(\mathcal {A}))$ is the linear space of homogeneous polynomials of degree 2. Firstly, we consider a linear operator $\mathcal {A}_2\in \mathcal {A}_s\left( \mathcal {X}_c^2,\mathcal {X}_h\cap D(\mathcal {A})\right)$ that satisfies the following equation

5.24 $$\begin{aligned} {{\frac{\text{ d }}{\text{ d } t}\left[ \mathcal {A}_2\left( e^{\mathcal {A}_ct}\kappa _1,e^{\mathcal {A}_ct}\kappa _2\right) \right] (0)=\mathcal {A}_h\mathcal {A}_2(\kappa _1,\kappa _2) +\frac{1}{2!}\Pi _hD^2\mathcal {P}(0_\mathcal {X})(\kappa _1,\kappa _2).}} \end{aligned}$$

for each $(\kappa _1,\kappa _2)\in \mathcal {X}_c^2$. Note that

$$\begin{aligned} {{\frac{\text{ d }}{\text{ d } t}\left[ \mathcal {A}_2\left( e^{\mathcal {A}_ct}\kappa _1,e^{\mathcal {A}_ct}\kappa _2\right) \right] (0)=\mathcal {A}_2(\mathcal {A}_c\kappa _1,\kappa _2) +\mathcal {A}_2(\kappa _1,\mathcal {A}_c\kappa _2),}} \end{aligned}$$

then (5.24) can be rewritten as

$$\begin{aligned} {{\mathcal {A}_2(\mathcal {A}_c\kappa _1,\kappa _2)+\mathcal {A}_2(\kappa _1,\mathcal {A}_c\kappa _2)=\mathcal {A}_h\mathcal {A}_2(\kappa _1,\kappa _2) +\frac{1}{2!}\Pi _hD^2\mathcal {P}(0_\mathcal {X})(\kappa _1,\kappa _2).}} \end{aligned}$$

Due to the fact that $\mathcal {A}_2$ is a linear operator, we need only to compute $\mathcal {A}_2(\overline{\nu }_1,\overline{\nu }_1),\mathcal {A}_2(\overline{\nu }_1,\overline{\nu }_2),\mathcal {A}_2(\overline{\nu }_2,\overline{\nu }_2)$, $\mathcal {A}_2(\overline{\nu }_2,\overline{\nu }_3),\mathcal {A}_2(\overline{\nu }_1,\overline{\nu }_3),\mathcal {A}_2(\overline{\nu }_3,\overline{\nu }_3)$.

(i) Computation of ${{\mathcal {A}_2(\overline{\nu }_1,\overline{\nu }_1)}}$. Due to $\frac{1}{2!}\Pi _hD^2{{\mathcal {P}(0_\mathcal {X})}}(\overline{\nu }_1,\overline{\nu }_1)=0$ and $\mathcal {A}_c\overline{\nu }_1=0$, the equation
  $$\begin{aligned} {{\mathcal {A}_2(\mathcal {A}_c\overline{\nu }_1,\overline{\nu }_1)+\mathcal {A}_2(\overline{\nu }_1,\mathcal {A}_c\overline{\nu }_1) =\mathcal {A}_h\mathcal {A}_2(\overline{\nu }_1,\overline{\nu }_1) +\frac{1}{2!}\Pi _hD^2\mathcal {P}(0_\mathcal {X})(\overline{\nu }_1,\overline{\nu }_1)}} \end{aligned}$$
  is equivalent to ${{0=\mathcal {A}_h\mathcal {A}_2(\overline{\nu }_1,\overline{\nu }_1). }}$ Since 0 belongs to the resolvent set of $\mathcal {A}_h$, we obtain that
  5.25 $$\begin{aligned} {{\mathcal {A}_2(\overline{\nu }_1,\overline{\nu }_1)=0.}} \end{aligned}$$
(ii) Computation of ${{\mathcal {A}_2(\overline{\nu }_1,\overline{\nu }_2)}}$. Because of $\mathcal {A}_c\overline{\nu }_1=0$ and $\mathcal {A}_c\overline{\nu }_2=\omega _*i\overline{\nu }_2$, the equation
  $$\begin{aligned} {{\mathcal {A}_2(\mathcal {A}_c\overline{\nu }_1,\overline{\nu }_2)+\mathcal {A}_2(\overline{\nu }_1,\mathcal {A}_c\overline{\nu }_2) =\mathcal {A}_h\mathcal {A}_2(\overline{\nu }_1,\overline{\nu }_2) +\frac{1}{2!}\Pi _hD^2\mathcal {P}(0_\mathcal {X})(\overline{\nu }_1,\overline{\nu }_2)}} \end{aligned}$$
  is equivalent to $(\omega _*i-\mathcal {A}_h){{\mathcal {A}_2(\overline{\nu }_1,\overline{\nu }_2)}}=\frac{1}{2!}\Pi _hD^2{{\mathcal {P}(0_\mathcal {X})}}(\overline{\nu }_1,\overline{\nu }_2)$ with
  $$\begin{aligned}&D^2{{\mathcal {P}(0_\mathcal {X})}}(\overline{\nu }_1,\overline{\nu }_2)\\&\quad =\left( \begin{array}{{c}} 0_{\mathbb {R}}\\ D^2{{\mathcal {H}(0_\mathcal {X})}}\left( \begin{array}{{c}}\left( \begin{array}{{c}} 0_{\mathbb {R}}\\ \left( \begin{array}{{c}} 0_{\mathbb {R}^2}\\ \left( \begin{array}{{c}} 0_{L^1((0,+\infty ),\mathbb {R}^2)}\\ 0_{L^1((0,+\infty ),\mathbb {R}^2)}\\ \end{array} \right) \\ \end{array} \right) \\ \end{array} \right) ,\left( \begin{array}{{c}} 0_{\mathbb {R}}\\ \left( \begin{array}{{c}} 0_{\mathbb {R}^2}\\ \left( \begin{array}{{c}} \beta _1\\ \beta _2\\ \end{array} \right) \\ \end{array} \right) \\ \end{array} \right) \\ \end{array} \right) \\ \end{array} \right) \\&\quad =\left( \begin{array}{{c}} 0_{\mathbb {R}}\\ DF({\overline{p}_{+}})\left( \begin{array}{{c}} 0_{\mathbb {R}^2}\\ \left( \begin{array}{{c}} \beta _1\\ \beta _2\\ \end{array} \right) \\ \end{array} \right) \\ \end{array} \right) \\&\qquad +\left( \begin{array}{{c}} 0_{\mathbb {R}}\\ \tau _kD^2F({\overline{p}_{+}})\left( \begin{array}{{c}}\left( \begin{array}{{c}} 0_{\mathbb {R}^2}\\ \left( \begin{array}{{c}} \beta _1\\ \beta _2\\ \end{array} \right) \\ \end{array} \right) ,\left( \frac{\text{ d }{\overline{p}_{+}}_{\tilde{\tau }(t)+\tau _k}}{\text{ d } \tilde{\tau }}\right) \Bigg |_{\tilde{\tau }=0} \end{array} \right) \\ \end{array} \right) \\&\qquad -D{{\mathcal {K}(0_X)}}\left( \begin{array}{{c}} 0_{\mathbb {R}^2}\\ \left( \begin{array}{{c}} \beta _1\\ \beta _2\\ \end{array} \right) \\ \end{array} \right) . \end{aligned}$$
  Therefore, we have that $$D^2{{\mathcal {P}(0_\mathcal {X})}}(\overline{\nu }_1,\overline{\nu }_2)=\ell _1(\tau _k)\left( \begin{array}{{c}} 0_{\mathbb {R}}\\ \Theta _1\\ \end{array} \right) -\ell _2(\tau _k)\left( \begin{array}{{c}} 0_{\mathbb {R}}\\ \Theta _2\\ \end{array} \right) , $$ where the forms of $\ell _1(\tau _k)$ and $\ell _2(\tau _k)$ are given in Appendix A.2. Then from (5.14) and (5.15), we obtain
  5.26 $$\begin{aligned}&{{\mathcal {A}_2(\overline{\nu }_1,\overline{\nu }_2)}}\nonumber \\&\quad =\frac{1}{2}\ell _1(\tau _k)(\omega _*i-\mathcal {A}_h)^{-1}\Pi _h\left( \begin{array}{{c}} 0_{\mathbb {R}}\\ \Theta _1\\ \end{array} \right) -\frac{1}{2}\ell _2(\tau _k)(\omega _*i-\mathcal {A}_h)^{-1}\Pi _h\left( \begin{array}{{c}} 0_{\mathbb {R}}\\ \Theta _2\\ \end{array} \right) \nonumber \\&\quad =\frac{1}{2}\ell _1(\tau _k)\left( \begin{array}{{c}} 0_{\mathbb {R}}\\ \left( \omega _*i-\mathcal {A}_{\tau _k}^{\mathbb {C}}|_{\Pi _h^{\mathcal {A}_{\tau _k}}(X)}\right) ^{-1}\Pi _h^{\mathcal {A}_{\tau _k}} \Theta _1\\ \end{array} \right) \nonumber \\&\qquad -\frac{1}{2}\ell _2(\tau _k)\left( \begin{array}{{c}} 0_{\mathbb {R}}\\ \left( \omega _*i-\mathcal {A}_{\tau _k}^{\mathbb {C}}|_{\Pi _h^{\mathcal {A}_{\tau _k}}(X)}\right) ^{-1}\Pi _h^{\mathcal {A}_{\tau _k}} \Theta _2\\ \end{array} \right) =\left( \begin{array}{{c}} 0_{\mathbb {R}}\\ \left( \begin{array}{{c}} 0_{\mathbb {R}^2}\\ \left( \begin{array}{{c}} \widetilde{\varphi }_{111}(\tau _k)\\ \widetilde{\varphi }_{112}(\tau _k)\\ \end{array} \right) \\ \end{array} \right) \\ \end{array} \right) \end{aligned}$$
  with
  $$\begin{aligned} \widetilde{\varphi }_{111}(\tau _k)&=\frac{1}{2}\ell _1(\tau _k)\varphi _{11}(\lambda =\omega _*i) -\frac{1}{2}\ell _2(\tau _k)\varphi _{21}(\lambda =\omega _*i),\\ \widetilde{\varphi }_{112}(\tau _k)&=\frac{1}{2}\ell _1(\tau _k)\varphi _{12}(\lambda =\omega _*i) -\frac{1}{2}\ell _2(\tau _k)\varphi _{22}(\lambda =\omega _*i). \end{aligned}$$
  By making use of the similar method, we get the following results:
  5.27 $$\begin{aligned} {{\mathcal {A}_2(\overline{\nu }_1,\overline{\nu }_3)}}=\left( \begin{array}{{c}} 0_{\mathbb {R}}\\ \left( \begin{array}{{c}} 0_{\mathbb {R}^2}\\ \left( \begin{array}{{c}} \overline{\widetilde{\varphi }_{111}(\tau _k)}\\ \overline{\widetilde{\varphi }_{112}(\tau _k)}\\ \end{array} \right) \\ \end{array} \right) \\ \end{array} \right) ,{{\mathcal {A}_2(\overline{\nu }_2,\overline{\nu }_1)=\mathcal {A}_2(\overline{\nu }_1,\overline{\nu }_2).}} \end{aligned}$$
(iii) Computation of ${{\mathcal {A}_2(\overline{\nu }_2,\overline{\nu }_2)}}$. Since $\mathcal {A}_c\overline{\nu }_2=\omega _*i\overline{\nu }_2$, the equation
  $$\begin{aligned} {{\mathcal {A}_2(\mathcal {A}_c\overline{\nu }_2,\overline{\nu }_2)+\mathcal {A}_2(\overline{\nu }_2,\mathcal {A}_c\overline{\nu }_2) =\mathcal {A}_h\mathcal {A}_2(\overline{\nu }_2,\overline{\nu }_2) +\frac{1}{2!}\Pi _hD^2\mathcal {P}(0_\mathcal {X})(\overline{\nu }_2,\overline{\nu }_2)}} \end{aligned}$$
  is equivalent to $(2\omega _*i-\mathcal {A}_h){{\mathcal {A}_2(\overline{\nu }_2,\overline{\nu }_2)}}=\frac{1}{2!}\Pi _hD^2{{\mathcal {P}(0_\mathcal {X})}}(\overline{\nu }_2,\overline{\nu }_2)$ with
  $$\begin{aligned} D^2{{\mathcal {P}(0_\mathcal {X})}}(\overline{\nu }_2,\overline{\nu }_2)&=\left( \begin{array}{{c}} 0_{\mathbb {R}}\\ D^2{{\mathcal {H}(0_\mathcal {X})}}\left( \begin{array}{{c}} 0_{\mathbb {R}}\\ \left( \begin{array}{{c}} 0_{\mathbb {R}^2}\\ \left( \begin{array}{{c}} \beta _1\\ \beta _2\\ \end{array} \right) \\ \end{array} \right) \\ \end{array} \right) ^2\\ \end{array} \right) \\&=\left( \begin{array}{{c}} 0_{\mathbb {R}}\\ \tau _kD^2F(\overline{p}_{+})\left( \begin{array}{{c}} 0_{\mathbb {R}^2}\\ \left( \begin{array}{{c}} \beta _1\\ \beta _2\\ \end{array} \right) \\ \end{array} \right) ^2\\ \end{array} \right) . \end{aligned}$$
  Then we get that $$D^2{{\mathcal {P}(0_\mathcal {X})(\overline{\nu }_2,\overline{\nu }_2)}}=\ell _3(\tau _k)\left( \begin{array}{{c}} 0_{\mathbb {R}}\\ \Theta _1\\ \end{array} \right) -\ell _4(\tau _k)\left( \begin{array}{{c}} 0_{\mathbb {R}}\\ \Theta _2\\ \end{array} \right) , $$ where the forms of $\ell _3(\tau _k)$ and $\ell _4(\tau _k)$ are given in Appendix A.2. Similarly, on the basis of (5.14), (5.15) and by using the similar method to (5.26), we can get
  5.28 $$\begin{aligned} {{\mathcal {A}_2(\overline{\nu }_2,\overline{\nu }_2)}} =\left( \begin{array}{{c}} 0_{\mathbb {R}}\\ \left( \begin{array}{{c}} 0_{\mathbb {R}^2}\\ \left( \begin{array}{{c}} \widetilde{\varphi }_{113}(\tau _k)\\ \widetilde{\varphi }_{114}(\tau _k)\\ \end{array} \right) \\ \end{array} \right) \\ \end{array} \right) \end{aligned}$$
  with
  $$\begin{aligned} \widetilde{\varphi }_{113}(\tau _k)&=\frac{1}{2}\ell _3(\tau _k)\varphi _{11}(\lambda =2\omega _*i) -\frac{1}{2}\ell _4(\tau _k)\varphi _{21}(\lambda =2\omega _*i),\\ \widetilde{\varphi }_{114}(\tau _k)&=\frac{1}{2}\ell _3(\tau _k)\varphi _{12}(\lambda =2\omega _*i) -\frac{1}{2}\ell _4(\tau _k)\varphi _{22}(\lambda =2\omega _*i). \end{aligned}$$
(iv) Computation of ${{\mathcal {A}_2(\overline{\nu }_2,\overline{\nu }_3)}}$. Since $\mathcal {A}_c\overline{\nu }_2=\omega _*i\overline{\nu }_2$ and $\mathcal {A}_c\overline{\nu }_3=-\omega _*i\overline{\nu }_3$, then the equation
  $$\begin{aligned} {{\mathcal {A}_2(\mathcal {A}_c\overline{\nu }_2,\overline{\nu }_3)+\mathcal {A}_2(\overline{\nu }_2,\mathcal {A}_c\overline{\nu }_3) =\mathcal {A}_h\mathcal {A}_2(\overline{\nu }_2,\overline{\nu }_3) +\frac{1}{2!}\Pi _hD^2\mathcal {P}(0_\mathcal {X})(\overline{\nu }_2,\overline{\nu }_3)}} \end{aligned}$$
  is converted to $(0-\mathcal {A}_h){{\mathcal {A}_2(\overline{\nu }_2,\overline{\nu }_3)}}=\frac{1}{2!}\Pi _hD^2{{\mathcal {P}(0_\mathcal {X})}}(\overline{\nu }_2,\overline{\nu }_3)$ with
  $$\begin{aligned}&D^2{{\mathcal {P}(0_\mathcal {X})(\overline{\nu }_1,\overline{\nu }_2)}}&\quad =\left( \begin{array}{{c}} 0_{\mathbb {R}}\\ \tau _kD^2F({\overline{p}_{+}})\left( \begin{array}{{c}}\left( \begin{array}{{c}} 0_{\mathbb {R}^2}\\ \left( \begin{array}{{c}} \beta _1\\ \beta _2\\ \end{array} \right) \\ \end{array} \right) ,\left( \begin{array}{{c}} 0_{\mathbb {R}^2}\\ \left( \begin{array}{{c}} \beta _3\\ \beta _4\\ \end{array} \right) \\ \end{array} \right) \\ \end{array} \right) \\ \end{array} \right) . \end{aligned}$$
  Thus we get $$D^2{{\mathcal {P}(0_\mathcal {X})}}(\overline{\nu }_2,\overline{\nu }_3)=\ell _5(\tau _k)\left( \begin{array}{{c}} 0_{\mathbb {R}}\\ \Theta _1\\ \end{array} \right) -\ell _6(\tau _k)\left( \begin{array}{{c}} 0_{\mathbb {R}}\\ \Theta _2\\ \end{array} \right) , $$ where the forms of $\ell _5(\tau _k)$ and $\ell _6(\tau _k)$ are given in Appendix A.2. Therefore, on the basis of (5.14) and (5.15), we get
  5.29 $$\begin{aligned} {{\mathcal {A}_2(\overline{\nu }_2,\overline{\nu }_3)}} =\left( \begin{array}{{c}} 0_{\mathbb {R}}\\ \left( \begin{array}{{c}} 0_{\mathbb {R}^2}\\ \left( \begin{array}{{c}} \widetilde{\varphi }_{115}(\tau _k)\\ \widetilde{\varphi }_{116}(\tau _k)\\ \end{array} \right) \\ \end{array} \right) \\ \end{array} \right) \end{aligned}$$
  with
  $$\begin{aligned} \widetilde{\varphi }_{115}(\tau _k)&=\frac{1}{2}\ell _5(\tau _k)\varphi _{11}(\lambda =0) -\frac{1}{2}\ell _6(\tau _k)\varphi _{21}(\lambda =0),\\ \widetilde{\varphi }_{116}(\tau _k)&=\frac{1}{2}\ell _5(\tau _k)\varphi _{12}(\lambda =0) -\frac{1}{2}\ell _6(\tau _k)\varphi _{22}(\lambda =0). \end{aligned}$$
  By the above method, we obtain the result as follows:
  5.30 $$\begin{aligned} {{\mathcal {A}}}_{{{2}}}(\overline{{{\nu }}}_{{{3}}},\overline{{{\nu }}}_{{{1}}})&= {{\mathcal {A}}}_{{{2}}}(\overline{{{\nu }}}_{{{1}}},\overline{{{\nu }}}_{{{3}}}),\; {{\mathcal {A}}}_{{{2}}}(\overline{{{\nu }}}_{{{3}}},\overline{{{\nu }}}_{{{2}}}) ={{\mathcal {A}}}_{{{2}}}(\overline{{{\nu }}}_{{{2}}},\overline{{{\nu }}}_{{{3}}}),\; {{\mathcal {A}}}_{{{2}}}(\overline{{{\nu }}}_{{{3}}},\overline{{{\nu }}}_{{{3}}})\nonumber \\&=\left( \begin{array}{{c}} 0_{\mathbb {R}}\\ \left( \begin{array}{{c}} 0_{\mathbb {R}^2}\\ \left( \begin{array}{{c}} \overline{\widetilde{\varphi }_{113}(\tau _k)}\\ \overline{\widetilde{\varphi }_{114}(\tau _k)}\\ \end{array} \right) \\ \end{array} \right) \\ \end{array} \right) . \end{aligned}$$
  Let $\widehat{\nu }_1=\overline{\nu }_1,~\widehat{\nu }_2=\overline{\nu }_2+\overline{\nu }_3,~ \widehat{\nu }_3=\frac{1}{i}(\overline{\nu }_2-\overline{\nu }_3)$, then we have
  $$\begin{aligned} {{\mathcal {A}}}_{{{2}}}(\widehat{{{\nu }}}_{{{1}}},\widehat{{{\nu }}}_{{{1}}})&={{0}},\;{{\mathcal {A}}}_{{{2}}} (\widehat{{{\nu }}}_{{{1}}},\widehat{{{\nu }}}_{{{2}}}) ={{\mathcal {A}}}_{{{2}}}(\widehat{{{\nu }}}_{{{2}}},\widehat{{{\nu }}}_{{{1}}}) =\left( \begin{array}{{c}} 0_{\mathbb {R}}\\ \left( \begin{array}{{c}} 0_{\mathbb {R}^2}\\ \left( \begin{array}{{c}} 2\text{ Re }(\widetilde{\varphi }_{111}(\tau _k))\\ 2\text{ Re }(\widetilde{\varphi }_{112}(\tau _k))\\ \end{array} \right) \\ \end{array} \right) \\ \end{array} \right) ,\\ {{\mathcal {A}}}_{{{2}}}(\widehat{{{\nu }}}_{{{1}}},\widehat{{{\nu }}}_{{{3}}})&= {{\mathcal {A}}}_{{{2}}}(\widehat{{{\nu }}}_{{{3}}},\widehat{{{\nu }}}_{{{1}}}) =\left( \begin{array}{{c}} 0_{\mathbb {R}}\\ \left( \begin{array}{{c}} 0_{\mathbb {R}^2}\\ \left( \begin{array}{{c}} 2\text{ Im }(\widetilde{\varphi }_{111}(\tau _k))\\ 2\text{ Im }(\widetilde{\varphi }_{112}(\tau _k))\\ \end{array} \right) \\ \end{array} \right) \\ \end{array} \right) ,\\ {{\mathcal {A}}}_{{{2}}}(\widehat{{{\nu }}}_{{{2}}},\widehat{{{\nu }}}_{{{2}}})&=\left( \begin{array}{{c}} 0_{\mathbb {R}}\\ \left( \begin{array}{{c}} 0_{\mathbb {R}^2}\\ \left( \begin{array}{{c}} 2\text{ Re }(\widetilde{\varphi }_{113}(\tau _k))+2\widetilde{\varphi }_{115}(\tau _k)\\ 2\text{ Re }(\widetilde{\varphi }_{114}(\tau _k))+2\widetilde{\varphi }_{116}(\tau _k)\\ \end{array} \right) \\ \end{array} \right) \\ \end{array} \right) ,\\ {{\mathcal {A}}}_{{{2}}}(\widehat{{{\nu }}}_{{{2}}},\widehat{{{\nu }}}_{{{3}}})&= {{\mathcal {A}}}_{{{2}}}(\widehat{{{\nu }}}_{{{3}}},\widehat{{{\nu }}}_{{{2}}}) =\left( \begin{array}{{c}} 0_{\mathbb {R}}\\ \left( \begin{array}{{c}} 0_{\mathbb {R}^2}\\ \left( \begin{array}{{c}} 2\text{ Im }(\widetilde{\varphi }_{113}(\tau _k))\\ 2\text{ Im }(\widetilde{\varphi }_{114}(\tau _k))\\ \end{array} \right) \\ \end{array} \right) \\ \end{array} \right) ,\\ {{\mathcal {A}}}_{{{2}}}(\widehat{{{\nu }}}_{{{3}}},\widehat{{{\nu }}}_{{{3}}})&=\left( \begin{array}{{c}} 0_{\mathbb {R}}\\ \left( \begin{array}{{c}} 0_{\mathbb {R}^2}\\ \left( \begin{array}{{c}} -2\text{ Re }(\widetilde{\varphi }_{113}(\tau _k))+2\widetilde{\varphi }_{115}(\tau _k)\\ -2\text{ Re }(\widetilde{\varphi }_{114}(\tau _k))+2\widetilde{\varphi }_{116}(\tau _k)\\ \end{array} \right) \\ \end{array} \right) \\ \end{array} \right) . \end{aligned}$$

### Normal form of the reduced system

In this section, we calculate the normal form of the reduced system (5.7). Firstly, we let $\mathcal {T}\subset \mathcal {X}$, and $J^{\widetilde{m}}(\mathcal {X}_c,\mathcal {T})$ be the linear space of homogeneous polynomials of degree $\widetilde{m}(\widetilde{m}=2,3)$. Define $\mathcal {S}_{\widetilde{m}}^c:J^{\widetilde{m}}(\mathcal {X}_c,\mathcal {X}_c)\rightarrow J^{\widetilde{m}}(\mathcal {X}_c,\mathcal {X}_c)$ by

5.31 $$\begin{aligned} \mathcal {S}_{\widetilde{m}}^c(\mathcal {U}_c)=[\mathcal {A}_c,\mathcal {U}_c],\quad \forall \,\mathcal {U}_c\in J^{\widetilde{m}}(\mathcal {X}_c,\mathcal {X}_c), \end{aligned}$$

where $[\mathcal {A},\mathcal {U}](\varphi _c)$ is defined by $[\mathcal {A},\mathcal {U}](\varphi _c)=D\mathcal {U}(\varphi _c)(\mathcal {A}\varphi _c)-\mathcal {A}\mathcal {U}(\varphi _c),~\varphi _c\in \mathcal {X}_c.$ Let $\mathcal {S}_{\widetilde{m}}^h:J^{\widetilde{m}}(\mathcal {X}_c,\mathcal {X}_h\cap D(\mathcal {A}))\rightarrow J^{\widetilde{m}}(\mathcal {X}_c,\mathcal {X}_h)$ by

5.32 $$\begin{aligned} \mathcal {S}_{\widetilde{m}}^h(\mathcal {U}_h)=[\mathcal {A},\mathcal {U}_h],\quad \forall \,\mathcal {U}_h\in J^{\widetilde{m}}(\mathcal {X}_c,\mathcal {X}_h\cap D(\mathcal {A})). \end{aligned}$$

Next we decompose $J^{\widetilde{m}}(\mathcal {X}_c,\mathcal {X}_c)$ into the direct sum $J^{\widetilde{m}}(\mathcal {X}_c,\mathcal {X}_c)=\mathcal {R}_{\widetilde{m}}^c\oplus \mathcal {Y}_{\widetilde{m}}^c,$ where $\mathcal {R}_{\widetilde{m}}^c=\text{ Ran }(\mathcal {S}_{\widetilde{m}}^c)$ is the range of $\mathcal {S}_{\widetilde{m}}^c$, and $\mathcal {Y}_{\widetilde{m}}^c$ is some complementary space of $\mathcal {R}_{\widetilde{m}}^c$ into $J^{\widetilde{m}}(\mathcal {X}_c,\mathcal {X}_c)$. Let $\mathcal {D}_{\widetilde{m}}:J^{\widetilde{m}}(\mathcal {X}_c,\mathcal {X})\rightarrow J^{\widetilde{m}}(\mathcal {X}_c,\mathcal {X})$ be the bounded linear projector satisfying

$$\begin{aligned} \mathcal {D}_{\widetilde{m}}(J^{\widetilde{m}}(\mathcal {X}_c,\mathcal {X}))=\mathcal {R}_{\widetilde{m}}^c\oplus J^{\widetilde{m}}(\mathcal {X}_c,\mathcal {X}_h)\quad \text{ and }\quad (I-\mathcal {D}_{\widetilde{m}})(J^{\widetilde{m}}(\mathcal {X}_c,\mathcal {X}))=\mathcal {Y}_{\widetilde{m}}^c. \end{aligned}$$

Then we calculate $\mathcal {U}_2\in J^2(\mathcal {X}_c,D(\mathcal {A}))$ such that

5.33 $$\begin{aligned} {[}\mathcal {A},\mathcal {U}_2](\kappa _c)=\mathcal {D}_2\left[ \frac{1}{2!}D^2{{\mathcal {P}(0_\mathcal {X})}}(\kappa _c,\kappa _c)\right] ,\quad \forall \, \kappa _c\in \mathcal {X}_c. \end{aligned}$$

Thus the normal form of the reduced system is

5.34 $$\begin{aligned} \frac{\text{ d } \kappa _c(t)}{\text{ d } t}&=\mathcal {A}_c\kappa _c(t)+\frac{1}{2!}\Pi _cD^2{{\mathcal {P}_3(0_\mathcal {X})}}(\kappa _c(t),\kappa _c(t))\nonumber \\&\quad +\frac{1}{3!}\Pi _cD^3{{\mathcal {P}_3(0_\mathcal {X})}}(\kappa _c(t),\kappa _c(t),\kappa _c(t))+R_c(\kappa _c(t)) \end{aligned}$$

with

5.35 $$\begin{aligned} \frac{1}{2!}\Pi _cD^2{{\mathcal {P}_3(0_\mathcal {X})}}(\kappa _c,\kappa _c)&=\frac{1}{2!}\Pi _cD^2{{\mathcal {P}_2(0_\mathcal {X})}}(\kappa _c,\kappa _c)\nonumber \\&=\frac{1}{2!}\Pi _cD^2{{\mathcal {P}(0_\mathcal {X})}}(\kappa _c,\kappa _c)-[\mathcal {A}_c,\Pi _c\mathcal {U}_2](\kappa _c) \end{aligned}$$

and

5.36 $$\begin{aligned} \frac{1}{3!}\Pi _cD^3{{\mathcal {P}_3(0_\mathcal {X})}}(\kappa _c,\kappa _c,\kappa _c)&=\frac{1}{3!}\Pi _cD^3{{\mathcal {P}_2(0_\mathcal {X})}}(\kappa _c,\kappa _c,\kappa _c)-\Pi _c[\mathcal {A},\mathcal {U}_3](\kappa _c)\nonumber \\&=\frac{1}{3!}\Pi _cD^3{{\mathcal {P}_2(0_\mathcal {X})}}(\kappa _c,\kappa _c,\kappa _c)-[\mathcal {A}_c,\Pi _c\mathcal {U}_3](\kappa _c), \end{aligned}$$

where

5.37 $$\begin{aligned} \frac{1}{3!}\Pi _cD^3{{\mathcal {P}_2(0_\mathcal {X})}}(\kappa _c,\kappa _c,\kappa _c)&=\Pi _cD^2{{\mathcal {P}(0_\mathcal {X})}}(\kappa _c,\mathcal {U}_2(\kappa _c))\nonumber \\&\quad +\frac{1}{3!}\Pi _cD^3{{\mathcal {P}(0_\mathcal {X})}}(\kappa _c,\kappa _c,\kappa _c)\nonumber \\&\quad -D\Pi _c\mathcal {U}_2(\kappa _c)\nonumber \\&\quad \times \left[ \frac{1}{2!}\Pi _cD^2{{\mathcal {P}(0_\mathcal {X})}}(\kappa _c,\kappa _c) -[\mathcal {A}_c,\Pi _c\mathcal {U}_2]\right] (\kappa _c). \end{aligned}$$

Next, we compute the normal form expressed in terms of the basis ${{\{\overline{\nu }_1,\overline{\nu }_2,\overline{\nu }_3\}}}$. We consider $J^{\widetilde{m}}(\mathbb {C}^3,\mathcal {X}_h\cap D(\mathcal {A}))$ and $J^{\widetilde{m}}(\mathbb {C}^3,\mathbb {C}^3)$, which define the linear space of the homogeneous polynomials of degree $\widetilde{m}$ in three real variables: ${{\tilde{\tau },\varsigma _1,~\varsigma _2}}$ with coefficients in $\mathbb {C}^3$ and $\mathcal {X}_h\cap D(\mathcal {A})$, respectively. The operators $\mathcal {S}_{\widetilde{m}}^c$ and $\mathcal {S}_{\widetilde{m}}^h$ act in the spaces $J^{\widetilde{m}}(\mathbb {C}^3,\mathbb {C}^3)$ and $J^{\widetilde{m}}(\mathbb {C}^3,\mathcal {X}_h\cap D(\mathcal {A}))$, respectively, and satisfying

5.38 $$\begin{aligned} \mathcal {S}_{\widetilde{m}}^c(\mathcal {U}_{{\widetilde{m}},c})\left( \begin{array}{{c}} \tilde{\tau }\\ \varsigma _1\\ \varsigma _2\\ \end{array} \right)&=[\mathcal {A}_c,\mathcal {U}_{{\widetilde{m}},c}]\left( \begin{array}{{c}} \tilde{\tau }\\ \varsigma _1\\ \varsigma _2\\ \end{array} \right) =D\mathcal {U}_{{\widetilde{m}},c}\mathcal {A}_c\left( \begin{array}{{c}} \tilde{\tau }\\ \varsigma _1\\ \varsigma _2\\ \end{array} \right) -\mathcal {A}_c\mathcal {U}_{{\widetilde{m}},c}\left( \begin{array}{{c}} \tilde{\tau }\\ \varsigma _1\\ \varsigma _2\\ \end{array} \right) \nonumber \\&=\left( \begin{array}{{c}} D\left( \begin{array}{{c}} \mathcal {U}_{{\widetilde{m}},c}^1\\ \mathcal {U}_{{\widetilde{m}},c}^2\\ \end{array} \right) \left( \begin{array}{{c}} \tilde{\tau }\\ \varsigma _1\\ \varsigma _2\\ \end{array} \right) \mathcal {M}_c\left( \begin{array}{{c}} \varsigma _1\\ \varsigma _2\\ \end{array} \right) -\mathcal {M}_c\left( \begin{array}{{c}} \mathcal {U}_{{\widetilde{m}},c}^1\\ \mathcal {U}_{{\widetilde{m}},c}^2\\ \end{array} \right) \left( \begin{array}{{c}} \tilde{\tau }\\ \varsigma _1\\ \varsigma _2\\ \end{array} \right) \\ D\mathcal {U}_{{\widetilde{m}},c}^3\left( \begin{array}{{c}} \tilde{\tau }\\ \varsigma _1\\ \varsigma _2\\ \end{array} \right) \mathcal {M}_c(\varsigma _1,\varsigma _2)\\ \end{array} \right) \end{aligned}$$

and

5.39 $$\begin{aligned} \mathcal {S}_{\widetilde{m}}^h(\mathcal {U}_{{\widetilde{m}},h})=[\mathcal {A},\mathcal {U}_{{\widetilde{m}},h}]=D\mathcal {U}_{{\widetilde{m}},h}\mathcal {A}_c-\mathcal {A}_h\mathcal {U}_{{\widetilde{m}},h}, \end{aligned}$$

where

$$\begin{aligned} \mathcal {U}_{{\widetilde{m}},c}\left( \begin{array}{{c}} \tilde{\tau }\\ \varsigma _1\\ \varsigma _2\\ \end{array} \right) =\left( \begin{array}{{c}} \mathcal {U}_{{\widetilde{m}},c}^1\\ \mathcal {U}_{{\widetilde{m}},c}^2\\ \mathcal {U}_{{\widetilde{m}},c}^3\\ \end{array} \right) \left( \begin{array}{{c}} \tilde{\tau }\\ \varsigma _1\\ \varsigma _2\\ \end{array} \right) \in J^{\widetilde{m}}(\mathbb {C}^3,\mathbb {C}^3),~\mathcal {U}_{{\widetilde{m}},h}\in J^{\widetilde{m}}(\mathbb {C}^3,\mathcal {X}_h\cap D(\mathcal {A})) \end{aligned}$$

and

$$\begin{aligned} \mathcal {A}_c=\left( \begin{array}{{ccc}} 0&{}0&{}0\\ 0&{}\omega _*i&{}0\\ 0&{}0&{}-\omega _*i\\ \end{array} \right) ,\;\mathcal {M}_c=\left( \begin{array}{{cc}} \omega _*i&{}0\\ 0&{}-\omega _*i\\ \end{array} \right) . \end{aligned}$$

Define $$\widetilde{\mathcal {S}}_{{\widetilde{m}}}^c:J^{\widetilde{m}}(\mathbb {C}^3,\mathbb {C}^2)\rightarrow J^{\widetilde{m}}(\mathbb {C}^3,\mathbb {C}^2)$$ by

5.40 $$\begin{aligned} \widetilde{\mathcal {S}}_{{\widetilde{m}}}^c\left( \begin{array}{{c}} \mathcal {U}_{{\widetilde{m}},c}^1\\ \mathcal {U}_{{\widetilde{m}},c}^2\\ \end{array} \right) =D\left( \begin{array}{{c}} \mathcal {U}_{{\widetilde{m}},c}^1\\ \mathcal {U}_{{\widetilde{m}},c}^2\\ \end{array} \right) \mathcal {M}_c\left( \begin{array}{{c}} \varsigma _1\\ \varsigma _2\\ \end{array} \right) -\mathcal {M}_c\left( \begin{array}{{c}} \mathcal {U}_{{\widetilde{m}},c}^1\\ \mathcal {U}_{{\widetilde{m}},c}^2\\ \end{array} \right) ,\quad \forall \,\left( \begin{array}{{c}} \mathcal {U}_{{\widetilde{m}},c}^1\\ \mathcal {U}_{{\widetilde{m}},c}^2\\ \end{array} \right) \in J^{\widetilde{m}}(\mathbb {C}^3,\mathbb {C}^2), \end{aligned}$$

then the canonical basis of $J^2(\mathbb {R}^3,\mathbb {R}^2)$ is

$$\begin{aligned}{} & {} \left( \begin{array}{{c}} \varsigma _1^2\\ 0\\ \end{array} \right) ,\left( \begin{array}{{c}} \varsigma _1\varsigma _2\\ 0\\ \end{array} \right) ,\left( \begin{array}{{c}} \varsigma _1\tilde{\tau }\\ 0\\ \end{array} \right) ,\left( \begin{array}{{c}} \varsigma _2^2\\ 0\\ \end{array} \right) ,\left( \begin{array}{{c}} \varsigma _2\tilde{\tau }\\ 0\\ \end{array} \right) ,\left( \begin{array}{{c}} \tilde{\tau }^2\\ 0\\ \end{array} \right) ,\\ \\{} & {} \left( \begin{array}{{c}} 0\\ \varsigma _1^2\\ \end{array} \right) ,\left( \begin{array}{{c}} 0\\ \varsigma _1\varsigma _2\\ \end{array} \right) ,\left( \begin{array}{{c}} 0\\ \varsigma _1\tilde{\tau }\\ \end{array} \right) ,\left( \begin{array}{{c}} 0\\ \varsigma _2^2\\ \end{array} \right) ,\left( \begin{array}{{c}} 0\\ \varsigma _2\tilde{\tau }\\ \end{array} \right) ,\left( \begin{array}{{c}} 0\\ \tilde{\tau }^2\\ \end{array} \right) . \end{aligned}$$

Their corresponding images under $\frac{1}{\omega _*i}\widetilde{\mathcal {S}}_{2}^c$ are

$$\begin{aligned}{} & {} \left( \begin{array}{{c}} -2\varsigma _1\varsigma _2\\ -\varsigma _1^2\\ \end{array} \right) ,\left( \begin{array}{{c}} \varsigma _1^2-\varsigma _2^2\\ -\varsigma _1\varsigma _2\\ \end{array} \right) ,\left( \begin{array}{{c}} -\varsigma _2\tilde{\tau }\\ -\varsigma _1\tilde{\tau }\\ \end{array} \right) ,\left( \begin{array}{{c}} 2\varsigma _1\varsigma _2\\ -\varsigma _2^2\\ \end{array} \right) ,\left( \begin{array}{{c}} \varsigma _1\tilde{\tau }\\ -\varsigma _2\tilde{\tau }\\ \end{array} \right) ,\left( \begin{array}{{c}} 0\\ -\tilde{\tau }^2\\ \end{array} \right) ,\\ \\{} & {} \left( \begin{array}{{c}} \varsigma _1^2\\ -2\varsigma _1\varsigma _2\\ \end{array} \right) ,\left( \begin{array}{{c}} \varsigma _1\varsigma _2\\ \varsigma _1^2-\varsigma _2^2\\ \end{array} \right) ,\left( \begin{array}{{c}} \varsigma _1\tilde{\tau }\\ -\varsigma _2\tilde{\tau }\\ \end{array} \right) ,\left( \begin{array}{{c}} \varsigma _2^2\\ 2\varsigma _1\varsigma _2\\ \end{array} \right) ,\left( \begin{array}{{c}} \varsigma _2\tilde{\tau }\\ \varsigma _1\tilde{\tau }\\ \end{array} \right) ,\left( \begin{array}{{c}} \tilde{\tau }^2\\ 0\\ \end{array} \right) . \end{aligned}$$

In addition, a complementary space of $\text{ Ran }(\widetilde{\mathcal {S}}_{2}^c)$ in $J^2(\mathbb {R}^3,\mathbb {R}^2)$ is

$$\begin{aligned} \text{ Ran }(\widetilde{\mathcal {S}}_{2}^c)^c=\text{ span }\left\{ \left( \begin{array}{{c}} \varsigma _1\tilde{\tau }\\ 0\\ \end{array} \right) ,\left( \begin{array}{{c}} 0\\ \varsigma _2\tilde{\tau }\\ \end{array} \right) \right\} . \end{aligned}$$

Consequently, the canonical basis of $J^3(\mathbb {R}^3,\mathbb {R}^2)$ is

$$\begin{aligned}{} & {} \left( \begin{array}{{c}} \varsigma _1^3\\ 0\\ \end{array} \right) ,\left( \begin{array}{{c}} \varsigma _1^2\varsigma _2\\ 0\\ \end{array} \right) ,\left( \begin{array}{{c}} \varsigma _1^2\tilde{\tau }\\ 0\\ \end{array} \right) ,\left( \begin{array}{{c}} \varsigma _1\varsigma _2^2\\ 0\\ \end{array} \right) ,\left( \begin{array}{{c}} \varsigma _1\varsigma _2\tilde{\tau }\\ 0\\ \end{array} \right) ,\\ \\{} & {} \left( \begin{array}{{c}} \varsigma _1\tilde{\tau }^2\\ 0\\ \end{array} \right) , \left( \begin{array}{{c}} \varsigma _2^3\\ 0\\ \end{array} \right) ,\left( \begin{array}{{c}} \varsigma _2^2\tilde{\tau }\\ 0\\ \end{array} \right) ,\left( \begin{array}{{c}} \varsigma _2\tilde{\tau }^2\\ 0\\ \end{array} \right) ,\left( \begin{array}{{c}} \tilde{\tau }^3\\ 0\\ \end{array} \right) ,\\{} & {} \left( \begin{array}{{c}} 0\\ \varsigma _1^3\\ \end{array} \right) ,\left( \begin{array}{{c}} 0\\ \varsigma _1^2\varsigma _2\\ \end{array} \right) ,\left( \begin{array}{{c}} 0\\ \varsigma _1^2\tilde{\tau }\\ \end{array} \right) ,\left( \begin{array}{{c}} 0\\ \varsigma _1\varsigma _2^2\\ \end{array} \right) ,\left( \begin{array}{{c}} 0\\ \varsigma _1\varsigma _2\tilde{\tau }\\ \end{array} \right) ,\\{} & {} \left( \begin{array}{{c}} 0\\ \varsigma _1\tilde{\tau }^2\\ \end{array} \right) , \left( \begin{array}{{c}} 0\\ \varsigma _2^3\\ \end{array} \right) ,\left( \begin{array}{{c}} 0\\ \varsigma _2^2\tilde{\tau }\\ \end{array} \right) ,\left( \begin{array}{{c}} 0\\ \varsigma _2\tilde{\tau }^2\\ \end{array} \right) ,\left( \begin{array}{{c}} 0\\ \tilde{\tau }^3\\ \end{array} \right) . \end{aligned}$$

Their corresponding images under $\frac{1}{\omega _*i}\widetilde{\mathcal {S}}_{3}^c$ are

$$\begin{aligned}{} & {} \left( \begin{array}{{c}} -3\varsigma _1^2\varsigma _2\\ -\varsigma _1^3\\ \end{array} \right) ,\left( \begin{array}{{c}} \varsigma _1^3-2\varsigma _1\varsigma _2^2\\ -\varsigma _1^2\varsigma _2\\ \end{array} \right) ,\left( \begin{array}{{c}} -2\varsigma _1\varsigma _2\tilde{\tau }\\ -\varsigma _1^2\tilde{\tau }\\ \end{array} \right) ,\left( \begin{array}{{c}} 2\varsigma _1^2\varsigma _2-\varsigma _2^3\\ -\varsigma _1\varsigma _2^2\\ \end{array} \right) ,\left( \begin{array}{{c}} \varsigma _1^2\tilde{\tau }-\varsigma _2^2\tilde{\tau }\\ -\varsigma _1\varsigma _2\tilde{\tau }\\ \end{array} \right) ,\\{} & {} \left( \begin{array}{{c}} -\varsigma _2\tilde{\tau }^2\\ -\varsigma _1\tilde{\tau }^2\\ \end{array} \right) ,\left( \begin{array}{{c}} 3\varsigma _1\varsigma _2^2\\ -\varsigma _2^3\\ \end{array} \right) ,\left( \begin{array}{{c}} 2\varsigma _1\varsigma _2\tilde{\tau }\\ -\varsigma _2^2\tilde{\tau }\\ \end{array} \right) ,\left( \begin{array}{{c}} \varsigma _1\tilde{\tau }^2\\ -\varsigma _2\tilde{\tau }^2\\ \end{array} \right) ,\left( \begin{array}{{c}} 0\\ -\tilde{\tau }^3\\ \end{array} \right) ,\\{} & {} \left( \begin{array}{{c}} \varsigma _1^3\\ -3\varsigma _1^2\varsigma _2\\ \end{array} \right) ,\left( \begin{array}{{c}} \varsigma _1^2\varsigma _2\\ \varsigma _1^3-2\varsigma _1\varsigma _2^2\\ \end{array} \right) ,\left( \begin{array}{{c}} \varsigma _1^2\tilde{\tau }\\ -2\varsigma _1\varsigma _2\tilde{\tau }\\ \end{array} \right) ,\left( \begin{array}{{c}} \varsigma _1\varsigma _2^2\\ 2\varsigma _1^2\varsigma _2-\varsigma _2^3\\ \end{array} \right) ,\left( \begin{array}{{c}} \varsigma _1\varsigma _2\tilde{\tau }\\ \varsigma _1^2\tilde{\tau }-\varsigma _2^2\tilde{\tau }\\ \end{array} \right) ,\\{} & {} \left( \begin{array}{{c}} \varsigma _1\tilde{\tau }^2\\ -\varsigma _2\tilde{\tau }^2\\ \end{array} \right) ,\left( \begin{array}{{c}} \varsigma _2^3\\ 3\varsigma _1\varsigma _2^2\\ \end{array} \right) ,\left( \begin{array}{{c}} \varsigma _2^2\tilde{\tau }\\ 2\varsigma _1\varsigma _2\tilde{\tau }\\ \end{array} \right) ,\left( \begin{array}{{c}} \varsigma _2\tilde{\tau }^2\\ \varsigma _1\tilde{\tau }^2\\ \end{array} \right) ,\left( \begin{array}{{c}} \tilde{\tau }^3\\ 0\\ \end{array} \right) . \end{aligned}$$

A complementary space of $\text{ Ran }(\widetilde{\mathcal {S}}_{3}^c)$ in $J^3(\mathbb {R}^3,\mathbb {R}^2)$ is

$$\begin{aligned} \text{ Ran }(\widetilde{\mathcal {S}}_{3}^c)^c=\text{ span }\left\{ \left( \begin{array}{{c}} \varsigma _1\tilde{\tau }^2\\ 0\\ \end{array} \right) ,\left( \begin{array}{{c}} \varsigma _1^2\varsigma _2\\ 0\\ \end{array} \right) ,\left( \begin{array}{{c}} 0\\ \varsigma _2\tilde{\tau }^2\\ \end{array} \right) ,\left( \begin{array}{{c}} 0\\ \varsigma _1\varsigma _2^2\\ \end{array} \right) \right\} . \end{aligned}$$

Now, define the following bounded linear projectors:

$$\begin{aligned} \text{ Proj}_{\widetilde{m}}^{\text{ R }}:J^{\widetilde{m}}(\mathbb {C}^3,\mathbb {C}^2)\rightarrow J^{\widetilde{m}}(\mathbb {C}^3,\mathbb {C}^2)\quad \text{ and }\quad \text{ Proj}_{\widetilde{m}}^{\text{ K }}:J^{\widetilde{m}}(\mathbb {C}^3,\mathbb {C}^2)\rightarrow J^{\widetilde{m}}(\mathbb {C}^3,\mathbb {C}^2) \end{aligned}$$

satisfying $$\text{ Proj}_{\widetilde{m}}^{\text{ R }}(J^{\widetilde{m}}(\mathbb {C}^3,\mathbb {C}^2))=\text{ Ran }(\widetilde{\mathcal {S}}_{{\widetilde{m}}}^c)\;\text{ and }\; \text{ Proj}_{\widetilde{m}}^{\text{ K }}(J^{\widetilde{m}}(\mathbb {C}^3,\mathbb {C}^2))=\text{ Ker }(\widetilde{\mathcal {S}}_{{\widetilde{m}}}^c).$$

Then we calculate the normal form of the reduced system expressed in terms of the basis ${{\{\overline{\nu }_1,\overline{\nu }_2,\overline{\nu }_3\}}}$ of $\mathcal {X}_c$. According to (5.21), (5.22), (5.37), (5.38), (5.39), we know that $\mathcal {U}_{2}\in J^2(\mathcal {X}_c,D(\mathcal {A}))$ denoted in (5.33) is equivalent to finding

$$\begin{aligned} \mathcal {U}_{2,c}&=\left( \begin{array}{{c}} \mathcal {U}_{2,c}^1\\ \mathcal {U}_{2,c}^2\\ \mathcal {U}_{2,c}^3\\ \end{array} \right) =\Pi _c\mathcal {U}_{2}(\mathbb {C}^3,\mathbb {C}^3)\in J^2(\mathbb {C}^3,\mathbb {C}^3)\quad \text{ and }\\ \mathcal {U}_{2,h}&=\Pi _h\mathcal {U}_{2}\in J^2(\mathbb {C}^3,\mathcal {X}_h\cap D(\mathcal {A})) \end{aligned}$$

such that

5.41 $$\begin{aligned} {[\mathcal {A}_c,\mathcal {U}_{2,c}]}\left( \begin{array}{{c}} \tilde{\tau }\\ \left( \begin{array}{{c}} \varsigma _1\\ \varsigma _2\\ \end{array} \right) \\ \end{array} \right)&=\left( \begin{array}{{c}} D\mathcal {U}_{2,c}^1\mathcal {M}_c(\varsigma _1,\varsigma _2)\\ D\left( \begin{array}{{c}} \mathcal {U}_{2,c}^2\\ \mathcal {U}_{2,c}^3\\ \end{array} \right) \mathcal {M}_c\left( \begin{array}{{c}} \varsigma _1\\ \varsigma _2\\ \end{array} \right) -\mathcal {M}_c\left( \begin{array}{{c}} \mathcal {U}_{2,c}^2\\ \mathcal {U}_{2,c}^3\\ \end{array} \right) \\ \end{array} \right) \nonumber \\&=\left( \begin{array}{{c}} 0_{\mathbb {R}}\\ \left( \begin{array}{{c}} 0_{\mathbb {R}^2}\\ \text{ Proj}_2^{\text{ R }}(\widetilde{\digamma })\\ \end{array} \right) \\ \end{array} \right) \end{aligned}$$

and

$$\begin{aligned} {[}\mathcal {A},\mathcal {U}_{2,h}]=D\mathcal {U}_{2,h}\mathcal {A}_c-\mathcal {A}_h\mathcal {U}_{2,h}=\left( \begin{array}{{c}} 0_{\mathbb {R}}\\ \left( \begin{array}{{c}} \widetilde{\widetilde{\varphi }}(\tau _k,\tilde{\tau })\\ \left( \begin{array}{{c}} \widehat{\overline{\overline{\varphi }}}_1(\tau _k,\tilde{\tau })\\ \widehat{\overline{\overline{\varphi }}}_2(\tau _k,\tilde{\tau })\\ \end{array} \right) \\ \end{array} \right) \\ \end{array} \right) , \end{aligned}$$

where

$$\begin{aligned} \widetilde{\digamma }\left( \begin{array}{{c}} \tilde{\tau }\\ \left( \begin{array}{{c}} \varsigma _1\\ \varsigma _2\\ \end{array} \right) \\ \end{array} \right) =\left( \begin{array}{{c}} \widetilde{\varphi }_1(\tau _k,\tilde{\tau })\\ \widetilde{\varphi }_2(\tau _k,\tilde{\tau })\\ \end{array} \right) =\left( \begin{array}{{c}} \digamma _1\varsigma _1\tilde{\tau }+\digamma _2\varsigma _2\tilde{\tau }+\ell _{20}\varsigma _1^2+\ell _{11}\varsigma _1\varsigma _2+\ell _{02}\varsigma _2^2\\ \overline{\digamma }_1\varsigma _2\tilde{\tau }+\overline{\digamma }_2\varsigma _1\tilde{\tau }+\overline{\ell }_{02}\varsigma _1^2 +\overline{\ell }_{11}\varsigma _1\varsigma _2+\overline{\ell }_{20}\varsigma _2^2\\ \end{array} \right) , \end{aligned}$$

and the forms of $\digamma _1,~\digamma _2,~\ell _{20},~\ell _{11}$ and $\ell _{02}$ are given in Appendix A.3.

According to (5.35), it is clear to get the second-order terms of the normal form expressed in terms of the basis $\{\overline{\nu }_1,\overline{\nu }_2,\overline{\nu }_3\}$ as follows:

5.42 $$\begin{aligned}&\frac{1}{2!}\Pi _cD^2{{\mathcal {P}_3(0_\mathcal {X})}}(\kappa _c,\kappa _c)\nonumber \\&\quad = (\overline{\nu }_1,\overline{\nu }_2,\overline{\nu }_3)\left( \begin{array}{{c}} 0_{\mathbb {R}^2}\\ \text{ Proj}_2^{\text{ K }}(\widetilde{\digamma })\\ \end{array} \right) =(\overline{\nu }_1,\overline{\nu }_2,\overline{\nu }_3)\left( \begin{array}{{c}} 0_{\mathbb {R}^2}\\ \left( \begin{array}{{c}} \left( \digamma _1+\frac{\overline{\digamma }_1}{2\omega _*}\right) \varsigma _1\tilde{\tau }\\ \left( \overline{\digamma }_1+\frac{\digamma _1}{2\omega _*}\right) \varsigma _2\tilde{\tau }\\ \end{array} \right) \\ \end{array} \right) \nonumber \\&\quad =\left( \digamma _1+\frac{\overline{\digamma }_1}{2\omega _*}\right) \tilde{\tau }\varsigma _1\overline{\nu }_2+ \left( \overline{\digamma }_1+\frac{\digamma _1}{2\omega _*}\right) \tilde{\tau }\varsigma _2\overline{\nu }_3. \end{aligned}$$

Note that the terms $O(|(\chi _1,\chi _2)|\tau ^2)$ are uncorrelated to determine the generic Hopf bifurcation. Thus, it needs only to calculate the coefficients of

$$\begin{aligned} \left( \begin{array}{{c}} 0\\ \left( \begin{array}{{c}} \varsigma _1^2\varsigma _2\\ 0\\ \end{array} \right) \\ \end{array} \right) ,\left( \begin{array}{{c}} 0\\ \left( \begin{array}{{c}} 0\\ \varsigma _1\varsigma _2^2\\ \end{array} \right) \\ \end{array} \right) \end{aligned}$$

in the third-order terms of the normal form. Firstly, from (5.28), (5.29) and (5.30), we get

$$\begin{aligned} \mathcal {U}_{2,h}\left( \begin{array}{{c}} 0\\ \left( \begin{array}{{c}} \varsigma _1\\ \varsigma _2\\ \end{array} \right) \\ \end{array} \right)&=\mathcal {U}_{2,h}(\varsigma _1\overline{\nu }_2+\varsigma _2\overline{\nu }_3) =\mathcal {A}_2(\varsigma _1\overline{\nu }_2+\varsigma _2\overline{\nu }_3,\varsigma _1\overline{\nu }_2+\varsigma _2\overline{\nu }_3)\\&=\varsigma _1^2\mathcal {A}_2(\overline{\nu }_2,\overline{\nu }_2)+2\varsigma _1\varsigma _2\mathcal {A}_2(\overline{\nu }_2,\overline{\nu }_3) +\varsigma _2^2\mathcal {A}_2(\overline{\nu }_3,\overline{\nu }_3)\\&=\left( \begin{array}{{c}} 0_{\mathbb {R}}\\ \left( \begin{array}{{c}} 0_{\mathbb {R}^2}\\ \varsigma _1^2\left( \begin{array}{{c}} \widetilde{\varphi }_{113}(\tau _k)\\ \widetilde{\varphi }_{114}(\tau _k)\\ \end{array} \right) +2\varsigma _1\varsigma _2\left( \begin{array}{{c}} \widetilde{\varphi }_{115}(\tau _k)\\ \widetilde{\varphi }_{116}(\tau _k)\\ \end{array} \right) +\varsigma _2^2\left( \begin{array}{{c}} \overline{\widetilde{\varphi }_{113}(\tau _k)}\\ \overline{\widetilde{\varphi }_{114}(\tau _k)}\\ \end{array} \right) \\ \end{array} \right) \\ \end{array} \right) . \end{aligned}$$

Secondly, from (5.41) we have

$$\begin{aligned} \mathcal {U}_{2,c}\left( \begin{array}{{c}} 0\\ \left( \begin{array}{{c}} \varsigma _1\\ \varsigma _2\\ \end{array} \right) \\ \end{array} \right) =\left( \begin{array}{{c}} 0_{\mathbb {R}}\\ \left( \begin{array}{{c}} 0_{\mathbb {R}^2}\\ \left( \begin{array}{{c}} \frac{1}{\omega _*i}(\ell _{20}\varsigma _1^2-\ell _{11}\varsigma _1\varsigma _2-\frac{1}{3}\ell _{02}\varsigma _2^2)\\ \frac{1}{\omega _*i}(\frac{1}{3}\overline{\ell }_{02}\varsigma _1^2 +\overline{\ell }_{11}\varsigma _1\varsigma _2-\overline{\ell }_{20}\varsigma _2^2)\\ \end{array} \right) \\ \end{array} \right) \\ \end{array} \right) . \end{aligned}$$

According to (5.20), we can obtain

$$\begin{aligned} {{(D^2\mathcal {H}(0_\mathcal {X})(\kappa _c,\mathcal {U}_2(\kappa _c)))_{\tilde{\tau }=0}}}=\tau _kD^2F(\overline{p}_{+})(\kappa _c,\mathcal {U}_2(\kappa _c)) =\left( \begin{array}{{c}} \left( \begin{array}{{c}} \tau _k\varphi _{1111}\\ \tau _k\varphi _{1112}\\ \end{array} \right) \\ 0_{L^1((0,+\infty ),\mathbb {R}^2)}\\ \end{array} \right) \end{aligned}$$

with

$$\begin{aligned} \varphi _1^1(a)&=(\varsigma _1\beta _1+\varsigma _2\beta _3)(a),~ \varphi _1^2(a)=(\varsigma _1\beta _2+\varsigma _2\beta _4)(a),\\ \varphi _2^1(a)&=\left( \frac{1}{\omega _*i}\left( \ell _{20}\varsigma _1^2-\ell _{11}\varsigma _1\varsigma _2 -\frac{1}{3}\ell _{02}\varsigma _2^2\right) \beta _1\right. \\&\quad +\frac{1}{\omega _*i}\left( \frac{1}{3}\overline{\ell }_{02}\varsigma _1^2 +\overline{\ell }_{11}\varsigma _1\varsigma _2-\overline{\ell }_{20}\varsigma _2^2\right) \beta _3\\&\quad +\varsigma _1^2\widetilde{\varphi }_{113}(\tau _k)+2\varsigma _1\varsigma _2\widetilde{\varphi }_{115}(\tau _k)+ \varsigma _2^2\overline{\widetilde{\varphi }_{113}(\tau _k)}\bigg )(a),\\ \varphi _2^2(a)&=\left( \frac{1}{\omega _*i}\left( \ell _{20}\varsigma _1^2-\ell _{11}\varsigma _1\varsigma _2 -\frac{1}{3}\ell _{02}\varsigma _2^2\right) \beta _2\right. \\&\quad +\frac{1}{\omega _*i}\left( \frac{1}{3}\overline{\ell }_{02}\varsigma _1^2 +\overline{\ell }_{11}\varsigma _1\varsigma _2-\overline{\ell }_{20}\varsigma _2^2\right) \beta _4\\&\quad +\varsigma _1^2\widetilde{\varphi }_{114}(\tau _k)+2\varsigma _1\varsigma _2\widetilde{\varphi }_{116}(\tau _k)+ \varsigma _2^2\overline{\widetilde{\varphi }_{114}(\tau _k)}\bigg )(a). \end{aligned}$$

Therefore, from (5.9) and (5.10), we have

5.43 $$\begin{aligned} {{\left( \Pi _c^{\mathcal {A}_{\tau _k}}D^2\mathcal {H}(0_\mathcal {X})(\kappa _c,\mathcal {U}_2(\kappa _c))\right) _{\tilde{\tau }=0}}}&=\tau _k\varphi _{1111} \Pi _c^{\mathcal {A}_{\tau _k}}\Theta _1+\tau _k\varphi _{1112} \Pi _c^{\mathcal {A}_{\tau _k}}\Theta _2\nonumber \\&=\tau _k\varphi _{1111}\left( \begin{array}{{c}} 0_{\mathbb {R}^2}\\ \varphi _1(a)\\ \end{array} \right) +\tau _k\varphi _{1112}\left( \begin{array}{{c}} 0_{\mathbb {R}^2}\\ \varphi _2(a)\\ \end{array} \right) . \end{aligned}$$

Then from (5.23) and (5.43), we get

$$\begin{aligned}&\left( \frac{1}{3!}\Pi _cD^3{{\mathcal {P}_2(0_\mathcal {X})}}(\kappa _c,\kappa _c,\kappa _c)\right) _{\tilde{\tau }=0} =\left( \Pi _cD^2{{\mathcal {P}(0_\mathcal {X})}}(\kappa _c,\mathcal {U}_2(\kappa _c))\right) _{\tilde{\tau }=0}\\&\qquad + \left( \frac{1}{3!}\Pi _cD^3{{\mathcal {P}(0_\mathcal {X})}}(\kappa _c,\kappa _c,\kappa _c)\right) _{\tilde{\tau }=0}\\&\quad =\left( \begin{array}{{c}} 0_{\mathbb {R}}\\ \left( \begin{array}{{c}} 0_{\mathbb {R}^2}\\ (\widehat{\overline{\varphi }}_3+\tau _k\varphi _{1111})\left( \begin{array}{{c}} 0_{\mathbb {R}^2}\\ \varphi _1(a)\\ \end{array} \right) +(\widehat{\overline{\varphi }}_4+\tau _k\varphi _{1112})\left( \begin{array}{{c}} 0_{\mathbb {R}^2}\\ \varphi _2(a)\\ \end{array} \right) \\ \end{array} \right) \\ \end{array} \right) .\\ \end{aligned}$$

Finally, we get the third-order terms of the normal form expressed in terms of the basis $\{\overline{\nu }_1,\overline{\nu }_2,\overline{\nu }_3\}$ as follows:

$$\begin{aligned}&\left( \frac{1}{3!}\Pi _cD^3{{\mathcal {P}_2(0_\mathcal {X})}}(\kappa _c,\kappa _c,\kappa _c)\right) _{\tilde{\tau }=0} =\left( \frac{1}{3!}\Pi _cD^3{{\mathcal {P}(0_\mathcal {X})}}(\kappa _c,\kappa _c,\kappa _c)\right) _{\tilde{\tau }=0}\\&\qquad - ((\mathcal {A}_c,\Pi _c\mathcal {U}_3)(\kappa _c))_{\tilde{\tau }=0}\\&\quad =(\overline{\nu }_1,\overline{\nu }_2,\overline{\nu }_3)\left( \begin{array}{{c}} 0_{\mathbb {R}}\\ \text{ Proj}_3^{\text{ K }}\left( \begin{array}{{c}} (\widehat{\overline{\varphi }}_3+\tau _k\varphi _{1111})\left( \begin{array}{{c}} 0_{\mathbb {R}^2}\\ \varphi _1(a)\\ \end{array} \right) \\ (\widehat{\overline{\varphi }}_4+\tau _k\varphi _{1112})\left( \begin{array}{{c}} 0_{\mathbb {R}^2}\\ \varphi _2(a)\\ \end{array} \right) \\ \end{array} \right) \\ \end{array} \right) +O(|(\varsigma _1,\varsigma _2)|\tilde{\tau }^2)\\&\quad =(\overline{\nu }_1,\overline{\nu }_2,\overline{\nu }_3)\left( \begin{array}{{c}} 0_{\mathbb {R}}\\ \left( \begin{array}{{c}} \Xi \varsigma _1^2\varsigma _2\\ \overline{\Xi }\varsigma _1\varsigma _2^2\\ \end{array} \right) \\ \end{array} \right) +O(|(\varsigma _1,\varsigma _2)|\tilde{\tau }^2),\\ \end{aligned}$$

where $\Xi =\left( \frac{\text{ d }~\text{ det }(\Delta (\omega _*i))}{\text{ d }\lambda }\right) ^{-1}\mathcal {Q}$ and the forms of $\mathcal {Q}$ is given in Appendix A.3. Thus, we get the normal form of the reduced system as follows:

5.44 $$\begin{aligned} \frac{\text{ d }}{\text{ d }t}\left( \begin{array}{{c}} \varsigma _1(t)\\ \varsigma _2(t)\\ \end{array} \right)&=\mathcal {M}_c\left( \begin{array}{{c}} \varsigma _1(t)\\ \varsigma _2(t)\\ \end{array} \right) +\left( \begin{array}{{c}} \left( \digamma _1+\frac{\overline{\digamma }_1}{2\omega _*}\right) \varsigma _1\tilde{\tau }\\ \left( \overline{\digamma }_1+\frac{\digamma _1}{2\omega _*}\right) \varsigma _2\tilde{\tau }\\ \end{array} \right) +\left( \begin{array}{{c}} \Xi \varsigma _1^2\varsigma _2\\ \overline{\Xi }\varsigma _1\varsigma _2^2\\ \end{array} \right) \nonumber \\&\quad +O(|(\varsigma _1,\varsigma _2)|\tilde{\tau }^2+|(\tilde{\tau },(\varsigma _1,\varsigma _2))|^4)). \end{aligned}$$

In order to discuss the direction of the Hopf bifurcation and the stability of the non-trivial periodic solutions in system (5.44), we use the change of variables:

5.45 $$\begin{aligned} {\varsigma }_{1}={\wp }\text{ cos }\chi -i{\wp }\text{ sin }\chi ,\;{\wp }>0,\quad \textrm{and}\quad {\varsigma }_{2}={\wp }\text{ cos }\chi +i{\wp }\text{ sin }\chi ,\;{\wp }>0, \end{aligned}$$

then the normal form can be rewritten as

5.46 $$\begin{aligned} \frac{\text{ d }{\wp }}{\text{ d }t}&=\mathcal {T}_{1}{\tilde{\tau }}{\wp }+\mathcal {T}_{2}{\wp }^3+O({\wp }{\tilde{\tau }}^{2}+|(\tilde{\tau },{\wp })|^{4}),\nonumber \\ \frac{\text{ d }\chi }{\text{ d }t}&=-\sigma _{k}+O(|({\tilde{\tau }},{\wp })|) \end{aligned}$$

with

$$\begin{aligned} \mathcal {T}_{1}=\text{ Re }(\digamma _{1}),~\mathcal {T}_{2}=\text{ Re }(\Xi ). \end{aligned}$$

From Chow and Hale (1982, Chapter 9, Theorems 5.2, 5.5 and 5.6), we know that the sign of $\mathcal {T}_1\mathcal {T}_2$ determines the direction of the Hopf bifurcation and the sign of $\mathcal {T}_2$ determines the stability of the bifurcating periodic solutions. Then we have the following theorem.

#### Theorem 5.2

The flow of system (1.5) on the center manifold of the positive equilibrium near $\tau =\tau _k,k=0,1,2,\ldots$ is given by (5.46). Then we have the following results:

(i) if $\mathcal {T}_1\mathcal {T}_2<0$, then the Hopf bifurcation is supercritical; on the contrary, if $\mathcal {T}_1\mathcal {T}_2>0$ it is subcritical;
(ii) the bifurcating periodic solutions are stable if $\mathcal {T}_2<0$ and unstable if $\mathcal {T}_2>0$.

## Numerical simulations

In this section, we give some numerical simulations of system (1.5) to illustrate our theoretical results in Theorem 4.2. Firstly, we select some parameters values as follow: $\mu =0.5,\;r=\Lambda -d=1.8,\;\Lambda =2,\;d=0.2,\;K=30,\;b=3.5,\;m=0.01,\;\alpha =2,\;s=0.3,\;\eta =2,\;M=0.1.$ The initial conditions are selected as $u(0,a)=75.7081e^{-1.5a}$ and $V(0)=7.2888$. Then the new maturation function $\beta (a)$ and new fertility function *f*(*a*) become

$$\begin{aligned} \beta (a)= \left\{ \begin{array}{ll} 0 &{}\quad \text {if}\;a\in (0,\tau ),\\ 0.5e^{0.5\tau }&{} \quad \text {if}\;a\ge \tau \end{array} \right. \quad \text{ and }\quad f(a)= \left\{ \begin{array}{ll} 0 &{}\quad \text {if}\; a\in (0,\tau ),\\ 1.75e^{0.5\tau } &{}\quad \text {if}\;a\ge \tau . \end{array} \right. \end{aligned}$$

By using MATLAB to calculate the relevant conditions, we have $b\eta =7>1,\mu P-s b\eta r=-0.81<0,\Delta =948.8340>0,\sqrt{\Delta }-K(\mu P-sb\eta r)=55.1031>0,P\sqrt{\Delta }-PK(\mu P-sb\eta r)-2sb\eta r\alpha = 172.6621>0$ and $M=0.1<\frac{\mu \alpha }{sb\eta }=0.4762$, so all conditions of Assumptions 1.1 and 3.1 holds. It implies that conditions in Theorem 4.2 are satisfied and the positive equilibrium of system (1.5) exists.

**Fig. 1:**
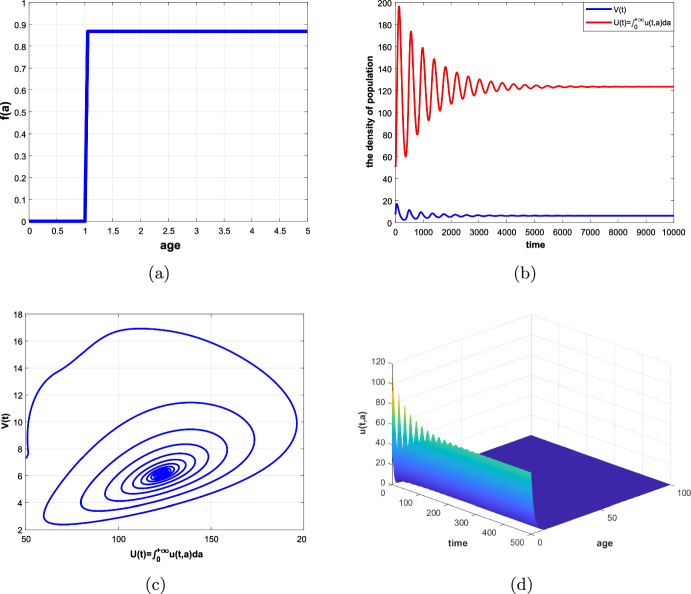
Numerical solutions of system (1.5) when $\tau =1.1<\hat{\tau }_0=1.2003$: **a** image of the function *f*(*a*) with age *a*; **b** solution structure of prey and predator populations; **c** phase portrait of system (1.5); **d** distribution function of predator *u*(*t*, *a*)

Secondly, in order to obtain the critical value for Hopf bifurcation, further calculation yields that $\omega _0=0.9492$ and $\hat{\tau }_0=1.2003$. In Fig. 1, we select the bifurcation parameter $\tau =1.1<\hat{\tau }_0=1.2003.$ Then the positive equilibrium $(\overline{u}_{\tau =1.1}(a),\overline{V})=(75.7081e^{-0.55a},7.2888)$ of system (1.5) is locally asymptotically stable. Figure 1a indicates that the fertility function *f*(*a*) varies with age *a*. Figure 1b shows that the solutions of prey and predator populations are stable. Figure 1c demonstrates the phase diagram between prey population *V*(*t*) and predator population $\int _{0}^{+\infty }u(t,a)\text{ d }a$ trajectories of system (1.5). The change of the distribution function of predator *u*(*t*, *a*) as the time and age vary is shown in Fig. 1d.

**Fig. 2:**
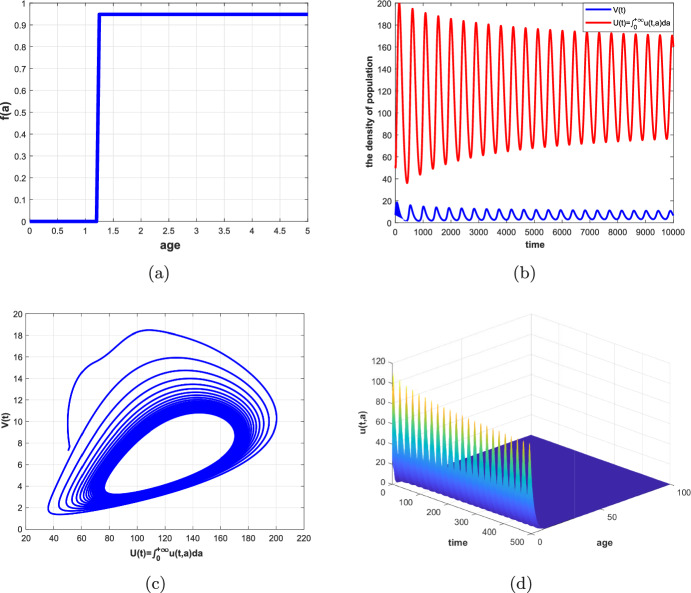
Numerical solutions of system (1.5) when $\tau =1.28>\hat{\tau }_0=1.2003$: **a** image of the function *f*(*a*) with age *a*; **b** periodic structure of prey and predator populations; **c** phase portrait of system (1.5); **d** distribution function of predator *u*(*t*, *a*)

In Fig. 2, we select the bifurcation parameter $\tau =1.28>\hat{\tau }_0=1.2003$ and the positive equilibrium $(\overline{u}_{\tau =1.28}(a),\overline{V})=(88.0967e^{-0.64a},7.2888)$ of system (1.5) is unstable, it shows that the sustained periodic oscillation behavior is generated near the positive equilibrium $(\overline{u}_{\tau =1.28}(a),\overline{V})=(88.0967e^{-0.64a},7.2888)$ of system (1.5); that is, the periodic solution occurs. Figure 2a indicates that the fertility function *f*(*a*) varies with age *a*. Figure 2b indicates that the solution curves exhibit sustained periodic oscillation behavior of prey and predator populations at $\tau =1.28>\hat{\tau }_0=1.2003$, respectively. Figure 2c represents the phase trajectories of prey population *V*(*t*) and predator population $\int _{0}^{+\infty }u(t,a)\text{ d }a$ which tend to a stable limit cycle. The change of the distribution function of predator *u*(*t*, *a*) as the time and age vary is given in Fig. 2d.

**Fig. 3:**
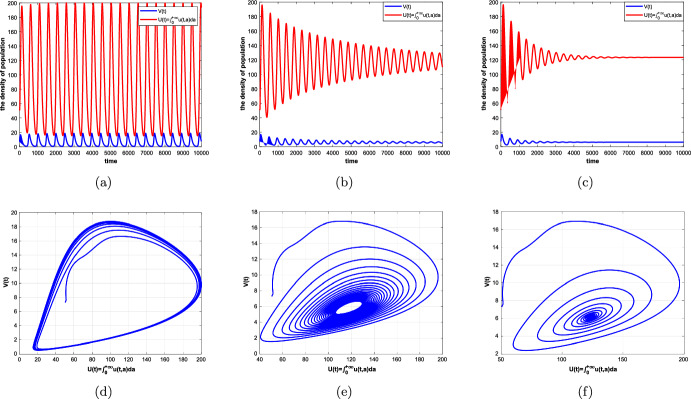
When $\tau =1.1$, the effect of the coefficient of the interference among predators *s* on the dynamics of the prey and predator populations: **a, d** $s=0.29$; **b, e** $s=0.295$; **c, f** $s=0.3$

**Fig. 4:**
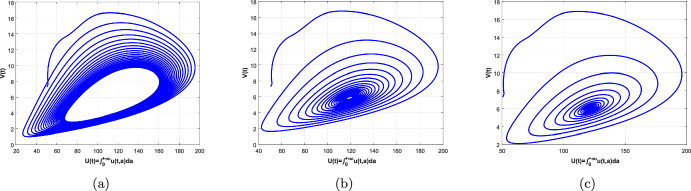
When $\tau =1.1$, the effect of the half saturation constant $\alpha$ on the dynamics of the prey and predator populations: **a** $\alpha =1.2$; **b** $\alpha =1.5$; **c** $\alpha =1.8$

Thirdly, due to the fact that B–D functional response could describe the interference among predators, we select *s* as the sensitivity parameter, and its effect on the stability of the population dynamics of system (1.5) is shown in Fig. 3. It can be seen from Fig. 3 that system (1.5) changes from instability to stability at the positive equilibrium and the stable limit cycle disappears as the value of *s* increases gradually, the positive equilibrium changes from $(\overline{u}_{\tau =1.1}(a),\overline{V})=(70.6299e^{-0.55a},6.9063)$ to $(\overline{u}_{\tau =1.1}(a),\overline{V})=(75.7081e^{-0.55a},7.2888)$ as *s* increases from 0.29 to 0.3, which indicates that with the increase of *s*, the density of predators will decrease and the density of prey will increase, which also indirectly leads to the balance of system (1.5). Meanwhile, compared with the B–D functional response and Holling-II type functional response $(s=0)$ used by Yang (2019); Yang and Wang (2020a), from Fig. 3, we can obtain that system (1.5) changes from instability to stability at the positive equilibrium and the stable limit cycle disappears as the value of *s* increases gradually, which indicates that the system (1.5) loses stability at the positive equilibrium as the value of *s* decreases or $s\rightarrow 0$ eventually. Similarly, the B–D functional response can be degenerated into an Michaelis–Menten type functional response $(\alpha =0)$ used by Zhang and Liu (2019, 2021), it can be seen from Fig. 4 that system (1.5) loses stability at the positive equilibrium as the value of $\alpha$ decreases or $\alpha \rightarrow 0$ eventually, it is more practical to consider the B–D functional response, the degree of interference *s* between predators and the half saturation constant $\alpha$ plays a key role in the stability of the system, which is a meaningful result.

**Fig. 5:**
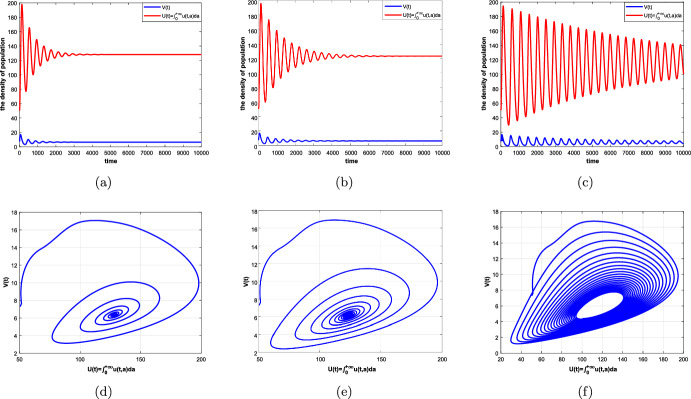
When $\tau =1.1$, the effect of the constant harvesting rate *M* on the dynamics of the prey and predator populations: **a, d** $M=0$; **b, e** $M=0.1$; **c, f** $M=0.2$

Fourthly, Fig. 5 shows the effect of the constant harvesting rate *M* on the dynamic behavior of system (1.5). From Fig. 5 we can see that system (1.5) changes from stability to instability at the positive equilibrium as the constant harvesting rate *M* increases, which indicates that humans should capture within a reasonable harvesting threshold range and should not over capture for their own benefit in nature, as this can lead to a loss of balance in the biological population system.

**Fig. 6:**
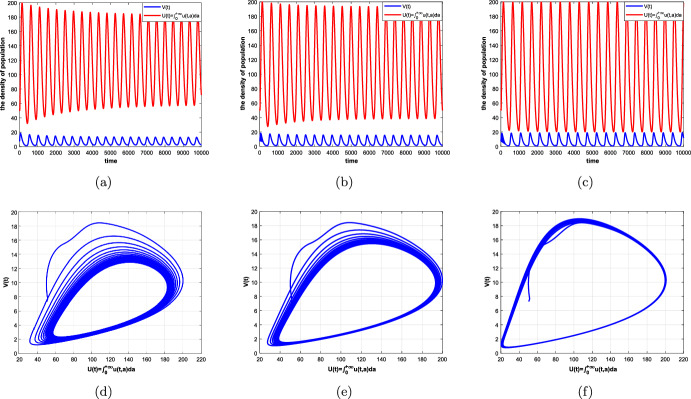
When $\tau =1.28$, the effect of the constant harvesting rate *M* on the dynamics of the prey and predator populations: **a, d** $M=0.11$; **b, e** $M=0.12$; **c, f** $M=0.13$

**Fig. 7:**
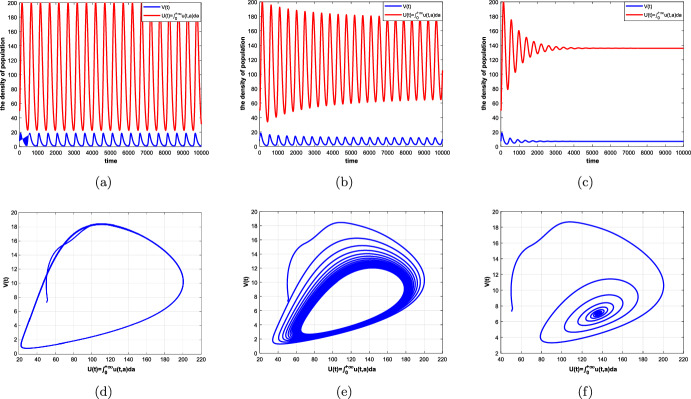
When $\tau =1.28$, the effect of the number of prey refuge *m* on the dynamics of the prey and predator populations: **a, d** $m=0.0029$; **b, e** $m=0.0089$; **c, f** $m=0.04$

Finally, in order to discuss the direction and stability of the Hopf bifurcation, by fixing $\tau =1.28$, which is the value of the Hopf bifurcation and the periodic solution appears at the positive equilibrium, we can obtain that the stability of system (1.5) changes from a stable equilibrium to a stable limit cycle to an unstable limit cycle as the values of parameters $b,\eta ,M,K$ and *d* increase, and the Hopf bifurcation is supercritical (see Fig. 6 using *M* as an example). On the contrary, the Hopf bifurcation is subcritical as the values of the parameters $m,s,\alpha$ and *r* increase, the stability of system (1.5) changes from a unstable limit cycle to a stable limit cycle to a stable equilibrium (see Fig. 7 using *m* as an example).

## Discussion

In this article, an age-structured predator–prey system with B–D functional response and prey harvesting was proposed. By converting the system into a non-densely defined abstract Cauchy problem, we studied the existence and stability of the equilibria of the non-densely defined Cauchy problem. Taking the maturation period $\tau$ as a bifurcation parameter, the existence of Hopf bifurcation and stability of the bifurcation periodic solutions at the positive equilibrium of system (1.5) were obtained. We found that system (1.5) is locally asymptotically stable if $\tau <\hat{\tau }_0$ and exhibits periodic oscillation phenomenon if $\tau >\hat{\tau }_0$, which implies that the maturation period $\tau$ has a great influence for the stability of system (1.5). We also provided a detailed study on the properties of Hopf bifurcation. According to the theoretical analysis, the Hopf bifurcation is supercritical if $\mathcal {T}_1\mathcal {T}_2<0$ and subcritical if $\mathcal {T}_1\mathcal {T}_2>0$; and the bifurcating periodic solutions are stable if $\mathcal {T}_2<0$ and unstable if $\mathcal {T}_2>0$. In addition, it is found that the degree of interference *s* between predators also plays a decisive role in the stability of system, and adjusting the value of the constant harvesting rate *M* can also change the stability of the system.

By numerical simulation methods we found that the Hopf bifurcation is supercritical as the values of parameters $b,\eta ,M,K$ and *d* increase, and the Hopf bifurcation is subcritical as the values of the parameters $m,s,\alpha$ and *r* increase. Compared with the results of Tripathi et al. (2015), we considered the influence of age structure on the predator population, which allows us to observe the changes of the dynamics behaviors caused by the age structure. The maturation period and reproductive ability of the predator population can also be expressed, and the maturation period $\tau$ also determines the stability of system.

There are more questions for the age structure model that deserve further consideration. For example, if coexistence equilibria are unstable in Sect. 4, it is interesting to study whether the coexistence equilibria would become stable with the increase of the maturation period $\tau$ and whether Hopf bifurcation would occur. Moreover, it is challenging to study the impact of spatial diffusion on the stability of the system. We leave these for future investigation.

## Data Availability

No data was used and no new data was generated in this study.

## Appendix A.1

In this part, we provide the expressions of $\widehat{\overline{\varphi }}_1(\tau _k,\tilde{\tau }),~\widehat{\overline{\varphi }}_2(\tau _k,\tilde{\tau }),~\widehat{\overline{\overline{\varphi }}}_1(\tau _k,\tilde{\tau }),~ \widehat{\overline{\overline{\varphi }}}_2(\tau _k,\tilde{\tau }),\varphi _{2211},~\varphi _{2212}$, $\widehat{\overline{\varphi }}_3(\tau _k,\tilde{\tau })$ and $\widehat{\overline{\varphi }}_4(\tau _k,\tilde{\tau })$ that were used in Sect. 5.3.

$$\begin{aligned} \widehat{\overline{\varphi }}_1(\tau _k,\tilde{\tau })&=-\tilde{\tau }\frac{\mu P_1}{b\eta m_1\nu } \int _{0}^{+\infty }\overline{\varphi }^1(a)\text{ d }a+\tilde{\tau }\frac{1}{b}\int _{0}^{+\infty }f(a)\overline{\varphi }^1(a)\text{ d }a\\&\quad +\tilde{\tau }\frac{\mu PP_1}{sb\eta m_1\nu }\int _{0}^{+\infty }\overline{\varphi }^2(a)\text{ d }a+\tau _k\frac{s\mu P_1}{(b\eta \nu m_1)^2} \left( \int _{0}^{+\infty }\overline{\varphi }^1(a)\text{ d }a\right) ^2\\&\quad +\tau _k\frac{\mu (2-b\eta )P_1}{b^2\eta ^2\nu ^2m_1}\int _{0}^{+\infty }\overline{\varphi }^1(a)\text{ d }a \int _{0}^{+\infty }\overline{\varphi }^2(a)\text{ d }a\\&\quad -\frac{\tau _ks}{b^2\eta \nu m_1 }\int _{0}^{+\infty }f(a)\overline{\varphi }^1(a)\text{ d }a\int _{0}^{+\infty }\overline{\varphi }^1(a)\text{ d }a\\&\quad -\tau _k\frac{\mu PP_1}{sb^2\eta ^2\nu ^2m_1} \left( \int _{0}^{+\infty }\overline{\varphi }^2(a)\text{ d }a\right) ^2\\&\quad +\frac{\tau _k(b\eta -1)}{b^2\eta \nu }\int _{0}^{+\infty }f(a)\overline{\varphi }^1(a)\text{ d }a\int _{0}^{+\infty }\overline{\varphi }^2(a)\text{ d }a\\&\quad +\tilde{\tau }\frac{2\mu P_1^2(1-\tau _k\mu )}{(b\eta m_1\nu )^2}\int _{0}^{+\infty }\overline{\varphi }^1(a)\text{ d }a\\&\quad +\tau _k\frac{\mu (2-b\eta )P_1}{b^2\eta ^2\nu ^2m_1}\int _{0}^{+\infty }\overline{\varphi }^2(a)\text{ d }a \int _{0}^{+\infty }\overline{\varphi }^1(a)\text{ d }a\\&\quad -\tilde{\tau }\frac{P_1(1-\tau _k\mu )}{b^2\eta m_1\nu }\int _{0}^{+\infty }f(a)\overline{\varphi }^1(a)\text{ d }a\\&\quad +\tilde{\tau }\frac{\mu (2-b\eta )P_1(1-\tau _kd)}{b^2\eta ^2\nu m_1}\int _{0}^{+\infty }\overline{\varphi }^1(a)\text{ d }a\\&\quad +\tilde{\tau }\frac{(b\eta -1)(1-\tau _kd)}{b^2\eta }\int _{0}^{+\infty }f(a)\overline{\varphi }^1(a)\text{ d }a\\&\quad +\tilde{\tau }\frac{\mu (2-b\eta )P_1^2(1-\tau _k\mu )}{sb^2\eta ^2\nu ^2m_1}\int _{0}^{+\infty }\overline{\varphi }^2(a)\text{ d }a\\&\quad -\tilde{\tau }\frac{\mu PP_1(1-\tau _kd)}{sb^2\eta ^2\nu m_1}\int _{0}^{+\infty }\overline{\varphi }^2(a)\text{ d }a, \end{aligned}$$

$$\begin{aligned} \widehat{\overline{\varphi }}_2(\tau _k,\tilde{\tau })&=\tilde{\tau }\frac{-2srb^2\eta ^2\nu ^2+[rKsb^2\eta ^2-\mu K(b\eta -1)P]\nu +\mu K(b\eta -1)\alpha }{K\eta ^2\nu bs}\\&\quad \times \int _{0}^{+\infty }\overline{\varphi }^2(a)\text{ d }a-\tilde{\tau }\frac{\mu P_1}{b^2\eta ^2m_1\nu } \int _{0}^{+\infty }\overline{\varphi }^1(a)\text{ d }a\\&\quad +\frac{\tau _k\mu (b\eta -2)P_1}{b^3\eta ^3\nu ^2m_1}\int _{0}^{+\infty }\overline{\varphi }^1(a)\text{ d }a \int _{0}^{+\infty }\overline{\varphi }^2(a)\text{ d }a\\&\quad -\tilde{\tau }\frac{1}{b\eta }\int _{0}^{+\infty }\beta (a)\overline{\varphi }^1(a)\text{ d }a +\frac{\tau _ks}{b^2\eta ^2\nu m_1}\\&\quad \times \int _{0}^{+\infty }\beta (a)\overline{\varphi }^1(a)\text{ d }a\int _{0}^{+\infty }\overline{\varphi }^1(a)\text{ d }a\\&\quad -\frac{\tau _k(b\eta -1)}{b^2\eta ^2\nu }\int _{0}^{+\infty }\beta (a)\overline{\varphi }^1(a)\text{ d }a\int _{0}^{+\infty }\overline{\varphi }^2(a)\text{ d }a\\&\quad -\frac{2\tau _kr}{K}\left( \int _{0}^{+\infty }\overline{\varphi }^2(a)\text{ d }a\right) ^2\\&\quad +\frac{2\tau _k\mu (b\eta -1)P_1}{sb^3\eta ^3\nu ^2m_1} \left( \int _{0}^{+\infty }\overline{\varphi }^2(a)\text{ d }a\right) ^2\\&\quad -\tilde{\tau }\frac{\mu (2-b\eta )P_1^2(1-\tau _k\mu )}{b^3\eta ^3\nu ^2m_1}\int _{0}^{\infty }\overline{\varphi }^2(a)\text{ d }a\\&\quad -\tau _k\frac{\mu (2-b\eta )P_1}{b^3\eta ^3\nu ^2m_1}\int _{0}^{+\infty }\overline{\varphi }^2(a)\text{ d }a \int _{0}^{\infty }\overline{\varphi }^1(a)\text{ d }a\\&\quad +\tilde{\tau }\frac{2\mu P_1^2(1-\tau _k\mu )}{b^3\eta ^3m_1^2\nu ^2} \int _{0}^{\infty }\overline{\varphi }^1(a)\text{ d }a\\&\quad +\tilde{\tau }\frac{\mu (b\eta -2)P_1(1-\tau _kd)}{b^3\eta ^3\nu m_1}\int _{0}^{+\infty }\overline{\varphi }^1(a)\text{ d }a\\&\quad +\tilde{\tau }\frac{P_1(1-\tau _k\mu )}{b^2\eta ^2m_1\nu }\int _{0}^{+\infty }\beta (a)\overline{\varphi }^1(a)\text{ d }a\\&\quad +\tilde{\tau }\frac{2[K \mu (b\eta -1)P_1- s r b^3\eta ^3\nu ^2m_1](1-\tau _kd)}{Ksb^3\eta ^3\nu m_1}\int _{0}^{+\infty }\overline{\varphi }^2(a)\text{ d }a\\&\quad -\tilde{\tau }\frac{(b\eta -1)(1-\tau _kd)}{b^2\eta ^2\nu }\int _{0}^{+\infty }\beta (a)\overline{\varphi }^1(a)\text{ d }a, \end{aligned}$$

$$\begin{aligned} \widehat{\overline{\overline{\varphi }}}_1(\tau _k,\tilde{\tau })&=-\tilde{\tau }\frac{\mu P_1}{b\eta m_1\nu } \int _{0}^{+\infty }\overline{\overline{\varphi }}^1(a)\text{ d }a+\tilde{\tau }\frac{1}{b} \int _{0}^{+\infty }f(a)\overline{\overline{\varphi }}^1(a)\text{ d }a\\&\quad +\tilde{\tau }\frac{\mu PP_1}{sb\eta m_1\nu }\int _{0}^{+\infty }\overline{\overline{\varphi }}^2(a)\text{ d }a\\&\quad +\tau _k\frac{s\mu P_1}{(b\eta \nu m_1)^2} \left( \int _{0}^{+\infty }\overline{\overline{\varphi }}^1(a)\text{ d }a\right) ^2\\&\quad +\tau _k\frac{\mu (2-b\eta )P_1}{b^2\eta ^2\nu ^2m_1}\int _{0}^{\infty }\overline{\overline{\varphi }}^1(a)\text{ d }a \int _{0}^{\infty }\overline{\overline{\varphi }}^2(a)\text{ d }a\\&\quad -\frac{\tau _ks}{b^2\eta \nu m_1}\int _{0}^{+\infty }f(a)\overline{\overline{\varphi }}^1(a)\text{ d }a\int _{0}^{+\infty }\overline{\overline{\varphi }}^1(a)\text{ d }a\\&\quad -\tau _k\frac{\mu PP_1}{sb^2\eta ^2\nu ^2m_1} \left( \int _{0}^{+\infty }\overline{\overline{\varphi }}^2(a)\text{ d }a\right) ^2\\&\quad +\frac{\tau _k(b\eta -1)}{b^2\eta \nu }\int _{0}^{+\infty }f(a)\overline{\overline{\varphi }}^1(a)\text{ d }a\int _{0}^{+\infty }\overline{\overline{\varphi }}^2(a)\text{ d }a\\&\quad +\tau _k\frac{\mu (2-b\eta )P_1}{b^2\eta ^2\nu ^2m_1}\int _{0}^{+\infty }\overline{\overline{\varphi }}^2(a)\text{ d }a \int _{0}^{+\infty }\overline{\overline{\varphi }}^1(a)\text{ d }a\\&\quad +\tilde{\tau }\frac{2\mu P_1^2(1-\tau _k\mu )}{(b\eta m_1\nu )^2} \int _{0}^{+\infty }\overline{\overline{\varphi }}^1(a)\text{ d }a\\&\quad +\tilde{\tau }\frac{\mu (2-b\eta )P_1(1-\tau _kd)}{b^2\eta ^2\nu m_1}\int _{0}^{+\infty }\overline{\overline{\varphi }}^1(a)\text{ d }a\\&\quad -\tilde{\tau }\frac{P_1(1-\tau _k\mu )}{b^2\eta m_1\nu }\int _{0}^{+\infty }f(a)\overline{\overline{\varphi }}^1(a)\text{ d }a\\&\quad +\tilde{\tau }\frac{(b\eta -1)(1-\tau _kd)}{b^2\eta }\int _{0}^{+\infty }f(a)\overline{\overline{\varphi }}^1(a)\text{ d }a\\&\quad +\tilde{\tau }\frac{\mu (2-b\eta )P_1^2(1-\tau _k\mu )}{sb^2\eta ^2\nu ^2m_1}\int _{0}^{+\infty }\overline{\overline{\varphi }}^2(a)\text{ d }a\\&\quad -\tilde{\tau }\frac{\mu PP_1(1-\tau _kd)}{sb^2\eta ^2\nu m_1}\int _{0}^{+\infty }\overline{\overline{\varphi }}^2(a)\text{ d }a, \end{aligned}$$

$$\begin{aligned} \widehat{\overline{\overline{\varphi }}}_2(\tau _k,\tilde{\tau })&=\tilde{\tau }\frac{-2srb^2\eta ^2\nu ^2+[r Ksb^2\eta ^2-\mu K(b\eta -1)P]\nu +\mu K(b\eta -1)\alpha }{K\eta ^2\nu bs}\nonumber \\&\quad \times \int _{0}^{+\infty }\overline{\overline{\varphi }}^2(a)\text{ d }a\\&\quad -\tilde{\tau }\frac{\mu P_1}{b^2\eta ^2m_1\nu } \int _{0}^{+\infty }\overline{\overline{\varphi }}^1(a)\text{ d }a-\tilde{\tau }\frac{1}{b\eta }\int _{0}^{+\infty }\beta (a)\overline{\overline{\varphi }}^1(a)\text{ d }a\\&\quad -\frac{\tau _k\mu sP_1}{b^3\eta ^3\nu ^2m_1^2} \left( \int _{0}^{+\infty }\overline{\overline{\varphi }}^1(a)\text{ d }a\right) ^2\\&\quad +\frac{\tau _k\mu (b\eta -2)P_1}{b^3\eta ^3\nu ^2m_1}\int _{0}^{+\infty }\overline{\overline{\varphi }}^1(a)\text{ d }a \int _{0}^{\infty }\overline{\overline{\varphi }}^2(a)\text{ d }a\\&\quad +\frac{\tau _ks}{b^2\eta ^2\nu m_1}\int _{0}^{+\infty }\beta (a)\overline{\overline{\varphi }}^1(a)\text{ d }a\int _{0}^{\infty }\overline{\overline{\varphi }}^1(a)\text{ d }a\\&\quad -\frac{\tau _k(b\eta -1)}{b^2\eta ^2\nu }\int _{0}^{+\infty }\beta (a)\overline{\overline{\varphi }}^1(a)\text{ d }a\\&\quad \times \int _{0}^{+\infty }\overline{\overline{\varphi }}^2(a)\text{ d }a +\tau _k\frac{2[K \mu (b\eta -1)P_1- s r b^3\eta ^3\nu ^2m_1]}{Ksb^3\eta ^3\nu ^2m_1}\\&\quad \times \left( \int _{0}^{+\infty }\overline{\overline{\varphi }}^2(a)\text{ d }a\right) ^2\\&\quad -\tau _k\frac{\mu (2-b\eta )P_1}{b^3\eta ^3\nu ^2m_1}\int _{0}^{+\infty }\overline{\overline{\varphi }}^2(a)\text{ d }a \int _{0}^{+\infty }\overline{\overline{\varphi }}^1(a)\text{ d }a\\&\quad -\tilde{\tau }\frac{2r\nu (1-\tau _kd)}{K}\int _{0}^{\infty }\overline{\overline{\varphi }}^2(a)\text{ d }a \\&\quad +\tilde{\tau }\frac{2\mu P_1^2(1-\tau _k\mu )}{b^3\eta ^3m_1^2\nu ^2} \int _{0}^{+\infty }\overline{\overline{\varphi }}^1(a)\text{ d }a\\&\quad +\tilde{\tau }\frac{\mu (b\eta -2)P_1(1-\tau _kd)}{b^3\eta ^3\nu m_1}\int _{0}^{+\infty }\overline{\overline{\varphi }}^1(a)\text{ d }a\\&\quad +\tilde{\tau }\left[ \frac{2\mu (b\eta -1)P_1(1-\tau _kd)}{sb^3\eta ^3\nu m_1}-\frac{\mu (2-b\eta )P_1^2(1-\tau _k\mu )}{b^3\eta ^3\nu ^2m_1}\right] \int _{0}^{+\infty }\overline{\overline{\varphi }}^2(a)\text{ d }a\\&\quad +\tilde{\tau }\frac{P_1(1-\tau _k\mu )}{b^2\eta ^2m_1\nu }\int _{0}^{+\infty }\beta (a)\overline{\overline{\varphi }}^1(a)\text{ d }a\\&\quad -\tilde{\tau }\frac{(b\eta -1)(1-\tau _kd)}{b^2\eta ^2\nu }\int _{0}^{+\infty }\beta (a)\overline{\overline{\varphi }}^1(a)\text{ d }a, \end{aligned}$$

$$\begin{aligned} \varphi _{2211}&=\frac{2\mu (2b\eta -3)P_1}{b^3\eta ^3m_1\nu ^3}\Bigg ( \int _{0}^{+\infty }\varphi _1^1(a)\text{ d }a\int _{0}^{+\infty }\varphi _2^2(a)\text{ d }a\int _{0}^{+\infty }\varphi _3^2(a)\text{ d }a\\&\quad +\int _{0}^{+\infty }\varphi _1^2(a)\text{ d }a\int _{0}^{+\infty }\varphi _2^1(a)\text{ d }a\\&\quad \times \int _{0}^{+\infty }\varphi _3^2(a)\text{ d }a +\int _{0}^{+\infty }\varphi _1^2(a)\text{ d }a\int _{0}^{+\infty }\varphi _2^2(a)\text{ d }a\int _{0}^{+\infty }\varphi _3^1(a)\text{ d }a\Bigg )\\&\quad +\frac{2s\mu (b\eta -3)P_1}{b^3\eta ^3m_1^2\nu ^3}\Bigg ( \int _{0}^{+\infty }\varphi _1^1(a)\text{ d }a\int _{0}^{+\infty }\varphi _2^2(a)\text{ d }a\int _{0}^{+\infty }\varphi _3^1(a)\text{ d }a\\&\quad +\int _{0}^{+\infty }\varphi _1^1(a)\text{ d }a\int _{0}^{+\infty }\varphi _2^1(a)\text{ d }a\int _{0}^{+\infty }\varphi _3^2(a)\text{ d }a\Bigg )\\&\quad -\frac{s(b\eta -2)}{b^3\eta ^2m_1\nu ^2} \Bigg (\int _{0}^{+\infty }\varphi _1^1(a)\text{ d }a\int _{0}^{+\infty }\varphi _2^2(a)\text{ d }a\int _{0}^{+\infty }f(a)\varphi _3^1(a)\text{ d }a\\&\quad +\int _{0}^{+\infty }\varphi _1^1(a)\text{ d }a\\&\quad \times \int _{0}^{+\infty }f(a)\varphi _2^1(a)\text{ d }a\int _{0}^{+\infty }\varphi _3^2(a)\text{ d }a +\int _{0}^{+\infty }f(a)\varphi _1^1(a)\text{ d }a\int _{0}^{+\infty }\varphi _2^1(a)\text{ d }a\\&\quad \times \int _{0}^{+\infty }\varphi _3^2(a)\text{ d }a+\int _{0}^{+\infty }f(a)\varphi _1^1(a)\text{ d }a\int _{0}^{+\infty }\varphi _2^2(a)\text{ d }a\int _{0}^{+\infty }\varphi _3^1(a)\text{ d }a\\&\quad +\int _{0}^{+\infty }\varphi _1^2(a)\text{ d }a\int _{0}^{+\infty }\varphi _2^1(a)\text{ d }a\int _{0}^{+\infty }f(a)\varphi _3^1(a)\text{ d }a\Bigg )\\&\quad +\frac{2s^2}{b^3(\eta m_1\nu )^2} \Bigg (\int _{0}^{+\infty }\varphi _1^1(a)\text{ d }a\int _{0}^{+\infty }\varphi _2^1(a)\text{ d }a\int _{0}^{+\infty }f(a)\varphi _3^1(a)\text{ d }a\\&\quad +\int _{0}^{+\infty }\varphi _1^1(a)\text{ d }a\\&\quad \times \int _{0}^{+\infty }f(a)\varphi _2^1(a)\text{ d }a\int _{0}^{+\infty }\varphi _3^1(a)\text{ d }a +\int _{0}^{+\infty }f(a)\varphi _1^1(a)\text{ d }a\int _{0}^{+\infty }\varphi _2^1(a)\text{ d }a\\&\quad \times \int _{0}^{+\infty }\varphi _3^1(a)\text{ d }a\Bigg )-\frac{2(b\eta -1)}{b^3\eta ^2\nu ^2} \left( \int _{0}^{+\infty }f(a)\varphi _1^1(a)\text{ d }a\int _{0}^{+\infty }\varphi _2^2(a)\text{ d }a\right. \\&\left. \quad \times \int _{0}^{+\infty }\varphi _3^2(a)\text{ d }a+\int _{0}^{+\infty }\varphi _1^2(a)\text{ d }a\int _{0}^{+\infty }f(a)\varphi _2^2(a)\text{ d }a\int _{0}^{+\infty }f(a)\varphi _3^1(a)\text{ d }a\right) \\&\quad -\frac{6s^2\mu P_1}{(b\eta m_1\nu )^3} \left( \int _{0}^{+\infty }\varphi _1^1(a)\text{ d }a\int _{0}^{+\infty }\varphi _2^1(a)\text{ d }a\int _{0}^{+\infty }\varphi _3^1(a)\text{ d }a\right) \\&\quad -\frac{2s\mu (2b\eta -3)P_1}{b^3\eta ^3m_1^2\nu ^3} \left( \int _{0}^{+\infty }\varphi _1^2(a)\text{ d }a\int _{0}^{+\infty }\varphi _2^1(a)\text{ d }a\int _{0}^{+\infty }\varphi _3^1(a)\text{ d }a\right) \\&\quad -\frac{6\mu (b\eta -1)P_1}{s b^3\eta ^3\nu ^3} \left( \int _{0}^{+\infty }\varphi _1^2(a)\text{ d }a\int _{0}^{+\infty }\varphi _2^2(a)\text{ d }a\int _{0}^{+\infty }\varphi _3^2(a)\text{ d }a\right) , \end{aligned}$$

$$\begin{aligned} \varphi _{2212}&=\frac{2\mu (3-b\eta )P_1}{b^4\eta ^4m_1^2\nu ^3} \left( \int _{0}^{+\infty }\varphi _1^1(a)\text{ d }a\int _{0}^{+\infty }\varphi _2^1(a)\text{ d }a\int _{0}^{+\infty }\varphi _3^2(a)\text{ d }a\right. \\&\left. \quad +\int _{0}^{+\infty }\varphi _1^1(a)\text{ d }a\times \int _{0}^{+\infty }\varphi _2^2(a)\text{ d }a\int _{0}^{+\infty }\varphi _3^1(a)\text{ d }a\right) \\&\quad -\frac{2s^2}{b^3\eta ^3m_1^2\nu ^3}\Bigg ( \int _{0}^{+\infty }\varphi _1^1(a)\text{ d }a\int _{0}^{+\infty }\varphi _2^1(a)\text{ d }a\\&\quad \times \int _{0}^{+\infty }\beta (a)\varphi _3^1(a)\text{ d }a +\int _{0}^{+\infty }\beta (a)\varphi _1^1(a)\text{ d }a\int _{0}^{+\infty }\varphi _2^1(a)\text{ d }a\int _{0}^{+\infty }\varphi _3^1(a)\text{ d }a\\&\quad +\int _{0}^{+\infty }\varphi _1^1(a)\text{ d }a\int _{0}^{+\infty }\beta (a)\varphi _2^1(a)\text{ d }a\int _{0}^{+\infty }\varphi _3^1(a)\text{ d }a\Bigg ) +\frac{2\mu (3-2b\eta )P_1}{b^4\eta ^4m_1^2\nu ^3}\\&\quad \times \Bigg ( \int _{0}^{+\infty }\varphi _1^1(a)\text{ d }a\int _{0}^{+\infty }\varphi _2^2(a)\text{ d }a\int _{0}^{+\infty }\varphi _3^2(a)\text{ d }a +\int _{0}^{+\infty }\beta (a)\varphi _1^2(a)\text{ d }a\\&\quad \times \int _{0}^{+\infty }\varphi _2^2(a)\text{ d }a\int _{0}^{+\infty }\varphi _3^1(a)\text{ d }a\\&\quad +\int _{0}^{+\infty }\varphi _1^2(a)\text{ d }a\int _{0}^{+\infty }\varphi _2^1(a)\text{ d }a\int _{0}^{+\infty }\varphi _3^2(a)\text{ d }a\Bigg )\\&\quad +\frac{s(b\eta -2)}{b^3\eta ^3m_1^2\nu ^2}\Bigg ( \int _{0}^{+\infty }\varphi _1^1(a)\text{ d }a\int _{0}^{+\infty }\varphi _2^2(a)\text{ d }a\int _{0}^{+\infty }\beta (a)\varphi _3^1(a)\text{ d }a\\&\quad +\int _{0}^{+\infty }\varphi _1^1(a)\text{ d }a\int _{0}^{+\infty }\beta (a)\varphi _2^1(a)\text{ d }a\int _{0}^{+\infty }\varphi _3^2(a)\text{ d }a +\int _{0}^{+\infty }\beta (a)\varphi _1^1(a)\text{ d }a\\&\quad \times \int _{0}^{+\infty }\varphi _2^1(a)\text{ d }a\int _{0}^{\infty }\varphi _3^2(a)\text{ d }a\\&\quad +\int _{0}^{+\infty }\beta (a)\varphi _1^1(a)\text{ d }a\int _{0}^{+\infty }\varphi _2^2(a)\text{ d }a\int _{0}^{+\infty }\varphi _3^1(a)\text{ d }a\\&\quad +\int _{0}^{+\infty }\varphi _1^2(a)\text{ d }a\int _{0}^{+\infty }\varphi _2^1(a)\text{ d }a\\&\quad \times \int _{0}^{+\infty }\beta (a)\varphi _3^1(a)\text{ d }a +\int _{0}^{+\infty }\varphi _1^2(a)\text{ d }a\int _{0}^{+\infty }\beta (a)\varphi _2^1(a)\text{ d }a\int _{0}^{+\infty }\varphi _3^1(a)\text{ d }a\Bigg )\\&\quad +\frac{2(b\eta -1)}{b^3\eta ^3m_1\nu ^2}\Bigg ( \int _{0}^{+\infty }\beta (a)\varphi _1^1(a)\text{ d }a\int _{0}^{+\infty }\varphi _2^2(a)\text{ d }a\int _{0}^{+\infty }\varphi _3^2(a)\text{ d }a\\&\quad +\int _{0}^{+\infty }\varphi _1^2(a)\text{ d }a\\&\quad \times \int _{0}^{+\infty }\beta (a)\varphi _2^1(a)\text{ d }a\int _{0}^{+\infty }\varphi _3^2(a)\text{ d }a +\int _{0}^{+\infty }\varphi _1^2(a)\text{ d }a\int _{0}^{+\infty }\varphi _2^2(a)\text{ d }a\\&\quad \times \int _{0}^{+\infty }\beta (a)\varphi _3^1(a)\text{ d }a\Bigg )-\frac{6s\mu P_1}{b^4\eta ^4m_1^3\nu ^3}\\&\quad \times \Bigg ( \int _{0}^{+\infty }\varphi _1^1(a)\text{ d }a\int _{0}^{+\infty }\varphi _2^1(a)\text{ d }a\int _{0}^{+\infty }\varphi _3^1(a)\text{ d }a\Bigg )\\&\quad -\frac{6\mu (b\eta -1)P_1}{sb^4\eta ^4m_1\nu ^3}\Bigg ( \int _{0}^{+\infty }\varphi _1^2(a)\text{ d }a\int _{0}^{+\infty }\varphi _2^2(a)\text{ d }a\int _{0}^{+\infty }\varphi _3^2(a)\text{ d }a\Bigg )\\&\quad +\frac{2s\mu (3-2b\eta )P_1}{b^4\eta ^4m_1^2\nu ^3}\Bigg ( \int _{0}^{+\infty }\varphi _1^2(a)\text{ d }a\int _{0}^{+\infty }\varphi _2^1(a)\text{ d }a\int _{0}^{+\infty }\varphi _3^1(a)\text{ d }a\Bigg ), \end{aligned}$$

$$\begin{aligned}&\widehat{\overline{\varphi }}_3(\tau _k,\tilde{\tau })=(\tilde{\tau }_1+\tilde{\tau }_2+\tilde{\tau }_3)\Bigg [\frac{s\mu P_1}{(b\eta \nu m_1)^2} \left( \int _{0}^{+\infty }\overline{\overline{\varphi }}^1(a)\text{ d }a\right) ^2-\frac{\mu PP_1}{sb^2\eta ^2\nu ^2m_1}\\&\quad \times \left( \int _{0}^{+\infty }\overline{\overline{\varphi }}^2(a)\text{ d }a\right) ^2\\&\quad +\frac{\mu (2-b\eta )P_1}{b^2\eta ^2\nu ^2m_1}\left( \int _{0}^{+\infty }\overline{\overline{\varphi }}^1(a)\text{ d }a \int _{0}^{\infty }\overline{\overline{\varphi }}^2(a)\text{ d }a+\int _{0}^{+\infty }\overline{\overline{\varphi }}^2(a)\text{ d }a \int _{0}^{\infty }\overline{\overline{\varphi }}^1(a)\text{ d }a\right) \\&\quad +\int _{0}^{+\infty }f(a)\overline{\overline{\varphi }}^1(a)\text{ d }a\left( \frac{b\eta -1}{b^2\eta \nu }\int _{0}^{+\infty }\overline{\overline{\varphi }}^2(a)\text{ d }a- \frac{s}{b^2\eta \nu m_1}\int _{0}^{+\infty }\overline{\overline{\varphi }}^1(a)\text{ d }a\right) \Bigg ]\\&\quad +\frac{(\tilde{\tau }_2\tilde{\tau }_3+\tilde{\tau }_1\tilde{\tau }_3+\tilde{\tau }_1\tilde{\tau }_2)}{\tau _k}\Bigg [\left( \frac{\mu P_1^2(1-\tau _k\mu )}{(b\eta m_1\nu )^2} +\frac{\mu (2-b\eta )P_1(1-\tau _kd)}{b^2\eta ^2\nu m_1}\right) \\&\quad \times \int _{0}^{+\infty }\overline{\overline{\varphi }}^1(a)\text{ d }a+\left( \frac{(b\eta -1)(1-\tau _kd)}{b^2\eta }-\frac{P_1(1-\tau _k\mu )}{b^2\eta m_1\nu }\right) \int _{0}^{+\infty }f(a)\overline{\overline{\varphi }}^1(a)\text{ d }a\\&\quad +\left( \frac{\mu (2-b\eta )P_1^2(1-\tau _k\mu )}{sb^2\eta ^2\nu ^2m_1}-\frac{\mu PP_1(1-\tau _kd)}{sb^2\eta ^2\nu m_1}\right) \int _{0}^{+\infty }\overline{\overline{\varphi }}^2(a)\text{ d }a\Bigg ]\\&\quad +(\tilde{\tau }_2\tilde{\tau }_3+\tilde{\tau }_1\tilde{\tau }_3+\tilde{\tau }_1\tilde{\tau }_2)\Bigg [\left( \frac{\mu ^2P_1^2(\tau _k\mu -2)}{s(b\eta m_1\nu )^2} +\frac{\mu d(2-b\eta )P_1(\tau _kd-2)}{b^2\eta ^2\nu m_1}\right) \\&\quad \times \int _{0}^{+\infty }\overline{\overline{\varphi }}^1(a)\text{ d }a\\&\quad +\left( \frac{d(b\eta -1)(\tau _kd-2)}{b^2\eta }-\frac{\mu P_1(\tau _k\mu -2)}{sb^2\eta m_1\nu }\right) \int _{0}^{+\infty }f(a)\overline{\overline{\varphi }}^1(a)\text{ d }a\\&\quad +\left( \frac{\mu ^2(2-b\eta )P_1^2(\tau _k\mu -2)}{s^2b^2\eta ^2\nu ^2m_1}-\frac{\mu d PP_1(\tau _kd-2)}{sb^2\eta ^2\nu m_1}\right) \int _{0}^{+\infty }\overline{\overline{\varphi }}^2(a)\text{ d }a\Bigg ]\\&\quad +\tau _k\Bigg \{-\frac{6s^2\mu P_1}{(b\eta m_1\nu )^3}\left( \int _{0}^{+\infty }\overline{\overline{\varphi }}^1(a)\text{ d }a\right) ^3 -\frac{6\mu (b\eta -1)P_1}{sb^3\eta ^3\nu ^3}\left( \int _{0}^{+\infty }\overline{\overline{\varphi }}^2(a)\text{ d }a\right) ^3\\&\quad +\frac{2\mu (2b\eta -3)P_1}{b^3\eta ^3m_1\nu ^3}\Bigg [ \int _{0}^{+\infty }\overline{\overline{\varphi }}^1(a)\text{ d }a\left( \int _{0}^{+\infty }\overline{\overline{\varphi }}^2(a)\text{ d }a\right) ^2 +\int _{0}^{+\infty }\overline{\overline{\varphi }}^2(a)\text{ d }a\\&\quad \times \int _{0}^{+\infty }\overline{\overline{\varphi }}^1(a)\text{ d }a\int _{0}^{+\infty }\overline{\overline{\varphi }}^2(a)\text{ d }a +\left( \int _{0}^{+\infty }\overline{\overline{\varphi }}^2(a)\text{ d }a\right) ^2\int _{0}^{+\infty }\overline{\overline{\varphi }}^1(a)\text{ d }a\Bigg ]\\&\quad +\frac{2s\mu (b\eta -3)P_1}{b^3\eta ^3m_1^2\nu ^3}\Bigg [ \int _{0}^{+\infty }\overline{\overline{\varphi }}^1(a)\text{ d }a\int _{0}^{+\infty }\overline{\overline{\varphi }}^2(a)\text{ d }a\int _{0}^{+\infty }\overline{\overline{\varphi }}^1(a)\text{ d }a\\&\quad +\left( \int _{0}^{+\infty }\overline{\overline{\varphi }}^1(a)\text{ d }a\right) ^2\int _{0}^{+\infty }\overline{\overline{\varphi }}^2(a)\text{ d }a\Bigg ] -\frac{s(b\eta -2)}{b^3\eta ^2m_1\nu ^2} \Bigg [\int _{0}^{+\infty }f(a)\overline{\overline{\varphi }}^1(a)\text{ d }a\\&\quad \times \int _{0}^{+\infty }\overline{\overline{\varphi }}^1(a)\text{ d }a\int _{0}^{+\infty }\overline{\overline{\varphi }}^2(a)\text{ d }a +\int _{0}^{+\infty }f(a)\overline{\overline{\varphi }}^1(a)\text{ d }a\int _{0}^{+\infty }\overline{\overline{\varphi }}^2(a)\text{ d }a\\&\quad \times \int _{0}^{+\infty }\overline{\overline{\varphi }}^1(a)\text{ d }a\Bigg ]\\&\quad +\frac{2s^2}{b^3(\eta m_1\nu )^2} \Bigg [\int _{0}^{\infty }f(a)\overline{\overline{\varphi }}^1(a)\text{ d }a\left( \int _{0}^{+\infty }\overline{\overline{\varphi }}^1(a)\text{ d }a\right) ^2\Bigg ]\\&\quad -\frac{2(b\eta -1)}{b^3\eta ^2\nu ^2} \Bigg [\int _{0}^{\infty }f(a)\overline{\overline{\varphi }}^1(a)\text{ d }a\\&\quad \times \left( \int _{0}^{+\infty }\overline{\overline{\varphi }}^2(a)\text{ d }a\right) ^2\Bigg ] -\frac{2s\mu (2b\eta -3)P_1}{b^3\eta ^3m_1^2\nu ^3}\\&\quad \times \Bigg [\int _{0}^{+\infty }\overline{\overline{\varphi }}^2(a)\text{ d }a\left( \int _{0}^{+\infty }\overline{\overline{\varphi }}^1(a)\text{ d }a\right) ^2\Bigg ]\Bigg \}\\&\quad +(\tilde{\tau }_1+\tilde{\tau }_2+\tilde{\tau }_3)\Bigg \{-\frac{6s\mu P_1^2(1-\tau _k\mu )}{(b\eta m_1\nu )^3}\left( \int _{0}^{+\infty }\overline{\overline{\varphi }}^1(a)\text{ d }a\right) ^2\\&\quad +\left[ \frac{2\mu (2b\eta -3)P_1^2(1-\tau _k\mu )}{sb^3\eta ^3(1-m)\nu ^3}-\frac{6\mu (b\eta -1)P_1(1-\tau _kd)}{sb^3\eta ^3\nu ^2}\right] \left( \int _{0}^{+\infty }\overline{\overline{\varphi }}^2(a)\text{ d }a\right) ^2\\&\quad +\frac{2\mu (2b\eta -3)P_1(1-\tau _kd)}{b^3\eta ^3m_1\nu ^2}\Big ( \int _{0}^{+\infty }\overline{\overline{\varphi }}^1(a)\text{ d }a\int _{0}^{+\infty }\overline{\overline{\varphi }}^2(a)\text{ d }a +\int _{0}^{+\infty }\overline{\overline{\varphi }}^2(a)\text{ d }a\\&\quad \times \int _{0}^{+\infty }\overline{\overline{\varphi }}^1(a)\text{ d }a\Big ) +\frac{2\mu (b\eta -3)P_1^2(1-\tau _k\mu )}{b^3\eta ^3m_1^2\nu ^3} \int _{0}^{+\infty }\overline{\overline{\varphi }}^1(a)\text{ d }a\int _{0}^{+\infty }\overline{\overline{\varphi }}^2(a)\text{ d }a\\&\quad +\frac{2s\mu (b\eta -3)P_1(1-\tau _kd)}{b^3\eta ^3m_1^2\nu ^2}\left( \int _{0}^{+\infty }\overline{\overline{\varphi }}^1(a)\text{ d }a\right) ^2\\&\quad +\left[ \frac{2sP_1(1-\tau _k\mu )}{b^3(\eta m_1\nu )^2}-\frac{s(b\eta -2)(1-\tau _kd)}{b^3\eta ^2m_1\nu }\right] \int _{0}^{+\infty }f(a)\overline{\overline{\varphi }}^1(a)\text{ d }a\int _{0}^{+\infty }\overline{\overline{\varphi }}^1(a)\text{ d }a\\&\quad -\left[ \frac{s(b\eta -2)P_1(1-\tau _k\mu )}{b^3\eta ^2m_1\nu }-\frac{2(b\eta -1)(1-\tau _kd)}{b^3\eta ^2\nu }\right] \int _{0}^{+\infty }f(a)\overline{\overline{\varphi }}^1(a)\text{ d }a\\&\quad \times \int _{0}^{+\infty }\overline{\overline{\varphi }}^2(a)\text{ d }a -\frac{2\mu (2b\eta -3)P_1^2(1-\tau _k\mu )}{b^3\eta ^3m_1^2\nu ^3} \int _{0}^{+\infty }\overline{\overline{\varphi }}^2(a)\text{ d }a\int _{0}^{+\infty }\overline{\overline{\varphi }}^1(a)\text{ d }a\Bigg \}\\&\quad +\frac{(\tilde{\tau }_2\tilde{\tau }_3+\tilde{\tau }_1\tilde{\tau }_3+\tilde{\tau }_1\tilde{\tau }_2)}{\tau _k}\Bigg \{ \Bigg [\frac{4\mu (b\eta -3)P_1^2(1-\tau _k\mu )(1-\tau _kd)}{b^3\eta ^3m_1^2\nu ^2}-\frac{6\mu P_1^3(1-\tau _k\mu )^2}{(b\eta m_1\nu )^3}\\&\quad +\frac{2\mu (2b\eta -3)P_1(1-\tau _kd)^2}{b^3\eta ^3m_1\nu }\Bigg ]\int _{0}^{+\infty }\overline{\overline{\varphi }}^1(a)\text{ d }a +\Bigg [\frac{4\mu (2b\eta -3)P_1^2(1-\tau _k\mu )(1-\tau _kd)}{sb^3\eta ^3m_1\nu ^2}\\&\quad -\frac{6\mu (b\eta -1)P_1(1-\tau _kd)^2}{sb^3\eta ^3} -\frac{2\mu (2b\eta -3)P_1^2(1-\tau _k\mu )^2}{sb^3\eta ^3m_1^2\nu ^3}\Bigg ]\int _{0}^{+\infty }\overline{\overline{\varphi }}^2(a)\text{ d }a\\&\quad +\frac{2P_1(1-\tau _k\mu )[P_1(1-\tau _k\mu )-m_1\nu (b\eta -2)(1-\tau _kd)]}{b^3(\eta m_1\nu )^2}\int _{0}^{+\infty }f(a)\overline{\overline{\varphi }}^1(a)\text{ d }a\\&\quad -\frac{2(b\eta -1)(1-\tau _kd)^2}{b^3\eta ^2}\int _{0}^{+\infty }f(a)\overline{\overline{\varphi }}^1(a)\text{ d }a\Bigg \},\\ \end{aligned}$$

$$\begin{aligned}&\widehat{\overline{\varphi }}_4(\tau _k,\tilde{\tau })=(\tilde{\tau }_1+\tilde{\tau }_2\\&\quad +\tilde{\tau }_3)\Bigg [-\frac{\mu sP_1}{b^3\eta ^3\nu ^2m_1^2} \left( \int _{0}^{\infty }\overline{\overline{\varphi }}^1(a)\text{ d }a\right) ^2+\frac{s}{b^2\eta ^2\nu m_1} \int _{0}^{\infty }\beta (a)\overline{\overline{\varphi }}^1(a)\text{ d }a\\&\quad \times \int _{0}^{+\infty }\overline{\overline{\varphi }}^1(a)\text{ d }a +\frac{b\eta -1}{b^2\eta ^2\nu }\int _{0}^{+\infty }\beta (a)\overline{\overline{\varphi }}^1(a)\text{ d }a\int _{0}^{+\infty }\overline{\overline{\varphi }}^2(a)\text{ d }a\\&\quad +\frac{K \mu (b\eta -1)P_1- s r b^3\eta ^3\nu ^2m_1}{Ksb^3\eta ^3\nu ^2m_1} \left( \int _{0}^{+\infty }\overline{\overline{\varphi }}^2(a)\text{ d }a\right) ^2-\frac{\mu (2-b\eta )P_1}{b^3\eta ^3\nu ^2m_1}\\&\quad \times \left( \int _{0}^{+\infty }\overline{\overline{\varphi }}^1(a)\text{ d }a \int _{0}^{+\infty }\overline{\overline{\varphi }}^2(a)\text{ d }a+\int _{0}^{+\infty }\overline{\overline{\varphi }}^2(a)\text{ d }a \int _{0}^{+\infty }\overline{\overline{\varphi }}^1(a)\text{ d }a\right) \Bigg ]\\&\quad +\frac{(\tilde{\tau }_2\tilde{\tau }_3+\tilde{\tau }_1\tilde{\tau }_3+\tilde{\tau }_1\tilde{\tau }_2)}{\tau _k}\Bigg [-\left( \frac{\mu P_1^2(1-\tau _k\mu )}{b^3\eta ^3m_1^2\nu ^2} +\frac{\mu (2-b\eta )P_1(1-\tau _kd)}{b^3\eta ^3\nu m_1}\right) \\&\quad \times \int _{0}^{+\infty }\overline{\overline{\varphi }}^1(a)\text{ d }a+\left( \frac{(b\eta -1)(1-\tau _kd)}{b^2\eta ^2}+\frac{P_1^2(1-\tau _k\mu )}{b^2\eta ^2m_1\nu }\right) \int _{0}^{+\infty }\beta (a)\overline{\overline{\varphi }}^1(a)\text{ d }a\\&\quad +\frac{[K\mu (b\eta -1)P_1-s r b^3\eta ^3\nu ^2m_1](1-\tau _kd)}{Ksb^3\eta ^3\nu m_1}\int _{0}^{+\infty }\overline{\overline{\varphi }}^2(a)\text{ d }a\\&\quad -\frac{\mu (2-b\eta )P_1^2(1-\tau _k\mu )}{sb^3\eta ^3\nu ^2m_1}\int _{0}^{+\infty }\overline{\overline{\varphi }}^2(a)\text{ d }a\Bigg ]\\&\quad +(\tilde{\tau }_2\tilde{\tau }_3+\tilde{\tau }_1\tilde{\tau }_3+\tilde{\tau }_1\tilde{\tau }_2)\Bigg [-\left( \frac{\mu ^2P_1^3(\tau _k\mu -2)}{s b^3\eta ^3(1-m)^2\nu ^2} +\frac{\mu d(2-b\eta )P_1(1-\tau _kd)}{b^3\eta ^3\nu m_1}\right) \\&\quad \times \int _{0}^{+\infty }\overline{\overline{\varphi }}^1(a)\text{ d }a+\left( \frac{d(b\eta -1)(\tau _kd-2)}{b^2\eta ^2}+\frac{\mu P_1^3(\tau _k\mu -2)}{sb^3\eta ^2m_1\nu }\right) \int _{0}^{+\infty }\beta (a)\overline{\overline{\varphi }}^1(a)\text{ d }a\\&\quad +\frac{[K\mu (b\eta -1)P_1-s rb^3\eta ^3\nu ^2m_1](1-\tau _kd)d}{Ksb^3\eta ^3\nu m_1}\int _{0}^{+\infty }\overline{\overline{\varphi }}^2(a)\text{ d }a\\&\quad -\frac{\mu ^2(2-b\eta )P_1^3(\tau _k\mu -2)}{s^2b^3\eta ^3\nu ^2m_1}\int _{0}^{+\infty }\overline{\overline{\varphi }}^2(a)\text{ d }a\Bigg ]\\&\quad +\tau _k\Bigg \{-\frac{6s\mu (P\nu -\alpha )}{b^4\eta ^4m_1^3\nu ^3}\left( \int _{0}^{+\infty }\overline{\overline{\varphi }}^1(a)\text{ d }a\right) ^3 -\frac{6\mu (b\eta -1)(P\nu -\alpha )}{s b^4\eta ^4m_1\nu ^3}\\&\quad \times \left( \int _{0}^{+\infty }\overline{\overline{\varphi }}^2(a)\text{ d }a\right) ^3+\frac{2\mu (3-2b\eta )P_1}{b^4\eta ^4m_1^2\nu ^3}\Bigg [ \int _{0}^{+\infty }\overline{\overline{\varphi }}^1(a)\text{ d }a\left( \int _{0}^{+\infty }\overline{\overline{\varphi }}^2(a)\text{ d }a\right) ^2\\ {}&\quad +\int _{0}^{+\infty }\overline{\overline{\varphi }}^2(a)\text{ d }a\int _{0}^{+\infty }\overline{\overline{\varphi }}^1(a)\text{ d }a\\&\quad \times \int _{0}^{+\infty }\overline{\overline{\varphi }}^2(a)\text{ d }a\Bigg ] +\frac{2s\mu (3-2b\eta )P_1}{b^4\eta ^4m_1^2\nu ^3}\left( \int _{0}^{+\infty }\overline{\overline{\varphi }}^2(a)\text{ d }a\right) ^2\int _{0}^{+\infty }\overline{\overline{\varphi }}^1(a)\text{ d }a\\&\quad +\frac{2\mu (3-b\eta )P_1}{b^4\eta ^4m_1^2\nu ^3}\Bigg [ \int _{0}^{+\infty }\overline{\overline{\varphi }}^1(a)\text{ d }a\int _{0}^{+\infty }\overline{\overline{\varphi }}^2(a)\text{ d }a\int _{0}^{+\infty }\overline{\overline{\varphi }}^1(a)\text{ d }a\\&\quad +\left( \int _{0}^{+\infty }\overline{\overline{\varphi }}^1(a)\text{ d }a\right) ^2\\&\quad \times \int _{0}^{+\infty }\overline{\overline{\varphi }}^2(a)\text{ d }a\Bigg ] +\frac{s(b\eta -2)}{b^3\eta ^3m_1^2\nu ^2} \Bigg [\int _{0}^{+\infty }\beta (a)\overline{\overline{\varphi }}^1(a)\text{ d }a\int _{0}^{+\infty }\overline{\overline{\varphi }}^1(a)\text{ d }a \int _{0}^{+\infty }\overline{\overline{\varphi }}^2(a)\text{ d }a\\&\quad +\int _{0}^{+\infty }\beta (a)\overline{\overline{\varphi }}^1(a)\text{ d }a\int _{0}^{+\infty }\overline{\overline{\varphi }}^2(a)\text{ d }a\int _{0}^{+\infty }\overline{\overline{\varphi }}^1(a)\text{ d }a\Bigg ] -\frac{2s^2}{b^3\eta ^3(1-m)^2\nu ^3}\\&\quad \times \Bigg [\int _{0}^{+\infty }\beta (a)\overline{\overline{\varphi }}^1(a)\text{ d }a \left( \int _{0}^{+\infty }\overline{\overline{\varphi }}^1(a)\text{ d }a\right) ^2\Bigg ] +\frac{2(b\eta -1)}{b^3\eta ^3m_1\nu ^2} \Bigg [\int _{0}^{+\infty }\beta (a)\overline{\overline{\varphi }}^1(a)\text{ d }a\\&\quad \times \left( \int _{0}^{+\infty }\overline{\overline{\varphi }}^2(a)\text{ d }a\right) ^2\Bigg ]+\frac{2s\mu (3-2b\eta )P_1}{b^4\eta ^4m_1^2\nu ^3} \Bigg [\int _{0}^{+\infty }\overline{\overline{\varphi }}^2(a)\text{ d }a\left( \int _{0}^{+\infty }\overline{\overline{\varphi }}^1(a)\text{ d }a\right) ^2\Bigg ]\Bigg \}\\&\quad +(\tilde{\tau }_1+\tilde{\tau }_2+\tilde{\tau }_3)\Bigg \{-\frac{6\mu P_1^2(1-\tau _k\mu )}{b^4\eta ^4m_1^3\nu ^3}\left( \int _{0}^{+\infty }\overline{\overline{\varphi }}^1(a)\text{ d }a\right) ^2\\ {}&\quad +\Bigg [\frac{2\mu (3-2b\eta )P_1^2(1-\tau _k\mu )}{b^4\eta ^4m_1^2\nu ^3}\\&\quad -\frac{6\mu (b\eta -1)P_1(1-\tau _kd)}{s b^4\eta ^4m_1\nu ^2}\Bigg ]\left( \int _{0}^{+\infty }\overline{\overline{\varphi }}^2(a)\text{ d }a\right) ^2 +\frac{2\mu (3-2b\eta )P_1(1-\tau _kd)}{b^4\eta ^4m_1^2\nu ^2}\\&\quad \times \Bigg ( \int _{0}^{+\infty }\overline{\overline{\varphi }}^1(a)\text{ d }a\int _{0}^{+\infty }\overline{\overline{\varphi }}^2(a)\text{ d }a +\int _{0}^{+\infty }\overline{\overline{\varphi }}^2(a)\text{ d }a \int _{0}^{+\infty }\overline{\overline{\varphi }}^1(a)\text{ d }a\Bigg )\\&\quad +\frac{2\mu (3-b\eta )P_1^2(1-\tau _k\mu )}{sb^4\eta ^4m_1^2\nu ^3} \int _{0}^{+\infty }\overline{\overline{\varphi }}^1(a)\text{ d }a\int _{0}^{+\infty }\overline{\overline{\varphi }}^2(a)\text{ d }a\\ {}&\quad +\frac{2\mu (3-b\eta )P_1(1-\tau _kd)}{b^4\eta ^4m_1^2\nu ^2}\\&\quad \times \left( \int _{0}^{+\infty }\overline{\overline{\varphi }}^1(a)\text{ d }a\right) ^2 +\left[ \frac{s(b\eta -2)(1-\tau _kd)}{b^3\eta ^3m_1^2\nu }-\frac{2sP_1(1-\tau _k\mu )}{b^3\eta ^3m_1^2\nu ^3}\right] \\ {}&\quad \int _{0}^{+\infty }\beta (a)\overline{\overline{\varphi }}^1(a)\text{ d }a\\&\quad \times \int _{0}^{+\infty }\overline{\overline{\varphi }}^1(a)\text{ d }a+\frac{2\mu (3-2b\eta )P_1^2(1-\tau _k\mu )}{b^4\eta ^4m_1^2\nu ^3} \int _{0}^{+\infty }\overline{\overline{\varphi }}^2(a)\text{ d }a\int _{0}^{+\infty }\overline{\overline{\varphi }}^1(a)\text{ d }a\\&\quad +\left[ \frac{(b\eta -2)P_1(1-\tau _k\mu )}{b^3\eta ^3m_1^2\nu ^2}+\frac{2(b\eta -1)(1-\tau _kd)}{b^3\eta ^3m_1\nu }\right] \\ {}&\quad \int _{0}^{+\infty }\beta (a)\overline{\overline{\varphi }}^1(a)\text{ d }a\int _{0}^{+\infty }\overline{\overline{\varphi }}^2(a)\text{ d }a\Bigg \}\\&\quad +\frac{(\tilde{\tau }_2\tilde{\tau }_3+\tilde{\tau }_1\tilde{\tau }_3+\tilde{\tau }_1\tilde{\tau }_2)}{\tau _k}\Bigg \{ \Bigg [\frac{4\mu (3-b\eta )P_1^2(1-\tau _k\mu )(1-\tau _kd)}{sb^4\eta ^4m_1^2\nu }\\ {}&\quad -\frac{6\mu P_1^3(1-\tau _k\mu )^2}{sb^4\eta ^4m_1^3\nu ^3}\\&\quad +\frac{2\mu (3-2b\eta )P_1(1-\tau _kd)^2}{b^4\eta ^4m_1^2\nu }\Bigg ]\int _{0}^{+\infty }\overline{\overline{\varphi }}^1(a)\text{ d }a\\ {}&\quad +\Bigg [\frac{4\mu (3-2b\eta )P_1^2(1-\tau _k\mu )(1-\tau _kd)}{sb^4\eta ^4m_1^2\nu ^2}\\&\quad -\frac{6\mu (b\eta -1)P_1(1-\tau _kd)^2}{sb^4\eta ^4m_1\nu } +\frac{2\mu (3-2b\eta )P_1^3(1-\tau _k\mu )^2}{sb^4\eta ^4m_1^2\nu }\Bigg ]\int _{0}^{+\infty }\overline{\overline{\varphi }}^2(a)\text{ d }a\\&\quad +\Bigg [\frac{2(b\eta -1)(1-\tau _kd)^2}{b^3\eta ^3m_1}-\frac{2P_1^2(1-\tau _k\mu )^2}{b^3\eta ^3m_1^2\nu ^3}\Bigg ]\int _{0}^{+\infty }f(a)\overline{\overline{\varphi }}^1(a)\text{ d }a\\&\quad +\frac{2(b\eta -2)P_1(1-\tau _k\mu )(1-\tau _kd)}{b^3\eta ^3m_1^2\nu }\int _{0}^{+\infty }f(a)\overline{\overline{\varphi }}^1(a)\text{ d }a\Bigg \}. \end{aligned}$$

## Appendix A.2

The formulas $\ell _i(\tau _k),~i=1,2,3,4,5,6,$ were used in Sect. 5.4.

$$\begin{aligned} \ell _1(\tau _k)=&-\frac{\mu P_1}{b\eta m_1\nu (\omega _*i+\tau _k\mu )}+\frac{\tau _k}{\omega _*i+\tau _k\mu }\\ {}&\left[ \frac{2\mu P_1^2(1-\tau _k\mu )}{(b\eta m_1\nu )^2}+ \frac{\mu (2-b\eta )P_1(1-\tau _kd)}{b^2\eta ^2\nu m_1}\right] \\&\quad +\frac{b\mu e^{-\omega _*i}}{\omega _*i+\tau _k\mu }+\frac{\tau _kb\mu e^{-\omega _*i}}{\omega _*i+\tau _k\mu }\left[ \frac{(b\eta -1)(1-\tau _kd)}{b^2\eta }-\frac{P_1(1-\tau _k\mu )}{b\eta m_1\nu }\right] \\&\quad +\frac{\mu PP_1}{sb\eta m_1\nu (\omega _*i+\tau _kd)} +\frac{\tau _k}{\omega _*i+\tau _kd}\\&\quad \times \left[ \frac{\mu (2-b\eta )P_1^2(1-\tau _k\mu )}{sb^2\eta ^2\nu ^2m_1} -\frac{2\mu PP_1(1-\tau _kd)}{sb^2\eta ^2\nu m_1}\right] ,\\ \ell _2(\tau _k)&=-\frac{\mu P_1}{b^2\eta ^2m_1\nu (\omega _*i+\tau _k\mu )}-\frac{\mu e^{-\omega _*i}}{b\eta (\omega _*i+\tau _k\mu )}\\&\quad +\frac{r K\eta ^2\nu bsm_1-2r\eta ^2\nu ^2bsm_1-\mu KPP_1}{K b^2\eta ^2\nu sm_1(\omega _*i+\tau _kd)}\\&\quad +\frac{\tau _k}{\omega _*i+\tau _k\mu }\left[ \frac{2\mu P_1^2(1-\tau _k\mu )}{sb^3\eta ^3m_1^2\nu ^2}+ \frac{\mu (b\eta -2)P_1(1-\tau _kd)}{b^2\eta ^2\nu m_1}\right] \\&\quad +\frac{\tau _k\mu e^{-\omega _*i}}{\omega _*i+\tau _k\mu }\left[ \frac{P_1(1-\tau _k\mu )}{b^2\eta ^2m_1\nu }-\frac{(b\eta -1)(1-\tau _kd)}{b^2\eta ^2\nu }\right] \\&\quad +\frac{\tau _k}{\omega _*i+\tau _kd}\left[ \frac{2[K\mu PP_1-s rb^3\eta ^3\nu ^2m_1^2](1-\tau _kd)}{Ksb^3\eta ^3m_1^2}\right. \\&\quad \left. -\frac{\mu (2-b\eta )P_1^2(1-\tau _k\mu )}{sb^3\eta ^3\nu ^2m_1}\right] \\ \ell _3(\tau _k)&=\frac{\tau _k}{(\omega _*i+\tau _k\mu )^2}\left[ \frac{2s\mu P_1}{(b\eta \nu m_1)^2}- \frac{s\mu e^{-\omega _*i}}{b\eta \nu m_1}\right] +\frac{\tau _k\mu PP_1}{sb^2\eta ^2\nu ^2m_1(\omega _*i+\tau _kd)^2}\\&\quad +\frac{\tau _k}{(\omega _*i+\tau _k\mu )(\omega _*i+\tau _kd)}\left[ \frac{b\eta -1}{b^2\eta \nu }+\frac{2\mu (2-b\eta )P_1}{b^2\eta ^2\nu ^2m_1} \right] ,\\ \ell _4(\tau _k)&=\frac{\tau _k}{(\omega _*i+\tau _k\mu )^2}\left[ \frac{s\mu e^{-\omega _*i}}{b^2\eta ^2\nu ^2m_1}- \frac{s\mu P_1}{b^3\eta ^3\nu ^2m_1^2}\right] \\&\quad +\frac{2\tau _k[K \mu (b\eta -1)P_1- s r b^3\eta ^3\nu ^2m_1]}{Ksb^3\eta ^3\nu ^2m_1(\omega _*i+\tau _kd)^2}\\&\quad +\frac{\tau _k}{(\omega _*i+\tau _k\mu )(\omega _*i+\tau _kd)}\\&\quad \times \left[ \frac{\mu (b\eta -2)P_1}{b^2\eta ^2\nu ^2m_1}\left( 1+\frac{1}{b\eta }\right) -\frac{(b\eta -1)\mu e^{-\omega _*i}}{b^2\eta ^2\nu }\right] , \end{aligned}$$

$$\begin{aligned} \ell _5(\tau _k)&=\frac{2\tau _ks\mu m_1}{[b\eta \nu m_1]^2(\omega _*i+\tau _k\mu )(-\omega _*i+\tau _k\mu )}\\&\quad +\frac{\tau _k\mu (2-b\eta )P_1}{b^2\eta ^2\nu ^2m_1(\omega _*i+\tau _k\mu )(-\omega _*i+\tau _kd)}\\&\quad -\frac{\tau _k s\mu e^{-\omega _*i}}{b\eta \nu m_1(\omega _*i+\tau _k\mu )(-\omega _*i+\tau _kd)}+ \frac{\tau _k(b\eta -1)\mu e^{-\omega _*i}}{b\eta (\omega _*i+\tau _k\mu )(-\omega _*i+\tau _kd)}\\&\quad +\frac{\tau _k\mu (2-b\eta )P_1}{b^2\eta ^2\nu ^2m_1(\omega _*i+\tau _kd)(-\omega _*i+\tau _k\mu )}\\&\quad - \frac{2\tau _k\mu PP_1}{sb^2\eta ^2\nu ^2m_1(\omega _*i+\tau _kd)(-\omega _*i+\tau _kd)},\\ \ell _6(\tau _k)&=-\frac{2\tau _ks\mu P_1}{b^3\eta ^3\nu ^2m_1^2(\omega _*i+\tau _k\mu )(-\omega _*i+\tau _k\mu )}\\&\quad -\frac{\tau _k\mu (2-b\eta )P_1\mu e^{-\omega _*i}}{b^3\eta ^3\nu ^2m_1(\omega _*i+\tau _k\mu )(-\omega _*i+\tau _kd)}\\&\quad +\frac{\tau _k s\mu e^{-\omega _*i}}{b^2\eta ^2\nu m_1(\omega _*i+\tau _k\mu )(-\omega _*i+\tau _k\mu )}\\&\quad +\frac{\tau _k(b\eta -1)\mu e^{-\omega _*i}}{b^2\eta ^2\nu (\omega _*i+\tau _k\mu )(-\omega _*i+\tau _kd)}\\&\quad +\frac{2\tau _k[K \mu (b\eta -1)P_1- s r b^3\eta ^3\nu ^2m_1]}{Ksb^3\eta ^3\nu ^2m_1(\omega _*i+\tau _kd)(-\omega _*i+\tau _kd)}\\&\quad - \frac{\tau _k\mu (2-b\eta )P_1}{b^3\eta ^3\nu ^2m_1(\omega _*i+\tau _kd)(-\omega _*i+\tau _k\mu )}. \end{aligned}$$

## Appendix A.3

The formulas $\digamma _1,~\digamma _2,~\ell _{20},~\ell _{11},~\ell _{02}$ and $\mathcal {Q}$ were used in Sect. 5.5.

$$\begin{aligned} \digamma _1&=\left( \frac{\text{ d }~\text{ det }(\Delta (\omega _*i))}{\text{ d }\lambda }\right) ^{-1} \Bigg \{-\frac{\mu P_1}{b\eta m_1\nu (\omega _*i+\tau _k\mu )}\left( 1+\frac{1}{b\eta }\right) \\&\quad +\frac{\mu e^{-\omega _*i}}{\omega _*i+\tau _k\mu }\left( b-\frac{1}{b\eta }\right) \\&\quad +\frac{1}{\omega _*i+\tau _kd}\left[ \frac{\mu PP_1}{sb\eta m_1\nu }+ \frac{r K\eta ^2\nu bs-2r\eta ^2\nu ^2bs-\mu K(b\eta -1)P_1}{K b^2\eta ^2\nu s}\right] \\&\quad +\frac{\tau _k}{\omega _*i+\tau _k\mu }\left[ \frac{2\mu P_1^2(1-\tau _k\mu )}{(b\eta m_1\nu )^2}\left( 1+\frac{1}{sb\eta }\right) + \frac{\mu (2-b\eta )P_1(1-\tau _kd)}{b^2\eta ^2\nu m_1}\right] \\&\quad +\frac{\tau _k\mu e^{-\omega _*i}}{\omega _*i+\tau _k\mu }\left[ \frac{(b\eta -1)(1-\tau _kd)}{b^2\eta }\left( 1-\frac{1}{\eta \nu }\right) -\frac{P_1(1-\tau _k\mu )}{b\eta m_1\nu }\left( 1-\frac{1}{b\eta }\right) \right] \\&\quad +\frac{2\tau _k(1-\tau _kd)}{\omega _*i+\tau _kd}\left[ \frac{2[K\mu (b\eta -1)P_1-s rb^3\eta ^3\nu ^2m_1]}{Ksb^3\eta ^3m_1}-\frac{\mu (b\eta -1)P_1}{sb^2\eta ^2\nu }\right] \\&\quad +\frac{\tau _k(1-\tau _k\mu )}{\omega _*i+\tau _kd}\left[ \frac{\mu (2-b\eta )P_1^2}{sb^2\eta ^2\nu ^2m_1}\left( 1-\frac{1}{b\eta }\right) \right] \Bigg \},\\ \digamma _2&=\left( \frac{\text{ d }~\text{ det }(\Delta (\omega _*i))}{\text{ d }\lambda }\right) ^{-1} \Bigg \{-\frac{\mu P_1}{b\eta m_1\nu (-\omega _*i+\tau _k\mu )}\left( 1+\frac{1}{b\eta }\right) \\ {}&\quad +\frac{\mu e^{-\omega _*i}}{-\omega _*i+\tau _k\mu }\left( b-\frac{1}{b\eta }\right) \\&\quad +\frac{1}{-\omega _*i+\tau _kd}\left[ \frac{\mu PP_1}{sb\eta m_1\nu }+ \frac{r K\eta ^2\nu bs-2r\eta ^2\nu ^2bs-\mu K(b\eta -1)P_1}{K b^2\eta ^2\nu s}\right] \\&\quad +\frac{\tau _k}{-\omega _*i+\tau _k\mu }\left[ \frac{2\mu P_1^2(1-\tau _k\mu )}{(b\eta m_1 \nu )^2}\left( 1+\frac{1}{sb\eta }\right) + \frac{\mu (2-b\eta )P_1(1-\tau _kd)}{b^2\eta ^2\nu m_1}\right] \\&\quad +\frac{\tau _k\mu e^{-\omega _*i}}{-\omega _*i+\tau _k\mu }\left[ \frac{(b\eta -1)(1-\tau _kd)}{b^2\eta }\left( 1-\frac{1}{\eta \nu }\right) -\frac{P_1(1-\tau _k\mu )}{b\eta m_1\nu }\left( 1-\frac{1}{b\eta }\right) \right] \\&\quad +\frac{2\tau _k(1-\tau _kd)}{-\omega _*i+\tau _kd}\left[ \frac{2[K\mu (b\eta -1)P_1-s rb^3\eta ^3\nu ^2m_1]}{Ksb^3\eta ^3m_1}-\frac{\mu (b\eta -1)P_1}{sb^2\eta ^2\nu }\right] \\&\quad +\frac{\tau _k(1-\tau _k\mu )}{-\omega _*i+\tau _kd}\left[ \frac{\mu (2-b\eta )P_1^2}{sb^2\eta ^2\nu ^2m_1}\left( 1-\frac{1}{b\eta }\right) \right] \Bigg \}, \end{aligned}$$

$$\begin{aligned} \ell _{20}&=\frac{\tau _k}{(\omega _*i+\tau _k\mu )^2}\left[ \frac{s\mu e^{-\omega _*i}}{b^2\eta ^2\nu ^2m_1}\left( 1-b\eta \nu \right) +\frac{2s\mu P_1}{(b\eta \nu m_1)^2}\left( 1-\frac{1}{2b\eta }\right) \right] \\&\quad +\frac{\tau _k}{(\omega _*i+\tau _kd)^2}\left[ \frac{\mu PP_1}{sb^2\eta ^2\nu ^2m_1}-\frac{K \mu (b\eta -1)P_1- s r b^3\eta ^3\nu ^2m_1}{Ksb^3\eta ^3\nu ^2m_1}\right] \\&\quad +\frac{\tau _k}{(\omega _*i+\tau _k\mu )(\omega _*i+\tau _kd)}\left[ \frac{\mu (b\eta -2)P_1}{b^2\eta ^2\nu ^2m_1} \left( \frac{1}{b\eta }-1\right) \right. \\ {}&\quad \left. +\frac{b\eta -1}{b^2\eta \nu }\left( 1-\frac{\mu e^{-\omega _*i}}{\eta }\right) \right] ,\\ \ell _{11}&=\frac{\tau _k}{(\omega _*i+\tau _k\mu )(-\omega _*i+\tau _k\mu )}\left[ \frac{2s\mu P_1}{(b\eta \nu m_1)^2}\left( 1-\frac{1}{b\eta }\right) +\frac{s\mu e^{-\omega _*i}}{b^2\eta ^2\nu m_1}\right] \\&\quad +\frac{\tau _k}{(\omega _*i+\tau _k\mu )(-\omega _*i+\tau _kd)}\left[ \frac{\mu (2-b\eta )P_1}{b^2\eta ^2\nu ^2m_1} \left( 1-\frac{\mu e^{-\omega _*i}}{b\eta }\right) \right] \\&\quad -\frac{\tau _k\mu e^{-\omega _*i}}{(\omega _*i+\tau _k\mu )(-\omega _*i+\tau _kd)}\left[ \frac{s}{b\eta \nu m_1}-\frac{b\eta -1}{b\eta }+\frac{b\eta -1}{b^2\eta ^2\nu }\right] \\&\quad +\frac{\tau _k}{(\omega _*i+\tau _kd)(-\omega _*i+\tau _k\mu )}\left[ \frac{\mu (2-b\eta )P_1}{b^2\eta ^2\nu ^2m_1} \left( 1-\frac{1}{b\eta }\right) \right] \\&\quad +\frac{2\tau _k}{(\omega _*i+\tau _kd)(-\omega _*i+\tau _kd)}\left[ \frac{K \mu (b\eta -1)P_1 - s r b^3\eta ^3\nu ^2m_1}{Ksb^3\eta ^3\nu ^2m_1}-\frac{\mu PP_1}{sb^2\eta ^2\nu ^2m_1}\right] ,\\ \ell _{02}&=\frac{\tau _k}{(-\omega _*i+\tau _k\mu )^2}\left[ \frac{s\mu e^{-\omega _*i}}{b^2\eta ^2\nu ^2m_1}\left( 1-b\eta \nu \right) +\frac{2s\mu P_1}{(b\eta \nu m_1)^2}\left( 1-\frac{1}{2b\eta }\right) \right] \\&\quad +\frac{\tau _k}{(-\omega _*i+\tau _kd)^2}\left[ \frac{\mu PP_1}{sb^2\eta ^2\nu ^2m_1}-\frac{K \mu (b\eta -1)P_1- s r b^3\eta ^3\nu ^2m_1}{Ksb^3\eta ^3\nu ^2m_1}\right] \\&\quad +\frac{\tau _k}{(-\omega _*i+\tau _k\mu )(-\omega _*i+\tau _kd)}\left[ \frac{\mu (b\eta -2)P_1}{b^2\eta ^2\nu ^2m_1} \left( \frac{1}{b\eta }-1\right) \right. \\&\quad \left. +\frac{b\eta -1}{b^2\eta \nu }\left( 1-\frac{\mu e^{-\omega _*i}}{\eta }\right) \right] ,\\ \end{aligned}$$

$$\begin{aligned} \mathcal {Q}&= \tau _k\Bigg \{-\frac{6s^2\mu P_1}{(b\eta m_1\nu )^3(\omega _*i+\tau _k\mu )^2(\omega _*i+\tau _k\mu )}\\&\quad -\frac{6\mu (b\eta -1)P_1}{sb^3\eta ^3\nu ^3(\omega _*i+\tau _kd)^2(\omega _*i+\tau _kd)}+\frac{2\mu (2b\eta -3)P_1}{b^3\eta ^3m_1\nu ^3}\\&\quad \times \Bigg [ \frac{1}{(\omega _*i+\tau _kd)^2(-\omega _*i+\tau _k\mu )}+\frac{2}{(\omega _*i+\tau _k\mu )(\omega _*i+\tau _kd)(-\omega _*i+\tau _kd)}\Bigg ]\\&\quad +\frac{2s\mu (b\eta -3)P_1}{b^3\eta ^3m_1^2\nu ^3}\\&\times \quad \Bigg [ \frac{1}{(\omega _*i+\tau _k\mu )^2(-\omega _*i+\tau _kd)}+\frac{2}{(\omega _*i+\tau _kd)(\omega _*i+\tau _k\mu )(-\omega _*i+\tau _k\mu )}\Bigg ]\\&\quad -\frac{s\mu (b\eta -2)}{b^3\eta ^2m_1\nu ^2}\\&\quad \times \Bigg [\frac{e^{-\omega _*i}}{(\omega _*i+\tau _k\mu )^2(-\omega _*i+\tau _kd)}+\frac{2e^{-\omega _*i}}{(\omega _*i+\tau _kd)(\omega _*i+\tau _k\mu )(-\omega _*i+\tau _k\mu )}\Bigg ]\\&\quad -\frac{2\mu (b\eta -1)}{b^3\eta ^2\nu ^2}\\&\quad \times \Bigg [\frac{e^{-\omega _*i}}{(\omega _*i+\tau _kd)^2(-\omega _*i+\tau _k\mu )}+\frac{2e^{-\omega _*i}}{(\omega _*i+\tau _k\mu )(\omega _*i+\tau _kd)(-\omega _*i+\tau _kd)}\Bigg ]\\&\quad +\frac{2s^2\mu e^{-\omega _*i}}{(b\eta m_1\nu )^2(\omega _*i+\tau _k\mu )^2(\omega _*i+\tau _k\mu )}\Bigg \}\\&\quad +\frac{\mu (2-b\eta )P_1}{b^2\eta ^2\nu ^2m_1(\omega _*i+\tau _k\mu )}\Bigg [-\frac{\ell _{11}}{\omega _*i(\omega _*i+\tau _kd)}+\frac{\overline{\ell }_{11}}{\omega _*i(-\omega _*i+\tau _kd)}\\&\quad +2\int _{0}^{+\infty }\widetilde{\varphi }_{116}(\tau _k)(a)\text{ d }a\Bigg ]\\&\quad +\frac{\mu (2-b\eta )P_1}{b^2\eta ^2\nu ^2m_1(-\omega _*i+\tau _k\mu )}\Bigg [\frac{\ell _{20}}{\omega _*i(\omega _*i+\tau _kd)}+\frac{\overline{\ell }_{02}}{3\omega _*i(-\omega _*i+\tau _kd)}\\&\quad +\int _{0}^{+\infty }\widetilde{\varphi }_{114}(\tau _k)(a)\text{ d }a\Bigg ]\\&\quad +\frac{\mu (2-b\eta )P_1}{b^2\eta ^2\nu ^2m_1(\omega _*i+\tau _k\mu )}\Bigg [-\frac{\ell _{11}}{\omega _*i(\omega _*i+\tau _k\mu )}+\frac{\overline{\ell }_{11}}{\omega _*i(-\omega _*i+\tau _k\mu )}\\&\quad +2\int _{0}^{+\infty }\widetilde{\varphi }_{115}(\tau _k)(a)\text{ d }a\Bigg ]\\&\quad +\frac{\mu (2-b\eta )P_1}{b^2\eta ^2\nu ^2m_1(-\omega _*i+\tau _k\mu )}\Bigg [\frac{\ell _{20}}{\omega _*i(\omega _*i+\tau _k\mu )}+\frac{\overline{\ell }_{02}}{3\omega _*i(-\omega _*i+\tau _k\mu )}\\&\quad +\int _{0}^{+\infty }\widetilde{\varphi }_{113}(\tau _k)(a)\text{ d }a\Bigg ]\\&\quad +\frac{2s\mu P_1}{(b\eta \nu m_1)^2(\omega _*i+\tau _k\mu )}\Bigg [-\frac{\ell _{11}}{\omega _*i(\omega _*i+\tau _k\mu )}+\frac{\overline{\ell }_{11}}{\omega _*i(-\omega _*i+\tau _k\mu )}\\&\quad +2\int _{0}^{+\infty }\widetilde{\varphi }_{115}(\tau _k)(a)\text{ d }a\Bigg ]\\&\quad +\frac{2s\mu P_1}{(b\eta \nu m_1)^2(-\omega _*i+\tau _k\mu )}\Bigg [\frac{\ell _{20}}{\omega _*i(\omega _*i+\tau _k\mu )}+\frac{\overline{\ell }_{02}}{3\omega _*i(-\omega _*i+\tau _k\mu )}\\&\quad +\int _{0}^{+\infty }\widetilde{\varphi }_{113}(\tau _k)(a)\text{ d }a\Bigg ]\\&\quad -\frac{2\mu PP_1}{sb^2\eta ^2\nu ^2m_1(\omega _*i+\tau _k\mu )}\Bigg [-\frac{\ell _{11}}{\omega _*i(\omega _*i+\tau _kd)}+\frac{\overline{\ell }_{11}}{\omega _*i(-\omega _*i+\tau _kd)}\\&\quad +2\int _{0}^{+\infty }\widetilde{\varphi }_{116}(\tau _k)(a)\text{ d }a\Bigg ]\\&\quad -\frac{2\mu PP_1}{sb^2\eta ^2\nu ^2m_1(-\omega _*i+\tau _k\mu )}\Bigg [\frac{\ell _{20}}{\omega _*i(\omega _*i+\tau _kd)}+\frac{\overline{\ell }_{02}}{3\omega _*i(-\omega _*i+\tau _kd)}\\&\quad +\int _{0}^{+\infty }\widetilde{\varphi }_{114}(\tau _k)(a)\text{ d }a\Bigg ]\\&\quad -\frac{s\mu e^{-\omega _*i}}{b\eta \nu m_1(\omega _*i+\tau _k\mu )}\Bigg [-\frac{\ell _{11}}{\omega _*i(\omega _*i+\tau _k\mu )}+\frac{\overline{\ell }_{11}}{\omega _*i(-\omega _*i+\tau _k\mu )}\\&\quad +2\int _{0}^{+\infty }\widetilde{\varphi }_{115}(\tau _k)(a)\text{ d }a\Bigg ]\\&\quad -\frac{s\mu e^{\omega _*i}}{b\eta \nu m_1(-\omega _*i+\tau _k\mu )}\Bigg [\frac{\ell _{20}}{\omega _*i(\omega _*i+\tau _k\mu )}+\frac{\overline{\ell }_{02}}{3\omega _*i(-\omega _*i+\tau _k\mu )}\\&\quad +\int _{0}^{+\infty }\widetilde{\varphi }_{113}(\tau _k)(a)\text{ d }a\Bigg ]\\&\quad +\frac{(\eta -1)\mu e^{-\omega _*i}}{b\eta \nu m_1(\omega _*i+\tau _k\mu )}\Bigg [-\frac{\ell _{11}}{\omega _*i(\omega _*i+\tau _kd)}+\frac{\overline{\ell }_{11}}{\omega _*i(-\omega _*i+\tau _kd)}\\&\quad +2\int _{0}^{+\infty }\widetilde{\varphi }_{116}(\tau _k)(a)\text{ d }a\Bigg ]\\&\quad +\frac{(\eta -1)\mu e^{\omega _*i}}{b\eta \nu m_1(-\omega _*i+\tau _k\mu )}\Bigg [\frac{\ell _{20}}{\omega _*i(\omega _*i+\tau _kd)}+\frac{\overline{\ell }_{02}}{3\omega _*i(-\omega _*i+\tau _kd)}\\&\quad +\int _{0}^{+\infty }\widetilde{\varphi }_{114}(\tau _k)(a)\text{ d }a\Bigg ]. \end{aligned}$$
